# Genome-Wide Identification of the Target Genes of AP2-O, a *Plasmodium* AP2-Family Transcription Factor

**DOI:** 10.1371/journal.ppat.1004905

**Published:** 2015-05-27

**Authors:** Izumi Kaneko, Shiroh Iwanaga, Tomomi Kato, Issei Kobayashi, Masao Yuda

**Affiliations:** 1 Department of Medical Zoology, Mie University Graduate School of Medicine, Tsu, Mie, Japan; 2 Core-Lab, Graduate School of Regional Innovation Studies, Mie University, Tsu, Mie, Japan; University of Geneva, SWITZERLAND

## Abstract

Stage-specific transcription is a fundamental biological process in the life cycle of the *Plasmodium* parasite. Proteins containing the AP2 DNA-binding domain are responsible for stage-specific transcriptional regulation and belong to the only known family of transcription factors in *Plasmodium* parasites. Comprehensive identification of their target genes will advance our understanding of the molecular basis of stage-specific transcriptional regulation and stage-specific parasite development. AP2-O is an AP2 family transcription factor that is expressed in the mosquito midgut-invading stage, called the ookinete, and is essential for normal morphogenesis of this stage. In this study, we identified the genome-wide target genes of AP2-O by chromatin immunoprecipitation-sequencing and elucidate how this AP2 family transcription factor contributes to the formation of this motile stage. The analysis revealed that AP2-O binds specifically to the upstream genomic regions of more than 500 genes, suggesting that approximately 10% of the parasite genome is directly regulated by AP2-O. These genes are involved in distinct biological processes such as morphogenesis, locomotion, midgut penetration, protection against mosquito immunity and preparation for subsequent oocyst development. This direct and global regulation by AP2-O provides a model for gene regulation in *Plasmodium* parasites and may explain how these parasites manage to control their complex life cycle using a small number of sequence-specific AP2 transcription factors.

## Author Summary

Although malarial parasites have a complex life cycle, they harbor only 30 transcription factors in their genome. The majority of these transcription factors belong to a single family referred to as the AP2 family. Our previous study suggested that stage-specific AP2 family transcription factors have critical roles in maintaining the *Plasmodium* parasite life cycle. However, it remains fairly elusive as to how these transcription factors regulate each stage. AP2-O is an AP2 family transcription factor that is expressed during the mosquito midgut-invading stage, the ookinete, and is essential for normal development of this stage. In the present study, we identified the entire set of AP2-O target genes to elucidate how this AP2 family transcription factor contributes to the formation of this stage. Our results showed that AP2-O directly regulates 10% of the parasite genome and is involved in the whole process of mosquito midgut-invasion by ookinetes. The global and comprehensive regulation by the AP2 family transcription factor that we revealed provides a model for transcriptional regulation of this parasite and may explain how malarial parasites regulate their complex life cycle using a small number of sequence-specific transcription factors.

## Introduction

Malarial parasites require two host animals during their life cycle and undergo multiple developmental changes in each host. According to these changes in the life cycle, parasites remarkably alter their repertoire of gene expression [1]. However, the corresponding regulatory mechanisms of gene expression remain poorly understood. In contrast to the lifecycle, malarial parasites have only a small set of sequence-specific transcription factors in their genome. The majority of the transcription factors belong to a single transcription factor family known as the Apetala2 (AP2) family, and 26–27 genes in this family have been detected in the genome [2,3]. The total number of sequence-specific transcription factors is exceptionally small compared with that in other eukaryotic organisms [4–6], suggesting that malaria parasites have a unique gene regulation system. Previous studies by us and other groups suggest that AP2-family transcription factors are involved in stage-specific gene regulation and are essential for normal development of the stages in which they are expressed [7–11]. However, only partial information has been obtained about their target genes; thus, it remains elusive how these transcription factors contribute to the development of each stage.

Ookinetes are motile forms of malarial parasites that are generated in the midgut of a mosquito after ingestion of an infected blood meal. The ookinetes promptly invade midgut epithelial cells and arrive at the basal lamina. There, they transform into oocysts, in which sporozoites, the liver-invading form, develop. We reported previously that the AP2-O AP2-family transcription factor is expressed in developing ookinetes of *Plasmodium berghei* [7]. Targeting experiments demonstrated that the disruptants display abnormal morphologies and completely lose infectivity to mosquitoes. We explored AP2-O targets by microarray analysis and identified 19 genes as targets. They included genes that encode microneme proteins and major surface proteins [7]. However, these genes do not explain the major abnormal morphogenesis phenotype of AP2-O disruptants, suggesting that most targets of this transcription factor remain to be identified.

The aim of this study was to investigate the basic features of transcriptional regulation in malaria parasites through elucidating the role of *P*. *berghei* AP2-O in this motile stage; determining the types of target genes controlled by the transcription factor and the extent to which they are responsible for gene regulation in this stage. We performed chromatin immunoprecipitation-high-throughput sequencing (ChIP-seq) and determined the whole range of *P*. *berghei* AP2-O target genes [12,13]. The results revealed that AP2-O regulates hundreds of genes directly and oversees the transcriptional regulation of this stage as a master regulator. Based on this result, we discuss the possibility that this centralized gene regulatory system represents a basic feature of transcriptional regulation in malarial parasites and could explain the paucity of transcription factors in this parasite.

## Results

### AP2-O has more than 1,000 binding sites on the genome

**Fig 1:**
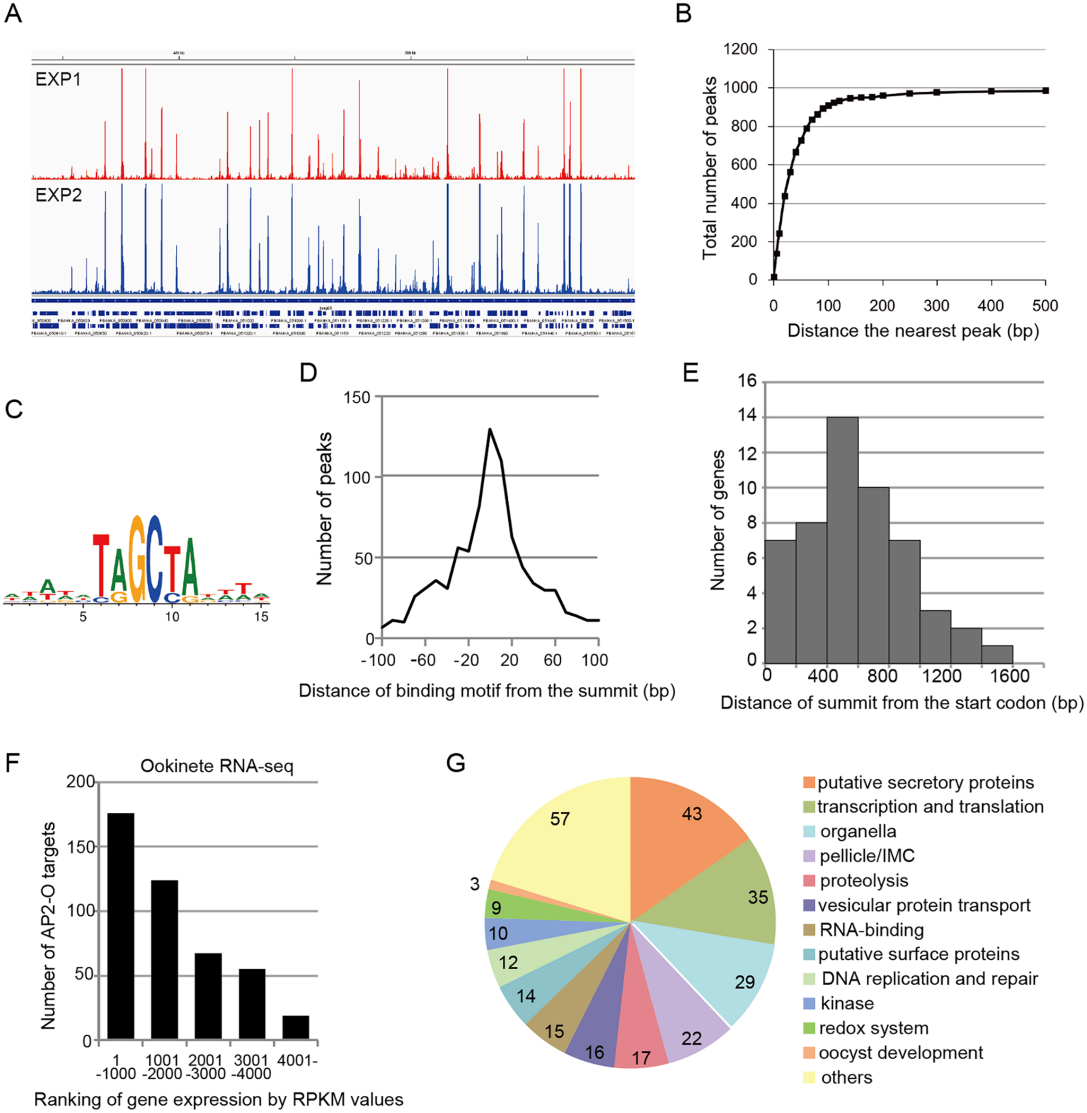
AP2-O targets over 500 genes in the genome. A. ChIP-seq experiments were performed independently using two different sequence platforms: an Illumina Genome analyzer and an ABI SOLiD 5500 system. This figure shows AP2-O peaks in each experiment within a 200-kb region (350–550-kb) of the fifth chromosome. The Integrative Genomic Viewer [56] was used for generating this image from bedgraph files (S ChIP-seq bedgraph). Peak call was performed with the MACS2 program [57], and 1,540 and 1,111 peaks were identified (FDR < 0.01, fold enrichments > 5). B. Distance of each peak in experiment 2 (1,111 peaks) to the nearest peak in experiment 1 (1,570 peaks) was calculated. Numbers of peaks that have a matching peak within the selected distance were plotted. The graph indicates that nearly 90% of peaks in experiment 2 have counterparts in those in experiment 1. C. Sequences enriched around the summits of AP2-O peaks. Logos were generated using WebLogo 3.3 (http://weblogo.threeplusone.com/create.cgi) [58]. D. Distances between the predicted summits of the AP2-O peaks and the motif sequences. The horizontal axis indicates the distance from the summit to the nearest motif sequence (the bin size is 20-bp). The vertical axis indicates the number of peaks in each region (total number of peaks = 959). E. The distribution of AP2-O peaks in the upstream regions of the target genes. The horizontal axis indicates the distance between the summits of the AP2-O peaks and the first methionine codon. The data were obtained from 53 putative target genes identified using microarray analysis. F. The *P*. *berghei* genes were divided into six groups containing 1,000 genes each (except for the 6^th^ group, which contained 900 genes) according to RPKM values estimated by RNA-seq. The number of target genes in each group is shown as a histogram. The horizontal axis indicates the groups ordered according to their expression levels. G. A pie chart showing the functional categories of target genes (282 genes in total). Hypothetical protein genes are not shown. The number of members in each group is shown on the chart.

ChIP-seq was performed with transgenic *P*. *berghei* that expressed green fluorescent protein (GFP)-tagged AP2-O, using ChIP conditions established previously for ChIP-quantitative polymerase chain reaction analysis in ookinetes [7]. Two independent ChIP-seq analyses were performed, and the data were compared to confirm experimental reproducibility. The comparison showed that the results were quite reproducible (Fig 1A). Approximately 90% of the significantly-enriched peaks of the second experiment were found within 90-bp from a significantly-enriched peak in the first experiment (Fig 1B), suggesting that these peaks are common to both. The numbers of target genes predicted in the two experiments were 541 and 573, and 465 genes were common to both (S1 and S2 Tables in S1 File). In a subsequent analysis, we primarily used the results of the second experiment, which was performed more recently with a next-generation sequencing platform that is higher in both read length and read number.

Analysis of the second set of ChIP-seq data with the MACS2 peak-calling program identified 1,111 peaks [false discovery rate (FDR) < 0.01 and fold enrichment over input > 5] [14]. Regions located within 100 base pairs (bp) of the predicted summit of each peak were sorted from the genome to identify the AP2-O-binding sequences. DNA sequences appearing frequently in these regions were analyzed using Fisher’s exact test (S3 and S4 Tables in S1 File). When six-base sequences were ranked according to their p-values, 17 of the top 20 sequences overlapped, thereby yielding the [TC][AG]GC[TC][AG] binding motif. This was the same motif predicted in our previous study [7] (Fig 1C), and 86% of the peaks had this motif within the peak region. Fig 1D summarizes the distances between the summits of these peaks and the binding motif. These results confirm that the motif predicted in our previous study is used for AP2-O binding *in vivo*. We further investigated the remaining 14% of the peaks that did not contain this motif. Statistical analysis using these peaks showed that another motif sequence, TG[ATC]ACA, was highly concentrated around the summit (S5 and S6 Tables in S1 File). The two nucleotides on either end of this motif, viz. TG and CA, were the same as those in the primary motif, suggesting that they are minor variants of the binding motif. After including those sequences, 99% of the peaks (1,097 of 1,111) had either motif within their regions. The distances of these motifs from the predicted summits averaged 26.6-bp.

### Target gene prediction revealed over 500 putative target genes

Next, the positional relationship of the binding sites with the target was investigated to predict the target genes based on the binding sites. Our previous study suggested that AP2-O binding sequences are usually located in the 1-kb upstream regions of the target genes, on the basis of data of 19 genes that were mainly (15 of 19 genes) identified by microarray analysis between wild-type parasites and AP2-O disruptants [7]. However, the number of genes seemed too small to define the relationship (the array was designed on our expressed sequence tag data). Accordingly, to identify more candidates as targets, we performed another microarray analysis using an array designed on the *P*. *berghei* genome sequence [11]. In that analysis, we identified 63 genes as candidate AP2-O targets (a twofold decrease in the AP2-O disruptants was observed). The 63 genes contained all 15 genes identified in the previous study. The ChIP-seq data showed that 53 of these genes had AP2-O peaks in the upstream intergenic region (Table 1). Among these genes, approximately 90% of the AP2-O peaks (49/53) existed within a 1,200-bp region upstream of the start codon and most frequently at 400–600-bp (Fig 1E). Based on this result, we concluded that it was appropriate to define the predicted target genes as those with AP2-O binding sites within the 1,200-bp region upstream of the start codon. However, this result indicated that target genes would be missed in such a prediction when they harbored AP2-O binding sites further upstream of this 1,200-bp region. Thus, in the following study, the targets were further manually investigated using the ChIP-seq and high-throughput cDNA sequencing (RNA-seq) data (See also Table 1, Table 2, and S7 Table in S1 File).

**Table 1:** List of genes whose expression decreased in *AP2-O*(–) ookinetes.

Gene ID	KO/WT	target	functional annotation
PBANKA_110690	0.00696	Yes	conserved Plasmodium protein, unknown function
PBANKA_100640	0.0107	Yes	conserved Plasmodium protein, unknown function
PBANKA_111920	0.0128	Yes	conserved Plasmodium protein, unknown function
PBANKA_060960	0.0159	Yes	heat shock protein 20, putative
PBANKA_080050	0.0171	Yes	chitinase (CHT1)
PBANKA_103780	0.0209	Yes	secreted ookinete adhesive protein (SOAP)
PBANKA_145770	0.0265	Yes	conserved Plasmodium protein, unknown function
PBANKA_041290	0.0361	Yes	circumsporozoite- and TRAP-related protein (CTRP)
PBANKA_114370	0.039	Yes	secreted ookinete protein, putative (PSOP2)
PBANKA_070660	0.0444	Yes	conserved Plasmodium protein, unknown function
PBANKA_070650	0.0568	Yes	exonuclease, putative
PBANKA_135340	0.069	Yes	secreted ookinete protein, putative (PSOP7)
PBANKA_101700	0.0721	Yes	CorA-like Mg2 transporter protein, putative
PBANKA_041065	0.0786	Yes	conserved Plasmodium protein, unknown function
PBANKA_120070	0.0899	Yes	conserved Plasmodium protein, unknown function
PBANKA_121810	0.0989	Yes	oocyst capsule protein (Cap380)
PBANKA_122890	0.105	Yes	von willebrand factor a-domain-related protein (WARP)
PBANKA_124140	0.117	Yes	conserved Plasmodium protein, unknown function
PBANKA_100620	0.146	No	sporozoite invasion-associated protein 1 (SIAP1)
PBANKA_081020	0.162	Yes	conserved Plasmodium protein, unknown function
PBANKA_122540	0.17	Yes	conserved Plasmodium protein, unknown function
PBANKA_020170	0.227	Yes	conserved Plasmodium protein, unknown function
PBANKA_136270	0.233	Yes	conserved Plasmodium protein, unknown function
PBANKA_122150	0.242	Yes	mitochondrial ribosomal protein L1 precursor, putative
PBANKA_082100	0.269	Yes*	chaperone protein, putative
PBANKA_133660	0.271	Yes	conserved Plasmodium protein, unknown function
PBANKA_083240	0.276	Yes	conserved Plasmodium protein, unknown function
PBANKA_113810	0.281	Yes	14-3-3 protein, putative
PBANKA_090980	0.284	Yes*	conserved Plasmodium protein, unknown function
PBANKA_145080	0.324	Yes	conserved Plasmodium protein, unknown function
PBANKA_071830	0.333	Yes	DNA replication licensing factor, putative
PBANKA_010640	0.34	Yes	conserved Plasmodium protein, unknown function
PBANKA_110760	0.346	Yes	6-cysteine protein (P38)
PBANKA_145670	0.356	Yes	conserved Plasmodium protein, unknown function
PBANKA_040820	0.37	Yes	calcium dependent protein kinase 3 (CDPK3)
PBANKA_071140	0.371	Yes	perforin like protein 4 (PPLP4)
PBANKA_123370	0.372	Yes	conserved Plasmodium protein, unknown function
PBANKA_122270	0.388	Yes	conserved Plasmodium protein, unknown function
PBANKA_145090	0.4	Yes*	conserved Plasmodium protein, unknown function
PBANKA_020410	0.408	No	N-terminal acetyltransferase, putative
PBANKA_082420	0.408	Yes	perforin like protein 3 (PPLP3)
PBANKA_051810	0.41	Yes	conserved Plasmodium protein, unknown function
PBANKA_140920	0.41	Yes	conserved Plasmodium protein, unknown function
PBANKA_112890	0.418	Yes*	conserved Plasmodium protein, unknown function
PBANKA_122460	0.423	Yes*	conserved Plasmodium protein, unknown function
PBANKA_123280	0.426	Yes	conserved Plasmodium protein, unknown function
PBANKA_111370	0.427	Yes	tubulin-tyrosine ligase, putative
PBANKA_030220	0.435	Yes*	dynein light chain, putative
PBANKA_103640	0.444	Yes	BOP1-like protein, putative
PBANKA_061650	0.449	Yes	conserved Plasmodium protein, unknown function
PBANKA_091600	0.454	Yes	conserved Plasmodium protein, unknown function
PBANKA_145910	0.46	Yes	conserved Plasmodium protein, unknown function
PBANKA_071450	0.46	Yes	conserved Plasmodium protein, unknown function
PBANKA_082450	0.463	Yes*	lipoate-protein ligase a type 2, putative (LplA2)
PBANKA_040360	0.465	Yes*	conserved Plasmodium protein, unknown function
PBANKA_120990	0.467	Yes*	conserved Plasmodium protein, unknown function
PBANKA_100650	0.475	Yes	peptidase, M22 family, putative
PBANKA_061920	0.484	Yes	secreted ookinete protein, putative (PSOP1)
PBANKA_120890	0.485	No	conserved Plasmodium protein, unknown function
PBANKA_031320	0.486	No	conserved Plasmodium protein, unknown function
PBANKA_146000	0.49	No	rac-beta serine/threonine protein kinase, putative (PKB)
PBANKA_143230	0.492	Yes	cell traversal protein for ookinetes and sporozoites (CelTOS)
PBANKA_131310	0.498	Yes	conserved Plasmodium protein, unknown function

Microarray analysis was performed between wild-type and *AP2-O*(–) parasites. Genes whose expression was decreased over two-fold are listed. Asterisks indicate genes that have peaks over 1200-bp upstream, in the intron, or in the exon of adjacent genes. Target genes identified in experiment 2 are indicated by “Yes” in the third column.

**Table 2:** Gene expression analysis of *P*. *berghei* ookinetes by RNA-seq.

	Gene ID	RPKMs	functional annotation	Array	target
1	PBANKA_103780	272813.96	secreted ookinete adhesive protein (SOAP)	Yes	Yes
2	PBANKA_051500	59476.6	25 kDa ookinete surface antigen precursor (P25)	No	Yes
3	PBANKA_051490	58297.98	28 kDa ookinete surface protein (P28)	No	Yes
4	PBANKA_094180	23344.68	histone H2B, putative (H2B)	No	Yes
5	PBANKA_020170	21695.64	conserved Plasmodium protein, unknown function	Yes	Yes
6	PBANKA_122890	20589.64	von willebrand factor a-domain-related protein (WARP)	Yes	Yes
7	PBANKA_143230	19699.16	cell traversal protein for ookinetes and sporozoites (CelTOS)	Yes	Yes
8	PBANKA_122540	18119.61	conserved Plasmodium protein, unknown function	Yes	Yes
9	PBANKA_111920	17569.81	conserved Plasmodium protein, unknown function	Yes	Yes
10	PBANKA_145950	16481.06	myosin light chain 1, putative,myosin A tail domain interacting protein MTIP, putative (MTIP)	No	Yes
11	PBANKA_094190	14571.11	histone H4, putative	No	No
12	PBANKA_102440	12647.41	calmodulin, putative	No	Yes*
13	PBANKA_120990	11293.31	conserved Plasmodium protein, unknown function	Yes	Yes*
14	PBANKA_071290	10841.18	high mobility group protein, putative (HMGB2)	No	Yes*
15	PBANKA_111700	8537.87	histone H2A, putative (H2A)	No	No
16	PBANKA_080050	7604.15	chitinase (CHT1)	Yes	Yes
17	PBANKA_082020	7226.9	thioredoxin, putative	No	Yes
18	PBANKA_121760	7075.98	histone H2A variant, putative (H2A.Z)	No	No
19	PBANKA_140920	6992	conserved Plasmodium protein, unknown function	Yes	Yes
20	PBANKA_040770	6860.29	60S acidic ribosomal protein P2, putative	No	No
21	PBANKA_142060	6809.58	histone H2B, putative	No	No
22	PBANKA_131860	5902.24	conserved Plasmodium protein, unknown function	No	Yes
23	PBANKA_133680	5504.03	conserved Plasmodium protein, unknown function	No	Yes
24	PBANKA_135450	5395.46	60S ribosomal protein L18-2, putative	No	No
25	PBANKA_134900	5313.19	MSP7-like protein (MSRP2)	No	No
26	PBANKA_100640	4792.68	conserved Plasmodium protein, unknown function	Yes	Yes
27	PBANKA_140070	4721.6	conserved rodent malaria protein, unknown function	No	No
28	PBANKA_092670	4690.25	circumsporozoite-related antigen	No	No
29	PBANKA_111530	4661.24	glideosome-associated protein 40, putative (GAP40)	No	Yes
30	PBANKA_111710	4649.11	histone H3, putative (H3.3)	No	No
31	PBANKA_081900	4422.92	secreted acid phosphatase, putative,glideosome-associated protein 50, putative (GAP50)	No	Yes*
32	PBANKA_132500	4400.8	40S ribosomal protein S28e, putative	No	No
33	PBANKA_010880	4302.64	histone H3, putative	No	Yes
34	PBANKA_020160	4194.84	early transcribed membrane protein (ETRAMP)	No	No
35	PBANKA_031060	4016.99	conserved protein, unknown function	No	Yes
36	PBANKA_101850	3987.2	transcription factor 3b, putative	No	No
37	PBANKA_031450	3975.81	40S ribosomal protein S26e, putative	No	No
38	PBANKA_143760	3729.09	glideosome-associated protein 45, putative	No	Yes
39	PBANKA_020460	3512.56	photosensitized INA-labeled protein 1, putative	No	Yes
40	PBANKA_094360	3391.27	60S acidic ribosomal protein, putative	No	No
41	PBANKA_123400	3361.92	vacuolar ATP synthetase, putative	No	Yes
42	PBANKA_091810	3343.25	60S ribosomal protein L38e, putative	No	No
43	PBANKA_040540	3180.18	40S ribosomal protein S12, putative	No	No
44	PBANKA_145670	3135.76	conserved Plasmodium protein, unknown function	Yes	Yes
45	PBANKA_030130	3128.73	conserved Plasmodium protein, unknown function	No	No
46	PBANKA_101770	2963.2	ubiquinol-cytochrome c reductase hinge protein, putative	No	No
47	PBANKA_146130	2959.01	conserved Plasmodium protein, unknown function	No	Yes
48	PBANKA_071680	2920.24	conserved Plasmodium protein, unknown function	No	Yes
49	PBANKA_134670	2899.07	60S ribosomal protein L23, putative	No	No
50	PBANKA_061780	2869.53	conserved Plasmodium protein, unknown function	No	Yes

RNA-seq analysis was performed in *P*. *berghei* ookinetes cultured for 24 h, and genes were ordered according to reads per kilobase of coding sequence per million reads (RPKM) value. Asterisks indicate genes that have peaks over 1200-bp upstream or in the exon of adjacent genes.

According to the prediction rule defined above, 541 genes (approximately 10% of all *P*. *berghei* genes) were predicted to be AP2-O target genes (S1 Table in S1 File). These predicted target genes (later target genes) included 18 of the 19 target genes identified in our previous study [7], and almost all genes that have been reported to be ookinete-specific genes or to be important in midgut-infection of the parasite, as descried later.

Of the remaining 10 genes that did not show AP2-O peaks in the upstream intergenic region, one gene showed a peak in the first intron and four genes, which all harbored a short upstream intergenic region, showed a peak in the exon of the adjacent gene (S1 Fig). All these peaks accompanied the transcripts downstream. These results suggest that exons and introns are occasionally used as promoters in *Plasmodium*. However, AP2-O summits on the exons and introns were not used for predicting target genes in subsequent analyses because whether such cryptic transcripts would be finally translated into proteins with biological functions was unclear.

### AP2-O target genes in the ookinete transcriptome and proteome

An RNA-seq analysis was performed to investigate the expression levels of AP2-O target genes in the ookinete transcriptome. As summarized in a histogram (Fig 1F), targets were biased towards highly expressed genes. Among the 50 genes showing the highest number of reads per kilobase of coding sequence per million reads (RPKM), 38 genes were AP2-O targets (Table 2), suggesting that AP2-O contributes to a stage-specific gene expression pattern in ookinetes. It is notable that the majority of the AP2-O target genes within these 50 genes were not predicted to be targets by microarray analysis (Table 2). This was probably because a high background of transcripts carried from the female gametocytes (e.g., *p28* transcripts) made it difficult to predict targets using expression differences [7,15]. This clearly shows that ChIP-seq has an advantage in target prediction.

Proteomics data are now available for the ookinete stage [1,16–18]. These data provide a useful resource for exploring genes involved in midgut invasion [19]. We compared the list of AP2-O target genes with the data of microneme proteome which contains 330 proteins (S8 Table in S1 File) [16]. Microneme proteome contained six known microneme proteins, two microneme proteins newly identified in this proteome study, and six putative secreted ookinete proteins (PSOP). ChIP-seq identified 11 of these 14 proteins, and only three proteins were missed in the list of AP2-O targets. On the other hand, the list of AP2-O targets contains two genes that encode the membrane-attack ookinete protein (MAOP) [or perforin-like protein 3 (PPLP3)] and PPLP5. These proteins are not present in the microneme proteome (S7 and S8 Tables in S1 File). These results indicate that the target gene data are as comprehensive as the proteome data. The proteome data contain a number of proteins specific to other stages, including eight rhoptry proteins, five merozoite surface proteins, and eight proteins exported to the erythrocyte (21 proteins in total). These contaminants were probably included because it is difficult to completely remove blood stage parasites from ookinete samples by using differences in density. This means that ookinete proteome data inevitably contain contaminants from other stages. In clear contrast, the list of AP2-O targets contained none of these proteins (S8 Table in S1 File). This lack of contamination from other stages may result from the fact that ChIP-seq analysis is based solely on information about the binding of a stage-specific transcription factor.

### Overview of target gene functions

A total of 541 target genes were identified in the analyses described above. In addition, as described later, one novel gene was identified as a target gene in the genomic region that had been thought to be intergenic. All 542 genes were classified into several major groups, and this classification was based on their functional annotations in PlasmoDB (http://PlasmoDB.org), their corresponding structures (such as an N-terminal signal sequence), as well as similarities to functionally annotated genes in other apicomplexan parasites (Fig 1G and S7 Table in S1 File). This classification showed that the targets included several genes related to the morphogenesis of, and midgut invasion by ookinetes, together with genes involved in general functions such as translation, transcription, and DNA replication. In the following sections, we describe the target genes belonging to the categories related to midgut invasion and further explore novel members of these categories among the target genes using the list of identified targets as a resource for gene exploration.

### Exploration for possible missing genes in the *P*. *berghei* genome

**Fig 2:**
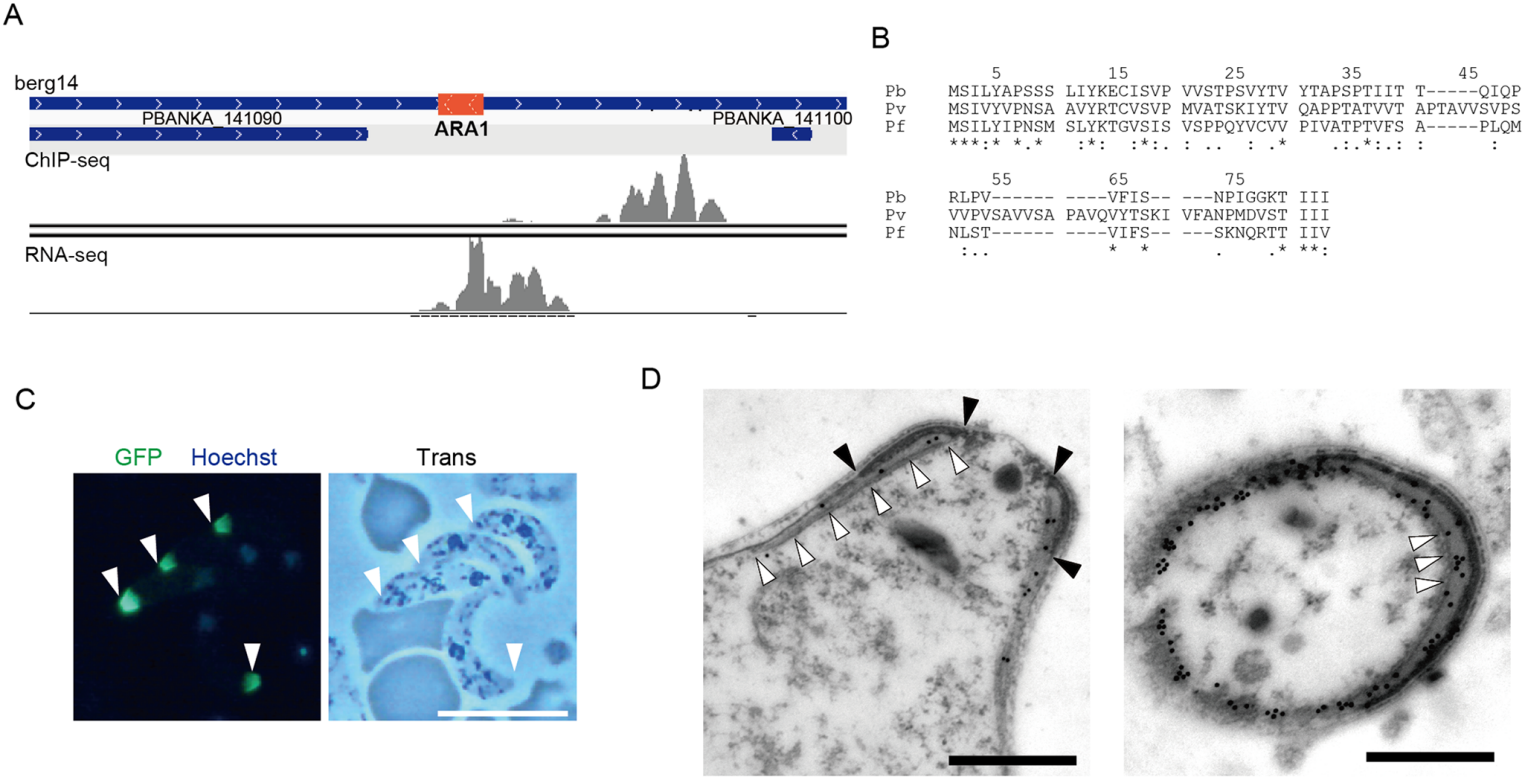
Identification of a missing gene in the *P*. *berghei* genome. A. Mapped view of the ChIP-seq and RNA-seq reads of the identified gene in ookinetes. The RNA-seq reads were located in the intergenic region between the 3′-portions of neighboring genes (PBANKA 141090 and PBANKA 141100). The identified ORF is indicated by a red rectangle. B. The amino acid sequences encoded by the corresponding ORFs were aligned in different *Plasmodium* species. The alignment was performed using ClustalW2.1 (http://www.ebi.ac.uk/Tools/msa/clustalw2). Pb, *P*. *berghei*; Pv. *P*. *vivax*; Pf, *P*. *falciparum*. C. A fluorescence image of *P*. *berghei* ookinetes expressing the GFP-tagged protein. Ookietes were cultured *in vitro* and observed by fluorescence microscopy at 24 h after fertilization. Apical ends of ookinetes are indicated by arrowheads. Left, merged image of GFP and nuclear staining with Hoechst 33342. Right, optical transmission image. Scale bar, 10 μm. D. Immunoelectron microscopy image of an ookinete expressing the putative protein. Immunoelectron microscopy was performed with anti-GFP antibodies. Left, sagittal section. Colloidal gold particles (15 nm) are located on the apical polar ring, a low-electron density area between the collar (edges of the color are indicated by closed arrowheads) and the striated structure of microtubules (indicated by open arrowheads. Right, cross-section image. Colloidal gold particles (15 nm) are located on the apical polar ring, a low-electron density area between the collar (high-electron density area), and microtubules (cross-sections of microtubules are indicated by arrowheads). The colloidal gold particles are also located on the fibrous tissue observed in the lower-left part of the section, which could be the microtubules adhering to the apical ring and the cytoskeletal fibers surrounding them. Scale bars, 0.5 μm.

The target prediction described above was based on the genome annotation in PlasmoDB. However, this annotation is still underway, and some small-sized genes may have been missed. Therefore, we used our data to look for target genes still missing in the *P*. *berghei* genome. We selected AP2-O peaks present in the intergenic regions that had no corresponding targets and then manually looked for open reading frames (ORFs) in the nearby regions using the BLAST program and the RNA-seq data. Using this screening technique, an ORF was identified in the region flanked by the 3′-portions of two genes (PBANKA_141090 and PBANKA_141100, respectively) (Fig 2A). It encoded a small putative protein comprising 60 amino acids that is conserved in *Plasmodium* spp. (Fig 2B).

To examine whether this hypothetical ORF was translated into a protein, it was expressed as a GFP-tagged protein under control of the original promoter, using the centromere plasmid pCen-GFP [8]. Expression of the tagged protein was found at the apical tip of the ookinetes (Fig 2C). Immunoelectron microscopy showed that the GFP-tagged protein was localized to the apical polar ring, corresponding to the low electron density structure beneath the collar (Fig 2D, left). Transverse section images demonstrated that this protein was also distributed at the boundaries between subpellicular microtubules and the apical ring, suggesting that the protein plays a role connecting the above two structures (Fig 2D, right). This is the first *Plasmodium* protein reported to localize to the apical polar ring, and we designated this protein apical ring associated protein 1 (ARA1). No ARA1 homologs have been found in organisms other than *Plasmodium* parasites.

### AP2-O induces genes involved in ookinete morphogenesis and gliding motility

**Fig 3:**
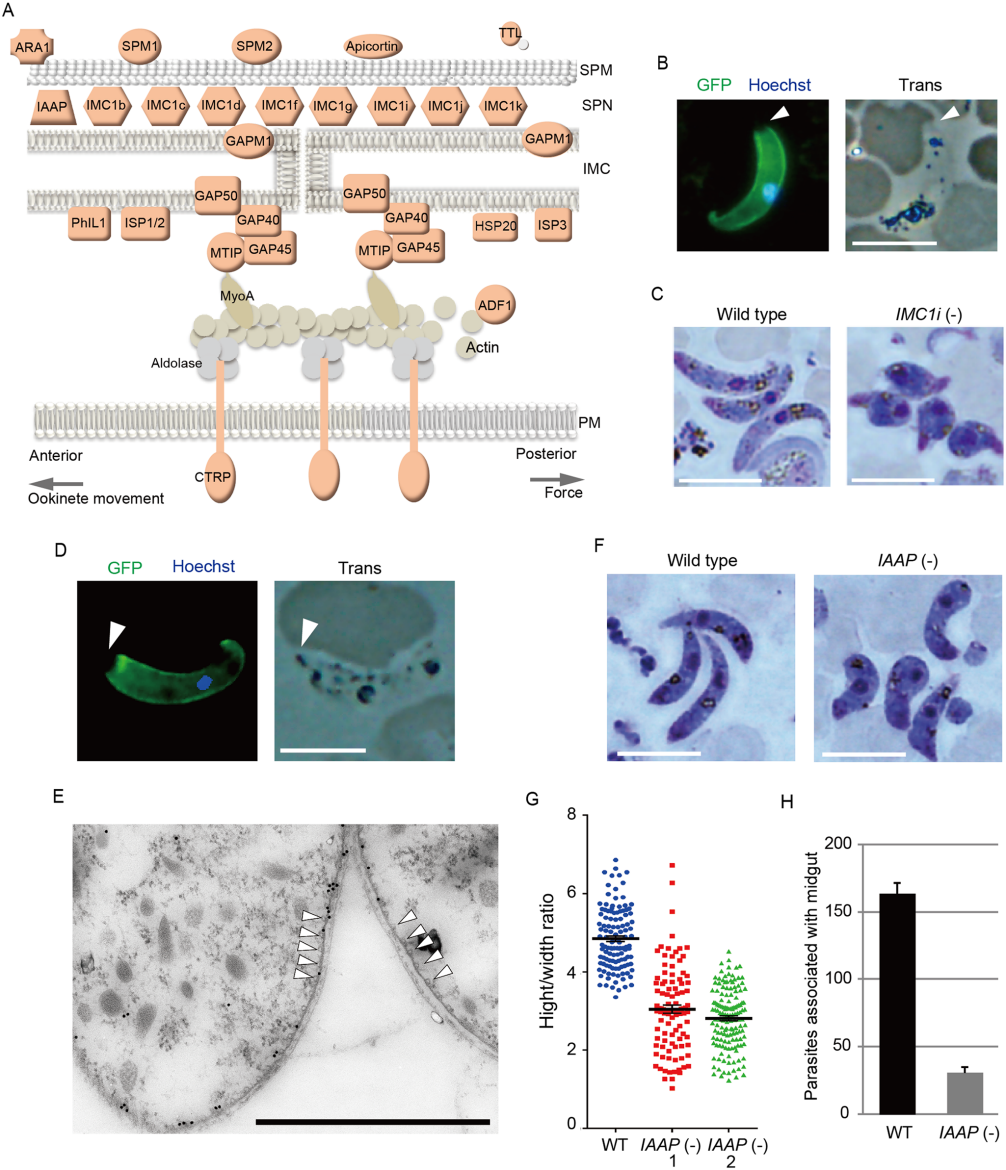
Genes encoding pellicular proteins are AP2-O targets. A. The pellicle structure and its components are illustrated. Targets are highlighted in red. In addition to the proteins mentioned in the text, targets contained several genes encoding putative pellicular/IMC proteins, viz. apicortin, tubulin-tyrosine ligase (TTL), and small heat shock-related protein 20 (HSP20). Apicortin is involved in the stabilization of microtubules [59]. TTL, an enzyme adding a tyrosine to the carboxyl end of tubulin, marks the plus ends of growing microtubules and regulates microtubule growth at this site [60]. HSP20 is involved in ookinete motility [51,61]. MyoA, myosin A; ADF1, actin-depolymerizing factor 1; SPM, subpellicular microtubule. B. IMC1i was expressed as a GFP-tagged protein using a *P*. *berghei* centromere plasmid. Ookinetes were cultured *in vitro* and observed by fluorescence microscopy at 24 h after fertilization. The apical end of the ookinete is indicated by an arrowhead. Left, merged image of GFP and nuclear staining with Hoechst 33342. Right, optical transmission image. Scale bar, 10 μm. C. Giemsa-stained image of wild-type (left) and *IMC1i*(-) (right) ookinetes, 24 h after fertilization. Scale bar, 10 μm. D. Fluorescence microscopy image of ookinetes expressing GFP-tagged PUA26. Left, merged image of GFP and nuclear staining with Hoechst 33342. Right, optical transmission image. Scale bar, 10 μm. E. Immunoelectron microscopy image. Cross-sections of two ookinetes expressing GFP-tagged PUA26 are shown. Immunoelectron microscopy was performed with anti-GFP antibodies. Colloidal gold particles (15 nm) are mainly localized at the IMC, which is the layer of high-electron density beneath the plasma membrane. Subpellicular microtubules are indicated by arrowheads. Scale bar, 1 μm. F. Giemsa-stained image of wild-type (left) and *PUA26*(-) (right) ookinetes at 24 h after fertilization. Scale bar, 10 μm. G. Height—width ratios were compared between wild-type and *PUA26*(-) ookinetes. Ookinetes were cultured for 24 h and stained with Giemsa. Micrographs were obtained with an Olympus BX60 fluorescence microscope. Height—width ratios of ookinetes were measured with the AquaCosmos software (Hamamatsu Photonic System). In total, 100 ookinetes were analyzed in each parasite population. Bars represent mean ± SE. H. The numbers of parasites associated with the midgut were compared between wild-type and *PUA26*(-) parasites at 24 h after an infective blood meal by mosquitoes. Data are the mean ± SE of three independent experiments using 20 mosquitoes each. Only parasites on a single side of the midgut were counted.

The pellicle is a thin structure constituted from the plasma membrane and the closely apposed inner membrane complex (IMC). The IMC is a complex of the membranous structure (IMC membrane) and the subpellicular network (SPN) of cytoskeletal proteins, such as the alveolins/IMC1 proteins. The pellicle underlies the entire parasite plasma membrane, except for the apical portion, and is supported by a row of subpellicular microtubules that originate from the apical ring. The pellicle space contains an actin-myosin motor that generates the driving force for gliding motility. Therefore, this structure is essential for the motile stages of these parasites. Pellicular/IMC proteins have been studied intensively in *Toxoplasma gondii* tachyzoites [20,21], and several pellicle-associated proteins have been reported. Most of these genes have orthologs in the *Plasmodium* genome. However, none of these genes have been identified as AP2-O target genes [7]. In the present study, 22 genes encoding pellicullar/subpellicular proteins were identified as AP2-O target genes (S7 Table in S1 File). The constellation of these target gene products in the pellicle and subpellicular structure is illustrated in Fig 3A.

The I*MC1/alveolin*-like genes constitute the largest group of paralogous genes in this category. Eight IMC1 genes (*IMC1a—IMC1h*) have been identified in the *P*. *berghei* genome [22], and there are five additional members of the alveolin family (tentatively named *IMC1i—IMC1m*) in it (S2 Fig and S7 Table in S1 File). Of these 13 *Plasmodium IMC1*-like genes, eight were identified as AP2-O targets (Fig 3A and S2 Fig). This result indicates that the SPN of this motile stage is composed of a stage-specific cocktail of IMC1 proteins. We generated parasites expressing *IMC1i* as a C-terminal GFP fusion protein to examine if newly identified *IMC1*-like genes actually encode IMC proteins (Fig 3B and S3 Fig). Signals were observed along the surface, but not at the apical end of the mature ookinete. This distribution pattern is characteristic of SPN proteins. We further disrupted the IMC1i gene and investigated the resulting phenotype (S3 Fig). The mutants developed into ookinetes with normal conversion rates (S9 Table in S2 File); however, nearly all of them (99.8%, n = 520) displayed abnormal morphologies (Fig 3C). To exclude the possibility that unfertilized gametocytes were mistakenly counted as immature ookinete, immunofluorescent staining with antibody against circumsporozoite- and TRAP-related protein (CTRP) was performed. Number of ookinetes per microscopic field observed by the immunofluorescent staining [14.7 ± 12.5 (mean ± SE; n = 10)] was essentially same as that observed by Giemsa staining [15.0 ± 11.9 (mean ± SE; n = 10)], which confirmed that round ookinetes-like parasites observed by Giemsa staining were genuine ookinetes. The morphologies were similar to those of ookinetes depleted of *IMC1b*, another ookinete protein in this family [23], and to those of *AP2-O*-depleted ookinetes [7]. They nearly lost their ability to infect mosquitoes (S10 Table in S2 File). These results suggest that the reduced production of these putative cytoskeletal proteins may have caused the abnormal morphology seen in *AP2-O*-depleted ookinetes.

Along with these IMC1 genes, the list of targets included orthologs of the *T*. *gondii* pellicullar/IMC proteins: *P*. *berghei* PhIL1 (*photo*sensitized INA-labeled protein 1), IMC sub-compartment protein 1 (ISP1), ISP3, subpellicular microtubule-associated protein 1 (SPM1), and SPM2 [20,24–26] (Fig 3A). PhIL1 and ISP1 are localized mainly with IMC and the apical cap of *T*. *gondii* tachyzoites, which is an apical structure linked to the IMC membrane that covers the parasite’s apical protrusion. We investigated localization of *P*. *berghei* PhIL1 by generating parasites expressing a GFP-tagged protein. The tagged protein spread along the cell surface of mature ookinetes, but still localized predominantly to the apical protrusion (S4 Fig). This distribution pattern was similar to the pattern reported for *T*. *gondii* PhIL1 [20,21], suggesting that ookinetes have the apical structure corresponding to the apical cap of *T*. *gondii* tachyzoites.

The pellicle also contains the glideosome, a complex of motor proteins (Fig 3A). The glideosome is linked to the cytoplasmic domains of adhesins integrated into the plasma membrane, facilitating the gliding motility of apicomplexan parasites. According to *T*. *gondii* studies, the glideosome is composed of actin filaments, myosin A, the myosin A tail domain-interacting protein, aldolase, glideosome-associated proteins (GAPs) [27], and GAPs with multiple membrane spans [28]. All of the corresponding genes have orthologs in *Plasmodium* parasites, and the majority of them, excluding actin, myosin A, and aldolase, are AP2-O targets (The GAP 50 gene shows a ChIP peak in the 1.4-kbp upstream). Taken together, these results suggest that AP2-O is involved in morphogenesis and motility of ookinetes.

### Exploring novel genes encoding ookinete pellicle proteins

The pellicle proteins described above are linage-specific; they have homologs solely in apicomplexan parasites or alveolates (alveolates are higher-order groups of apicomplexan parasites). Therefore, we explored genes encoding novel pellicle proteins among the target genes that have not been functionally annotated, but which are uniquely conserved in organisms of this lineage. The list of target genes contained 36 genes satisfying these criteria. These genes were tentatively designated *PUA* (protein unique to apicomplexan parasites) *1–36* (S7 Table in S1 File). We expressed three small *PUA* genes (*PUA17*, *PUA19*, and *PUA26*) as GFP-tagged proteins using pCen-GFP and examined their subcellular localization (Fig 3D and S5 Fig). Among these proteins, GFP-tagged PUA26 displayed a distribution pattern characteristic of SPN proteins (localization at the parasite surface, except at the apical end, as shown in Fig 3D). Therefore, we further investigated its localization and function using immunoelectron microscopy and gene targeting (S3 Fig), respectively. By immunoelectron microscopy gold particles were localized predominantly along the parasite surface, except at the apical region (S6 Fig). Under higher magnification, it was evident that the particles were localized not to the plasma membrane, but on the electron-dense structure beneath it, indicating that PUA26 is located on IMC (Fig 3E). Targeting this gene did not affect ookinete conversion rates (S9 Table in S2 File); however, the morphologies of generated ookinetes appeared somewhat laterally longer than those of the wild-type parasites (Fig 3F). Calculation of the height—width ratios of these ookinetes confirmed that they had abnormal morphologies (Fig 3G). The number of oocysts observed in the midgut was also significantly reduced in these parasites (Table 3). We prepared GFP-expressing parasites from these disruptants and investigated the number of ookinetes that successfully reached the midgut lamina at 24 h after an infective blood meal by mosquitoes. Ookinetes observed in the midgut were significantly decreased in the disruptants (Fig 3H), while the majority of them (84.5 ± 5.4%) began to transform into spherical early oocysts, as observed in wild-type parasites (75.5 ± 5.4%). These results suggest that the disruption impaired ookinete cytoskeletal structure and reduced ookinete motility, as reported for other IMC proteins [29]. *PUA26* encodes a 96-amino-acid protein that possesses no known functional motifs. Orthologs of this gene exist in *T*. *gondii* (TGME49_014220), *Neospora caninum*(XP_003884749), and *Eimeria* spp (CDJ60835). We designated this protein as IMC-associated apicomplexan protein (IAAP).

**Table 3:** Oocyst formation in mutant parasites.

Parasite population	Number of oocysts per mosquito (mean±SE)	Number of oocyst sporozoites per mosquito
Wild-type 1	134.4±27.0	27,768
Wild-type 2	106.6±27.6	11,700
Wild-type 3	98.0±25.7	78,068
Wild-type 4	202.4±42.4	43,134
Wild-type 5	150.9±30.1	62,634
*IAAP*(–) 1	19.0±3.0	4,674
*IAAP*(–) 1	14.4±1.9	4,275
*IAAP*(–) 1	11.2±2.3	1,596
*IAAP*(–) 2	17.8±5.3	7633
*IAAP*(–) 2	17.1±2.7	5,061
*IAAP*(–) 2	11.9±3.4	–
*PPLP4*(–) 1	0±0	–
*PPLP4*(–) 1	0±0	–
*PPLP4*(–) 1	0±0	–
*PPLP4*(–) 2	0±0	–
*PPLP4*(–) 2	0±0	–
*PPLP4*(–) 2	0±0	–
*POS8*(–) 1	1.3±0.42	36
*POS8*(–) 1	7.1±2.0	101
*POS8*(–) 1	7.0±2.4	–
*POS8*(–) 1	1.4±0.57	18
*POS8*(–) 2	5.7±1.3	–
*POS8*(–) 2	1.6±0.49	–
*CYC3*(–) 1	43.2±8.0	1,565
*CYC3*(–) 1	10.1±3.8	1,614
*CYC3*(–) 1	21.2±6.2	–
*CYC3*(–) 2	2.2±1.0	152
*CYC3*(–) 2	15.6±4.0	3,268
*CYC3*(–) 2	25.2±5.8	4,408

Two independently prepared mutants were used for assessing the phenotype in each gene. The number of oocysts was counted 14 days after an infective blood meal by mosquitoes. The value is an average from 25 mosquitoes. SE, standard error.

### Putative microneme proteins among the targets

**Fig 4:**
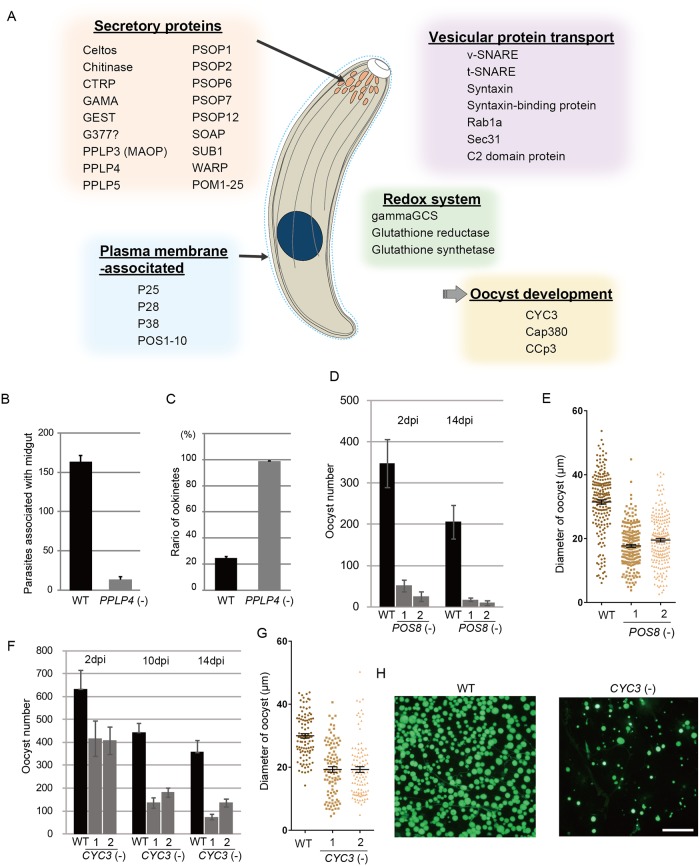
Target genes required for midgut invasion and oocyst formation. A. Overview of the target genes involved in midgut invasion and oocyst formation. B. The numbers of parasites associated with the midgut were compared between wild-type and *PPLP4*(-) parasites at 24 h after an infective blood meal by mosquitoes, as in Fig 3G. Data are the mean ± SE of three independent experiments using 20 mosquitoes each. C. Ratios of okinetes to total parasites associated with midguts at 24 h after an infective blood meal by mosquitoes. Only ookinetes of fully elongated shape were judged as ookinetes. Data are the mean ± SE of three independent experiments using 20 mosquitoes each. D. Oocysts were counted 2 and 14 dpi. Three independent experiments were performed with each clone. Data are represented as mean ± SE. E. Dot plots of diameters of oocysts at 14 dpi. In total, 200 oocysts were used for measurements in each experiment. Bars represent the mean ± SE of diameters. Mosquitoes were fed on mice infected with *POS8*(-) parasites constitutively expressing GFP. F. The oocysts in the midgut were counted at different time points (2, 10, and 14 dpi), using the same GFP-expressing parasites as in H. Three independent experiments were performed with each clone. Data are represented as mean ± SE. G. Dot plots of diameters of oocysts at 14 dpi. In total, 200 oocysts were used for measurements in each experiment. Bars represent the mean ± SE of diameters. Mosquitoes were fed on mice infected with *CYC3*(-) parasites constitutively expressing GFP. H. Fluorescence microscopy image of a mosquito midgut infected with wild-type (left) and *CYC3*(-) (right) parasites expressing GFP at 14 dpi. Scale bar, 300 μm.

Secreted proteins constituted the largest group among the targets; in total, 43 genes encoded a putative secreted protein (S7 Table in S1 File). The majority of them could be microneme proteins, as ookinetes have no secretory organelles for intracellular parasitic infection. The list of targets included all seven proteins reported to be localized to the ookinete microneme: chitinase, CTRP, secreted ookinete adhesive protein, von Willebrand factor A-domain-related protein, cell traversal protein for ookinetes and sporozoites, GPI-anchored micronemal antigen (GAMA), and MAOP (PPLP3), and the seven putative microneme proteins detected during the ookinete proteomic studies: PPLP4, PPLP5, PSOP1, PSOP2, PSOP6, PSOP7, and PSOP12 [19,30–37] (Fig 4A and S7 Table in S1 File). These results indicate that microneme proteins are induced as a set by AP2-O in developing ookinetes. Another 25 unannotated genes in this category were tentatively designated here as *POM* (putative ookinete microneme proteins) *1–25* (S7 Table in S1 File).

In addition to these genes, the list of predicted targets included two genes encoding secreted proteins of the osmiophilic body, which is the secretory organelle of gametocytes. We expressed one of the genes, viz. *gamete egress and sporozoite traversal protein* (*GEST*), as a GFP-tagged protein and demonstrated that it is expressed in the ookinete as a microneme protein (S7 Fig). As the osmiophilic body is involved in egress of the gametocyte from the host erythrocyte, its expression during motile stages suggests that parasites employ common mechanisms for egression and invasion into host cells. This similarity between the two organelles was also suggested from the finding that perforin-like proteins are used for egression by gametocytes [38] and for invasion by ookinetes and sporozoites, respectively [35,39].

We generated disruptants in five target genes belonging to this category whose roles in ookinetes remain to be elucidated: CS domain protein, *PPLP4*, *POM2*, *POM7*, and *POM16*. They were all successfully disrupted, but a clear reduction in oocysts was observed only with *PPLP4* (Table 3 and S11 Table in S2 File). *PPLP4* encodes one of the five PPLP genes (*PPLP1*–*PPLP5*) identified in the *P*. *berghei* genome that contains a membrane attack complex/perforin (MACPF) domain. Targeting *PPLP4* did not affect ookinete conversion rates (S9 Table in S2 File) and morphologies of ookinetes but resulted in the complete loss of infectivity to mosquitoes (Table 3). We generated parasites that constitutively expressed GFP from these disruptant populations and assessed midgut invasion by ookinetes *in vivo* at 24 h after an infective blood meal by mosquitoes (Fig 4B and 4C). A small number of ookinetes were observed in the midgut (Fig 4B). However, almost all these ookinetes still displayed elongated shapes (99.5 ± 1.4%), whereas in wild-type parasites, the majority of ookinetes associated with the midgut had already started to transform into early oocysts (Fig 4C). This means that the ookinetes associated with the midguts observed in the parasites may not have arrived at the basal lamina but may have rather attached to the apical side of the epithelial cells, as we reported in *PPLP3*/*MAOP* disruptants [35]. Three PPLPs, *PPLP3*–*PPLP5*, were detected in ookinete micronemes during a proteomic study [19], and *PPLP3* and *PPLP5* are essential for ookinete infection of the midgut [35,40]. The present results demonstrated that all three ookinete PPLP genes were AP2-O targets and are essential for infection of the midgut. This is in clear contrast with the redundancy of other target genes in this category, raising the possibility that these PPLPs assemble into a protein complex and form MAC on the plasma membrane of a target cell, as do human complement proteins C6–C9, which all contain a MACPF domain [41].

In addition to secretory proteins, the list of targets contained genes involved in vesicular protein transport (Fig 4A and S7 Table in S1 File). Considering that the predominant secretory proteins in this stage are all microneme proteins, these genes could be involved in transport and secretion of microneme proteins. In particular, several proteins, possibly constituting the soluble N-ethylmaleimide-sensitive factor activating protein receptor (SNARE) machinery, were included in the list of targets. These included vesicle-associated SNARE protein, target-associated SNARE protein, syntaxin, syntaxin-binding protein, mammalian uncoordinated protein 18-related protein, and a C2-domain protein with a transmembrane region (S7 Table in S1 File). The functions of these genes during this stage remain elusive, but our data could serve as a resource for future investigations into the mechanism of ookinete microneme secretion.

### Putative plasma membrane-associated proteins on the list of targets

We classified genes encoding proteins with an N-terminal signal sequence and a structure for anchoring to the plasma membrane, such as the membrane-integrated domain and the GPI modification site, as putative plasma membrane-associated proteins. This group could include proteins that are targeted to the parasite surface after being secreted from micronemes, such as CTRP and GAMA, as well as proteins directly targeted to the cell surface. Genes in this group are important for ookinete biology because their products could be involved in parasite interactions with the midgut epithelium, and are still largely unknown. The list of targets contained 14 of these genes, including two genes that have been reported to be expressed during this stage: *P25* and *P28* (Fig 4A and S7 Table in S1 File, respectively). The *MSP10* gene *may* have been incorrectly predicted as a target, mostly because the AP2-O binding site is very close to the start codon (< 50-bp) and probably exists in the 5′-UTR. This finding suggests that the region close to the start codon must be excluded from target prediction. Ten other unannotated genes in this category were tentatively designated as putative ookinete surface-associated protein (*POS*) *1–10* (S7 Table in S1 File).

We performed targeting experiments with three genes of unknown function in this category (*POS7–9*) (S3 Fig), and we investigated their involvement in midgut infection. During the targeting experiments, a significant decrease in the number of oocysts was observed only for the *POS8* disruptant (Table 3 and S12 Table in S2 File). We further analyzed the phenotype of this disruptant using independently prepared disruptant populations. In these disruptants, the number of oocysts decreased more than 20-fold (Table 3). The size of parasite oocysts was clearly smaller than that of the wild-type parasite oocysts [as observed by phase contrast microscopy at 14 days post-infection (dpi)]. Therefore, to identify the step in which they decreased in number, we generated parasites that constitutively expressed GFP from these disruptant populations and counted the oocysts shortly after ookinete invasion of the midgut at 2 dpi (Fig 4D). The number of oocysts was already approximately 10-fold smaller than that of wild-type parasites. We further measured oocyst diameters by epifluorescence microscopy at 14 dpi. As shown in Fig 4E, the average oocyst diameter was approximately 60% of that of the wild-type oocysts, suggesting that oocyst development was impaired by this disruption. This phenotype indicates that *POS8* plays a critical role in ookinete-mediated midgut invasion as well as in subsequent oocyst development. We speculate that this gene product might participate in oocyst development by establishing the foothold necessary for subsequent oocyst development. To determine the localization of this protein at the surface of the ookinetes and to explain the phenotype of the disruptants by *in vivo* observation, we transfected wild-type parasites with a centromere plasmid containing a construct for expressing *POS8* as a GFP-fused protein. However, we could not detect GFP signals in the ookinetes of these parasites. At present, we speculate that the fusion of GFP to the putative membrane-associated region of the protein may affect its targeting to the plasma membrane. Further study is required to explain why the abnormal phenotypes were observed over the two stages.

### The glutathione redox system is activated by AP2-O for survival in mosquitoes

Reactive oxygen species (ROS) are produced in mosquitoes after a blood meal and in response to penetration of the midgut epithelium by ookinetes. Epithelial cells play a critical role eliminating parasites from the midgut [42,43]. The parasite reduction-oxidation (redox) system is essential for parasites to combat these oxidative stressors and is therefore critically important for their midgut survival. Parasites have two redox systems, a thioredoxin system and a glutathione system [44]. Of these two systems, the glutathione system plays a central role in midgut parasite infection and oocyst development [45,46]. This system is maintained by three genes that produce gamma-glutamylcysteine synthetase (gammaGCS), glutathione synthetase, and glutathione reductase. Glutathione is synthesized *de novo* from glutamine and cysteine by gammaGCS and glutathione synthetase. Glutathione reductase reduces oxidized glutathione to glutathione. A ChIP-seq analysis showed that all of these genes are AP2-O targets (Fig 4A and S7 Table in S1 File), indicating that these genes are “programmed” to be induced during this stage prior to midgut invasion, thereby protecting ookinetes from the mosquito immune system during midgut penetration. This is in clear contrast to the mechanisms for sensing and responding to oxidative stressors observed in other eukaryotes. The glutathione system is also important for oocyst development. Considering that transcripts of these genes are abundant in the ookinete stage, it is possible that transcripts of these genes in ookinetes are prepared in part to protect early oocysts from ROS produced in the midgut.

### AP2-O induces the genes required for oocyst formation

After arrival at the basal side of the midgut, ookinetes lose their elongated shape and transform into spherical oocysts. The list of AP2-O targets contained the Cap380 gene, which encodes the oocyst major capsule protein [47]. RNA-seq analysis showed that the transcripts of this gene are abundant in ookinetes (S13 Table in S2 File), and DNA microarray analysis showed that its expression decreased more than 10-fold in *AP2-O*(-) parasites (Table 1). This finding suggests that the AP2-O target genes could include genes for oocyst development and that expression of a portion of target genes is post-transcriptionally regulated in ookinetes for the subsequent oocyst stage as reported in other stages, such as the female gametocyte stage or the sporozoite stage [15,48]. The list of targets contained a number of gene products with RNA-interacting motifs, such as the zinc finger motif and RNA recognition motifs (Fig 1G and S7 Table in S1 File). They could be involved in post-transcriptional regulation of such genes and contribute to the progression of ookinetes into oocysts.

We found another target gene with an important role in oocyst development. This gene (PBANKA_123320), which encodes a cyclin-like protein, was tentatively named *CYC3*, to be consistent with the annotation of its ortholog in *P*. *falciparum* (PlasmoDB). Among the five genes annotated as cyclins in the *P*. *berghei* genome, *CYC3* is most abundantly expressed in ookinetes (S13 Table in S2 File). Parasites in which this gene was disrupted (S3 Fig) showed a normal ookinete conversion rate (S9 Table in S2 File) and formed morphologically normal ookinetes. However, the numbers of oocysts and oocyst sporozoites at 14 dpi were several-fold lower than those present in the wild-type (Table 3). Moreover, oocysts observed with phase-contrast microscopy were smaller than corresponding wild-type oocysts. Following this, we prepared disruptants constitutively expressing GFP from the same set of parasites and conducted a time-course study of the number of oocysts. The oocyst number of the disruptants was approximately 75% of that of wild-type parasites at 2 dpi and decreased to 20%–30% of that of the wild-type parasites at 14 dpi (Fig 4F). A high proportion of small-size oocysts, which were too small to be detected by phase-contrast microscopy, were observed by epifluorescence microscopy at 14 dpi (Fig 4G and 4H). The average diameter of the oocysts at this time point was approximately 65% of that of wild-type oocysts (Fig 4G). By nuclear staining with cell permeable Hoechst 33342 at 4 dpi, nuclei of most disruptants were not detected (27 of 30 oocysts), suggesting that nuclear division scarcely proceeded in them. To exclude the possibility that uncompleted meiosis during zygote to ookinetes transition affected the subsequent oocyst development [49,50], we measured the content of nuclear DNA in mature ookinetes by staining with Hoechst 33342. No differences in DNA contents were observed between wild-type ookinetes and disruptants (S8 Fig), indicating that meiosis was normally completed in the disruptants. Collectively, these results strongly suggest that CYC3 controls cell cycle progression in early oocysts.

## Discussion

We explored the AP2-O targets with ChIP-seq analyses, identified approximately 1,100 binding sites on the genome, and predicted over 500 genes as targets. The list of targets included a set of genes necessary for pellicle formation, which finally explained why ookinetes with disrupted AP2-O display abnormal morphologies. The targets also included a series of genes necessary for midgut infection by the parasite. The targets comprised genes encoding proteins for gliding motility, microneme proteins, and surface proteins; genes necessary for the redox system; and genes for oocyst development. Further, although not mentioned specifically, the list of targets included genes encoding protein kinases (Fig 1G and S7 Table in S1 File), three of which, viz. CDPK1, CDPK3, and PBANKA_146050, are essential for midgut infection of ookinetes [51–53]. These results show that a single transcription factor is involved in all processes of ookinete-mediated midgut invasion and that transcriptional regulation during this stage is centralized to this transcription factor.

This transcriptional regulation in ookinetes is in clear contrast to that reported in model eukaryotes, in which a network system composed of a number of transcription factors regulates gene expression. A transcription factor usually regulates functionally related genes in budding yeast [6], and a bundle of these modules constitutes a transcriptional regulatory system [54]. Therefore, different biological pathways can be activated independently, enabling the organism to create different gene expression patterns according to environmental conditions. In contrast, transcriptional regulation in ookinetes definitely lacks the flexibility necessary for adapting to environmental change. In this stage, parasites may not be able to control a group of genes separately from other target genes. Hundreds of target genes should be induced in a set according to the program defined in advance for this stage. This observation suggests that the parasites can survive only under limited environmental conditions.

Obviously, stable and predictable environments are prerequisite for this inflexible system, and their parasitic lifestyle seems to ensure them such environments. It would be reasonable to speculate that parasitism relieved them from the necessity to respond to environmental change and allowed them to reduce the number of regulatory genes including transcription factors. If the ookinete regulatory system is an evolutionary consequence of the parasitic lifestyle, it would not be surprising if these parasites adopt similar regulation systems in other lifecycle stages.

Major families of transcription factors different from the AP2-family have not been demonstrated, supporting the assumption that the AP2-family is the sole sequence-specific transcription factor family of this parasite and suggests that the total number of transcription factors in these parasites is about 30, which is an order smaller than those in ordinary eukaryotes, because transcription factors usually constitute 5–10% of the eukaryote genome and these parasites possess >5000 genes in their genome. This paucity of transcription factors and their complicated lifecycle appear to oppose each other and suggest that these parasites have a unique gene regulatory system. In this study, we provide the first information regarding the parasite transcriptional regulatory system and revealed that a single master transcription factor directly regulates the broad range of targets in ookinetes. This finding suggests one simple gene regulatory model in these parasites as follows: Each lifecycle stage possesses a stage-specific master transcription factor. That factor directly induces a number of target genes during the stage, creating a stage-specific gene expression repertoire. This simple model could explain the paucity of transcription factors in these parasites [2,55]. To examine if this model could be extended to other stages, we are now performing ChIP-seq analyses of AP2 transcription factors in several lifecycle stages including the proliferation and motility stages.

AP2-O was first observed in the nucleus of the developing zygote/ookinete approximately 8 h after fertilization [7]. This observation suggests that AP2-O would not be involved in transcriptional regulation in the early development of ookinetes. This is also supported by the fact that parasites disrupted with *AP2-O* can develop into retort forms [7]. Therefore, it is possible that other sequence-specific transcription factors are expressed during this period and participate in transcriptional regulation of this stage. However, at present, the most likely regulation involved in this early development would be translational regulation. It has been reported that large amounts of transcripts are stored in female gametocytes for development in this period and that the development of parasites lacking RNA helicase DOZI, the major component of this translation regulation system, is completely halted during the early phase [15]. The expression of AP2-O is also regulated by this regulation system, and as demonstrated by cross-fertilization experiments, its transcripts are mainly derived from the female gametocyte [7]. Therefore, it is possible that gene expression in this period largely depends on transcripts prepared in female gametocytes. On the other hand, RNA-seq analysis in the present study showed that most highly expressed genes in mature ookinetes are targets of AP2-O, strongly suggesting that the development in the later stage would depend on transcriptional regulation by AP2-O. Therefore, it seems that corroboratory regulation by these two gene regulation systems contributes to the transition from gametocytes to ookinetes in the mosquito midgut. Elucidation of gene regulation in this transition stage is a next important theme because it may deepen our understanding of the parasite lifecycle that is constituted from serial stage conversions in host animals.

We demonstrated that target information obtained by ChIP-seq provided an overview of the molecular events proceeding in ookinetes. Based on this target information, we identified novel genes important for midgut infection by this parasite. Importantly, two independent experiments demonstrated consistency of peaks and calculated targets, demonstrating the robustness of this method. This result suggests that analyzing the entire set of target genes of a transcription factor (or targetome analysis) is a powerful way to study the biology of this parasite. Obviously, success in this attempt with ookinetes occurred largely because of the unique transcriptional regulatory features described above. In fact, if ookinete gene expression was regulated by a network of sequence-specific transcription factors, only a partial view of this stage would have been possible. Our results suggest that this method could be a potent omics tool for studying the biology of malarial parasites.

In conclusion, we report the first application of ChIP-seq to genome-wide identification of transcription factor targets in a malaria parasite. We determined the entire set of target genes of a malaria parasite transcription factor and elucidated how the AP2 family transcription factor contributes to formation of the motile stage. In addition, we revealed a unique gene regulatory system that is employed in ookinetes and possibly in other stages of the parasitic lifecycle.

## Materials and Methods

### Ethics statement

This study was carried out in strict accordance with the recommendations in the Guide for the Care and Use of Laboratory Animals of the National Institutes of Health. The protocol was approved by the Committee on the Ethics of Animal Experiments of the Mie University (Permit Number: 23–29). All efforts were made to minimize animal suffering during the course of these studies.

### Parasite preparations

The ANKA strain of *P*. *berghei* was maintained in female BALB/c mice (6–10 weeks old; Japan SLC, Inc., Hamamatsu, Japan). Ookinete culturing was performed as previously described [7]. To examine the number of oocysts, infected mice were subjected to *Anopheles stephensi* mosquitoes. Fully engorged mosquitoes were selected and maintained at 20°C. The number of oocysts and oocyst sporozoites was evaluated 14 days after an infective blood meal.

### ChIP-seq

For the ookinete culture, six mice were pre-treated with phenylhydrazine and then infected with *P*. *berghei* expressing GFP-fused AP2-O. Sulfadiazine was added to their drinking water (10 mg/L) in order to deplete asexual blood-stage parasites. When the exflagellation of the male gametes reached approximately 300 per 10^5^ red blood cells, the blood was harvested and cultured in an ookinete culture medium for 16 h, and then fixed with 1% paraformaldehyde. Erythrocytes in the fixed culture were removed by lysis in 0.84% NH_4_Cl, and the remaining ookinetes were subjected to ChIP. ChIP was performed using the ChIP Assay Kit (Millipore) according to the manufacturer’s protocol. Briefly, samples in the lysis buffer were sonicated with a Bioruptor (Tosho Denki, Yokohama, Japan) until chromatin DNA was fragmented to 300–500-bp in size for sequencing with an Illumina Genome Analyzer and to 150-bp for sequencing with a SOLiD 5500 system (Life Technologies). Immunoprecipitation (IP) was performed with anti-GFP antibodies, and the harvested DNA fragments were subjected to sequencing. Input DNAs were obtained from the chromatin without IP. Anti-GFP antibodies used for ChIP were purchased from Clontech and Abcam.

### Analysis of ChIP-seq data

In experiment 1, sequence data obtained with an Illumina Genome Analyzer were mapped onto the *P*. *berghei* genome sequence (PlasmoDB, version 12.0) using the Bowtie program under conditions allowing one mismatch within 35-bp. In experiment 2, sequence data obtained with a SOLiD 5500 system were mapped onto the *P*. *berghei* genome sequence with a lifescope program equipped with the sequencing system in the default conditions and then filtered under more stringent conditions allowing no mismatches within 60-bp using an in-house program. The mapping data were analyzed with the MACS2 peak-calling algorithm using approximately 0.22 × 10^7^ reads for IP and 0.77 × 10^7^ reads for input control in experiment 1 or 0.22 × 10^7^ reads for IP and 1.12 × 10^7^ reads for input in experiment 2. Conditions for peak calling included FDR < 0.01 and fold enrichments over input control > 5 in both analyses. Genes were identified as AP2-O targets when their 1.2-kbp upstream regions contained the nearest binding motifs from the predicted summits of the ChIP-seq peaks. When the upstream region was less than 1.2-kbp, the entire intergenic region was used for target prediction. The ChIP-seq data have been deposited to Gene Expression Omnibus (GEO) with the accession no. GSE58584. Gene ID and functional annotation were attributed to each gene according to those in PlasmoDB ver.12.0.

To identify the binding sequences of AP2-O, six-base sequences concentrated around the predicted summits were investigated. Fisher’s exact test was performed between the 200-bp regions that have summits in the center and 200-bp regions excised from the genome excluding the former regions, to cover the entire genome sequence. Six-base sequences were ordered according to the calculated p-values, and common sequence motifs were searched among the sequences with the least p-values.

### RNA-seq

Ookinetes were obtained from asexual parasite-depleted infected mouse blood that had been cultured for 24 h in an ookinete culture medium. Total RNA was extracted using the RNeasy mini kit (Qiagen, Hilden, Germany) according to the manufacturer’s protocols. Poly (A)+ RNA was purified using the Oligotex-dT30 mRNA purification kit (Takara Bio, Japan). The harvested RNA was used for sequencing with the SOLiD sequencing system. cDNA libraries for sequencing were prepared according to manufacturer protocols. Reads were mapped on the *P*. *berghei* genome, allowing no mismatches within 60-bp. The total number of reads mapped onto the genome were 15,740,028. The RNA-seq data have been deposited to GEO with the accession no. GSE58584.

### DNA microarray

RNA samples used for the DNA microarray analysis were identical as those used in the previous study (seven biologically independent *AP2-O*(-) and five biologically independent wild-type ookinete samples) [7]. The DNA microarray experiments were performed with the one color method using a custom chip designed on a Agilent platform, as previously described [11]. The data were analyzed by using the GeneSpring software (Agilent Technologies, Santa Clara, CA), and genes whose expression was reduced more than two-fold relative to the wild-type were selected. Genes located at subtelomeric regions were excluded from the analysis. The microarray data have been deposited to GEO with the accession no. GSE58584.

### Gene-targeting experiments

Gene-targeting experiments were carried out essentially using the same procedure as described previously [7]. The genotypes of cloned parasites were checked by PCR. Primers used for the preparation of targeting constructs and for the purpose of genotyping are listed in S14 Table in S2 File. The infectivity of parasites was estimated by examining the oocyst number. When the oocyst number was normal in one disruptant clone, the phenotype was not investigated further. When this number decreased, relative to that of the corresponding wild-type parasites, mutant parasites were obtained by another transfection experiment, and the phenotype and the genotype were subsequently determined.

### Analysis of protein targeting using *P*. *berghei* centromere plasmid

Protein targeting was investigated by expressing each gene as a GFP-tagged protein, using the centromere plasmid pCen-GFP. Target genes with the upstream regulatory region (1.1–1.5-kbp upstream of the first methionine codon) were amplified by PCR using genomic DNA as the template, and then subcloned into the region immediately upstream of the GFP gene of the pCen vector. Merozoites were transfected with these constructs as reported previously [7]. The primers used for the preparation of the pCen constructs are listed in S14 Table in S2 File. The micrographs were obtained with an Olympus BX60 fluorescence microscope (Olympus, Tokyo, Japan) with a DXM1200C digital color camera (Nikon Corporation, Tokyo, Japan).

### Preparation of the centromere plasmid vector with a sulfadiazine-resistant selectable marker

The sulfadiazine-resistant *P*. *falciparum* DHPS (Dihydropteroate synthase) gene (Lys-460 to Glu) was generated by PCR using the primers listed in S14 Table in S2 File, using *P*. *falciparum* 3D7 genomic DNA as a template. The *P*. *berghei* centromere plasmid pCen-GFP-mDHPS was prepared from the *P*. *berghei* heat shock protein promoter, the GFP gene, the *P*. *berghei* heat shock protein 70 termination sequence, the *P*. *berghei* elongation factor 1 alpha promoter, the Sulfadiazine-resistant *P*. *falciparum* DHPS gene, the *P*. *berghei* DHFR-ts (Dihydrofolate reductase-thymidylate synthase) transcription termination sequence, and the plasmid vector pBluescript II SK+ (Agilent Technologies). Pyrimethamine-resistant mutant parasites were transfected with this centromere plasmid, and transfectants were selected in mice by adding sulfadiazine to the drinking water (10 mg/mL). Micrographs were obtained with an Olympus BX60 fluorescence microscope. The oocyst diameter was measured with the AquaCosmos software (Hamamatsu Photonic System).

### Immunoelectron microscopy

Immunoelectron microscopy was performed as described previously [37]. Briefly, ookinetes were fixed in 0.1 M phosphate buffer (pH 7.4) containing 1% paraformaldehyde and 0.1% glutaraldehyde (TAAB), after a 24-hour culture. They were dehydrated in ethanol and embedded in LR Gold resin (London Resin Company, UK). Ultrathin sections were blocked for 30 min in PBS containing 0.01% Tween 20 and 5% non-fat dry milk, incubated with anti-GFP antibodies, and then with goat anti-rabbit IgG conjugated to gold particles of 15 nm diameter (Amersham Pharmacia Biotech) diluted in a blocking buffer. Finally, the sections were fixed with 2.5% glutaraldehyde for 10 min and stained with 2% uranyl acetate and Reynold’s lead citrate. The anti-GFP antibodies used were identical to those used in the ChIP assay.

## Supporting Information

S1 Fig The coding region acts as a promoter for the induction of transcription of some target genes of AP2-O. ChIP-seq peaks of AP2-O and reads of RNA-seq in the ookinete stage were shown in four genes. They have relatively a short upstream intergenic region and their transcripts were induced from the AP2-O binding sites within the coding region of the adjacent gene. Red and blue colors of reads indicate the direction in which they were mapped onto the genome (red: 5′–3′, blue: 3′–5′). Genes are depicted under each panel. Arrows indicate the direction of transcription. Views were generated with the Integrative Genomics Viewer (Robinson *et al*., 2011).
(TIF)

S2 Fig Identification of genes belonging to the alveolin family and the *Plasmodium* genome. Eight *IMC1* genes (*IMC1a—IMC1h*) have been identified so far in the *Plasmodium* genome, and by BLAST search, additional five genes were identified as members of the *IMC1/alveolin* family. They were tentatively named *IMC1i– IMC1m*. Of them, eight genes are AP2-O targets (highlighted in red).
(TIF)

S3 Fig Genotype analyses of genetically modified parasite populations. A. Genotypes of all mutant parasites were checked by PCR, using two primer sets for detecting the wild-type (WT) and the knockout (insertion of knockout construct; KO) parasites, respectively. Primers are listed in S14 Table in S2 File. B. When gene disruption resulted in an abnormal phenotype, another independent mutant parasite population was prepared, and the genotypes were confirmed by Southern blot analysis. Primers used for preparing probes are listed in S14 Table in S2 File.
(TIF)

S4 Fig Localization of PhIL1 in *P*. *berghei* ookinetes. A. Fluorescence microscopy image of ookinetes expressing GFP-tagged PhIL1. Arrows indicates the apical end. Trans, a transmission image. Scale bar, 10 μm. B. Immunoelectron microscopy image of a sagittal section of an ookinete expressing GFP-tagged PhIL1. Colloidal gold particles are mainly localized at the structure of high electron density that is at the apical end adjacent to the IMC and which covers the apical protrusion. The structure seems to be that of the apical cap of ookinetes. Scale bar, 1 μm. C. Immunoelectron microscopy image of a longitudinal section of an ookinete expressing GFP-tagged PhIL1. PhIL1 is also localized at the structure of high electron-density, i.e., IMC (indicated by an open arrow), but not at the plasma membrane (indicated by a closed arrow), in the portion where the plasma membrane was detached from the IMC.
(TIF)

S5 Fig Candidates for pellicular/IMC proteins among the targets. Two genes, viz. PBANKA_083040 (A) and PBANKA_092120 (B) were expressed in *P*. *berghei* ookinetes as GFP-tagged proteins under the control of their original promoters, using pCen-GFP. A. Fluorescence microscopy image of ookinetes expressing GFP-tagged PUA17. The product of *PUA17* (PBANKA_083040) was distributed at the apical end of mature ookinetes and along the ookinete surface. The distribution pattern was similar to that of PhIL1. Thus, it might be located at the apical cap of ookinetes, but this was difficult to determine further by fluorescence microscopy. Scale bar, 10 μm. PUA17 has orthologs only in coccidian parasites such as *Eimeria tenella* and *Toxoplasma gondii*. This gene, designated as G2 (glycine at position 2), is necessary for ookinete motility (Tremp *et al*., 2013). B. Fluorescence microscopy image of ookinetes expressing GFP-tagged PUA19 (PBANKA_092120). Tagged protein showed a trapezoidal appearance similar to that of ARA1, suggesting localization at an apical structure, such as a conoid or an apical ring. Scale bar, 10 μm.
(TIF)

S6 Fig Localization of PUA26 in *P*. *berghei* ookinete. Longitudinal section of an ookinete expressing GFP-tagged PUA26 is shown. Immunoelectron microscopy was performed with anti-GFP antibodies. Colloidal gold particles (15 nm) are mainly localized along the parasite surface except at the apical end. Scale bar, 1 μm.
(TIF)

S7 Fig *GEST* is expressed in ookinetes. *P*. *berghei* parasites expressing GFP-tagged GEST were prepared using pCen-GFP and its expression in ookinetes was investigated. Scale bars, 10 μm. A. Gametocytes in the blood of mice. The tagged protein was observed as particles in the cytoplasm of gametocytes, as previously reported (Talman *et al*., 2011). B. Ookinetes cultured for 24 h after fertilization. GFP-tagged GEST was observed in vesicle-like particles within the apical portion of the cytoplasm, suggesting that it is a microneme protein. C. Ookinetes were cultured for 22 h after fertilization, fixed with acetone for 1 min, and double-stained with mouse anti-GFP antibody [Dylight 488 (green)] and rabbit anti-CTRP antibody [Dylight 549 (red)]. The nucleus was stained with 4',6-Diamidino-2-phenylindole (DAPI).
(TIF)

S8 Fig Comparison of nuclear DNA contents between wild-type and *CYC3*(–) ookinetes. Ookinetes cultured for 24 h were stained with Hoechst 33342. Micrographs were obtained with an Olympus BX60 fluorescence microscope. DNA content of the ookinete nucleus was measured with the AquaCosmos software (Hamamatsu Photonic System). Haploid blood stage parasites were used as controls. In the graph, the values of wild-type ookinetes (tetraploid) were shown as 100%. Values are the mean ± SE of 50 parasites.
(TIF)

S1 File Data of ChIP-seq analyses (S1–8 Tables). (DOCX)

S2 File Phenotype analysis of disruptants (S9–14 Tables). (DOCX)

S1 Data S ChIP-seq bedgraph. (ZIP)
