# Supplementary figures and images for: Genome-Wide Identification of the Target Genes of AP2-O, a Plasmodium AP2-Family Transcription Factor
Source: PLoS Pathog. 2015 May 27;11(5):e1004905. doi: 10.1371/journal.ppat.1004905 (PMC4446032; doi:10.1371/journal.ppat.1004905)

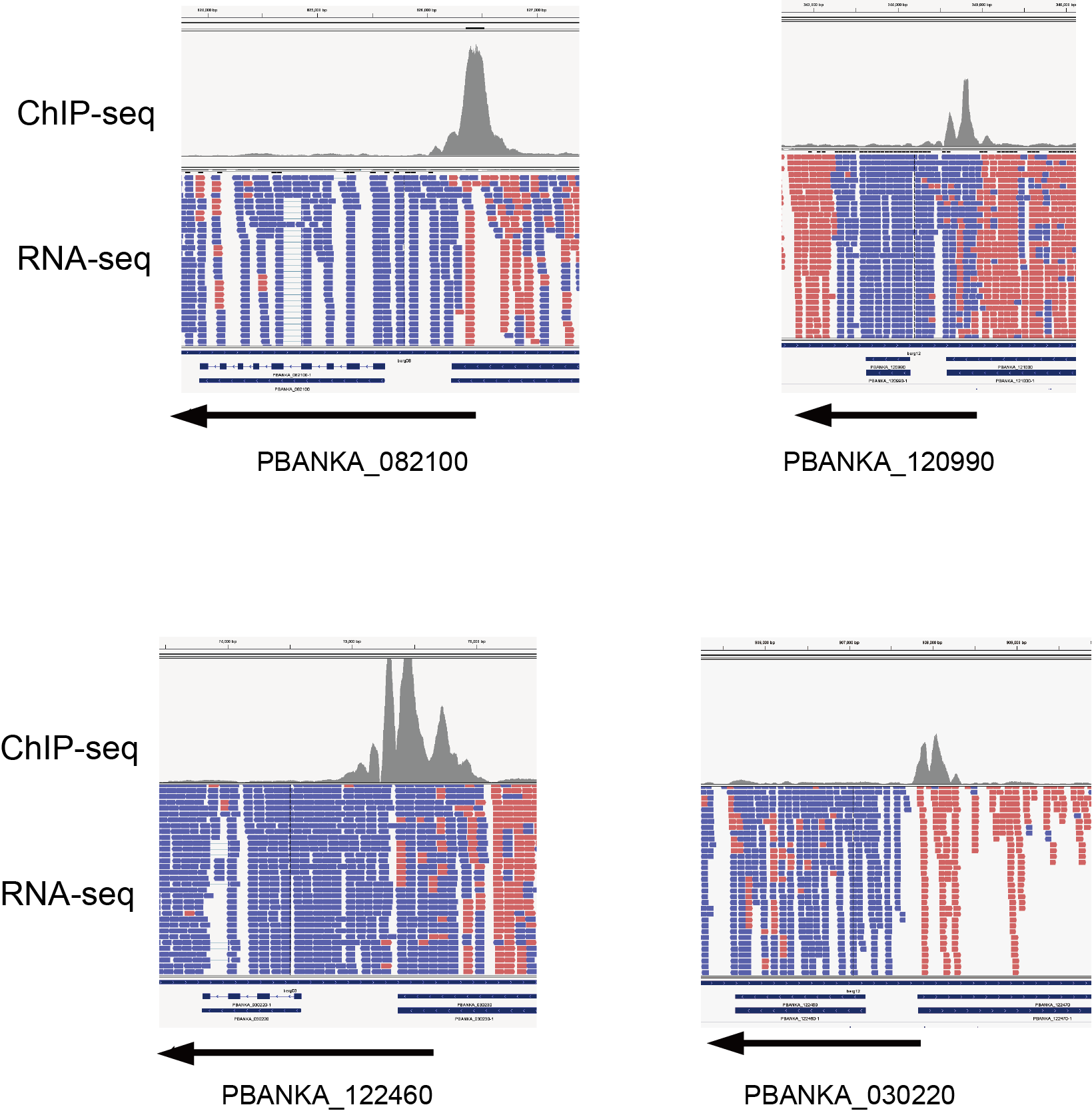

Supplement: S1 Fig — ChIP-seq peaks of AP2-O and reads of RNA-seq in the ookinete stage were shown in four genes. They have relatively a short upstream intergenic region and their transcripts were induced from the AP2-O binding sites within the coding region of the adjacent gene. Red and blue colors of reads indicate the direction in which they were mapped onto the genome (red: 5′–3′, blue: 3′–5′). Genes are depicted under each panel. Arrows indicate the direction of transcription. Views were generated with the Integrative Genomics Viewer (Robinson et al., 2011). (TIF) [file ppat.1004905.s001.tif]

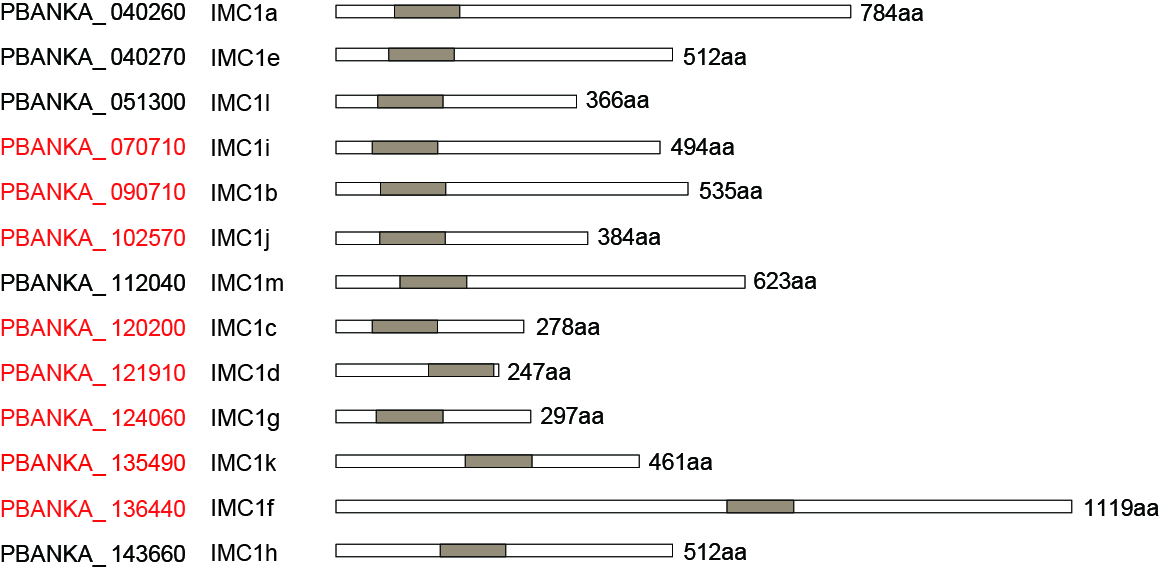

Supplement: S2 Fig — Eight IMC1 genes (IMC1a—IMC1h) have been identified so far in the Plasmodium genome, and by BLAST search, additional five genes were identified as members of the IMC1/alveolin family. They were tentatively named IMC1i– IMC1m. Of them, eight genes are AP2-O targets (highlighted in red). (TIF) [file ppat.1004905.s002.tif]

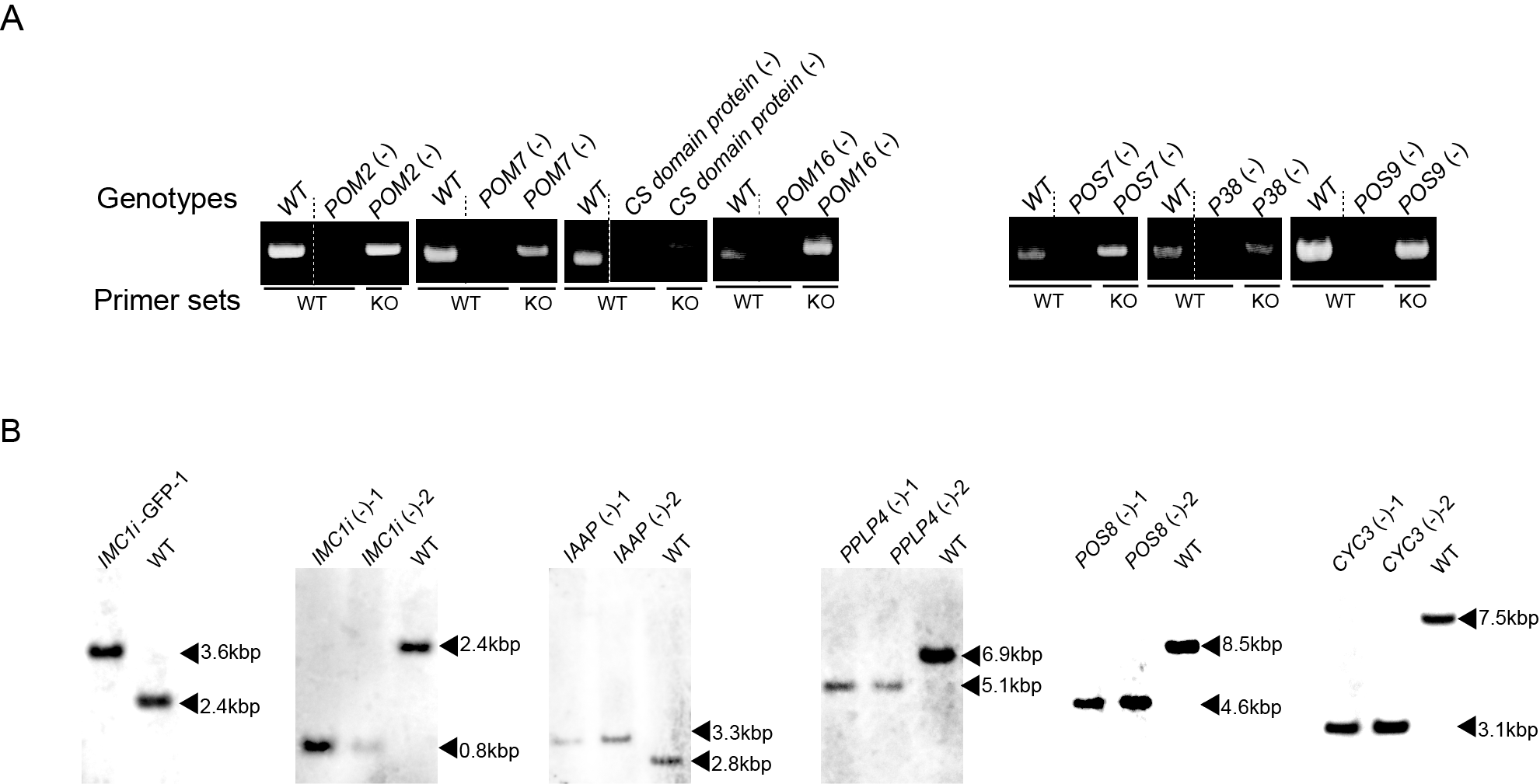

Supplement: S3 Fig — A. Genotypes of all mutant parasites were checked by PCR, using two primer sets for detecting the wild-type (WT) and the knockout (insertion of knockout construct; KO) parasites, respectively. Primers are listed in S14 Table in S2 File. B. When gene disruption resulted in an abnormal phenotype, another independent mutant parasite population was prepared, and the genotypes were confirmed by Southern blot analysis. Primers used for preparing probes are listed in S14 Table in S2 File. (TIF) [file ppat.1004905.s003.tif]

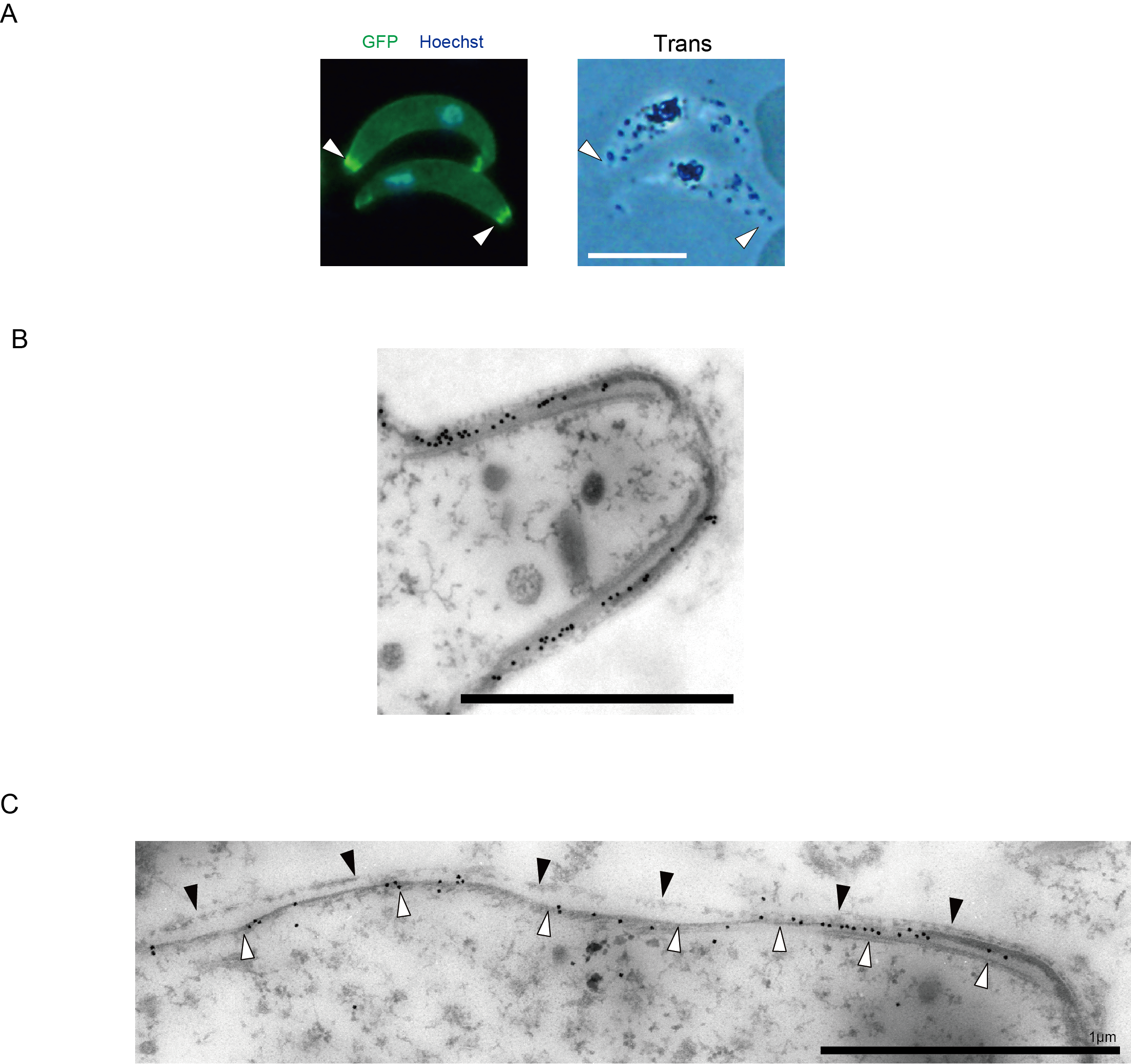

Supplement: S4 Fig — A. Fluorescence microscopy image of ookinetes expressing GFP-tagged PhIL1. Arrows indicates the apical end. Trans, a transmission image. Scale bar, 10 μm. B. Immunoelectron microscopy image of a sagittal section of an ookinete expressing GFP-tagged PhIL1. Colloidal gold particles are mainly localized at the structure of high electron density that is at the apical end adjacent to the IMC and which covers the apical protrusion. The structure seems to be that of the apical cap of ookinetes. Scale bar, 1 μm. C. Immunoelectron microscopy image of a longitudinal section of an ookinete expressing GFP-tagged PhIL1. PhIL1 is also localized at the structure of high electron-density, i.e., IMC (indicated by an open arrow), but not at the plasma membrane (indicated by a closed arrow), in the portion where the plasma membrane was detached from the IMC. (TIF) [file ppat.1004905.s004.tif]

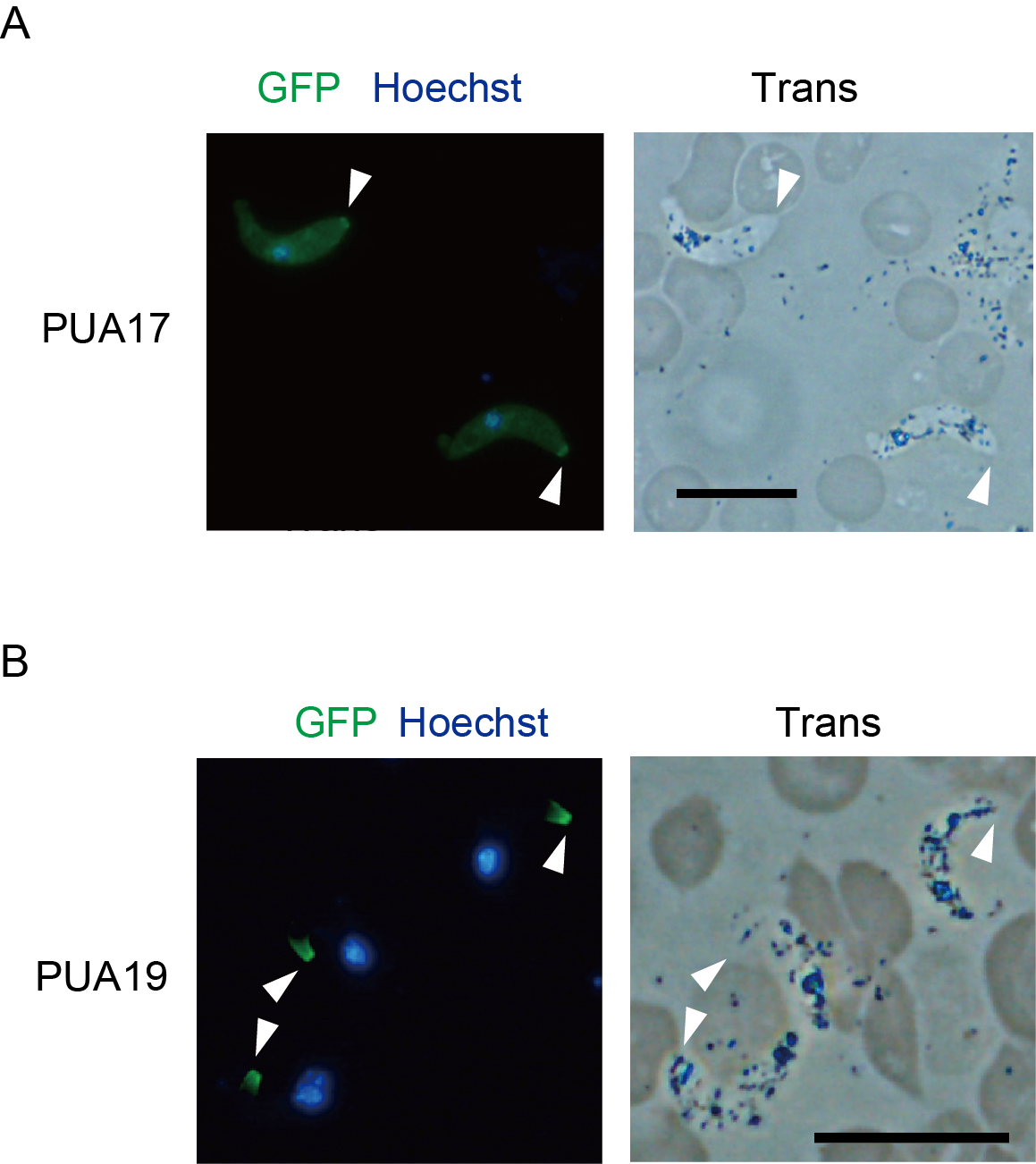

Supplement: S5 Fig — Two genes, viz. PBANKA_083040 (A) and PBANKA_092120 (B) were expressed in P. berghei ookinetes as GFP-tagged proteins under the control of their original promoters, using pCen-GFP. A. Fluorescence microscopy image of ookinetes expressing GFP-tagged PUA17. The product of PUA17 (PBANKA_083040) was distributed at the apical end of mature ookinetes and along the ookinete surface. The distribution pattern was similar to that of PhIL1. Thus, it might be located at the apical cap of ookinetes, but this was difficult to determine further by fluorescence microscopy. Scale bar, 10 μm. PUA17 has orthologs only in coccidian parasites such as Eimeria tenella and Toxoplasma gondii. This gene, designated as G2 (glycine at position 2), is necessary for ookinete motility (Tremp et al., 2013). B. Fluorescence microscopy image of ookinetes expressing GFP-tagged PUA19 (PBANKA_092120). Tagged protein showed a trapezoidal appearance similar to that of ARA1, suggesting localization at an apical structure, such as a conoid or an apical ring. Scale bar, 10 μm. (TIF) [file ppat.1004905.s005.tif]

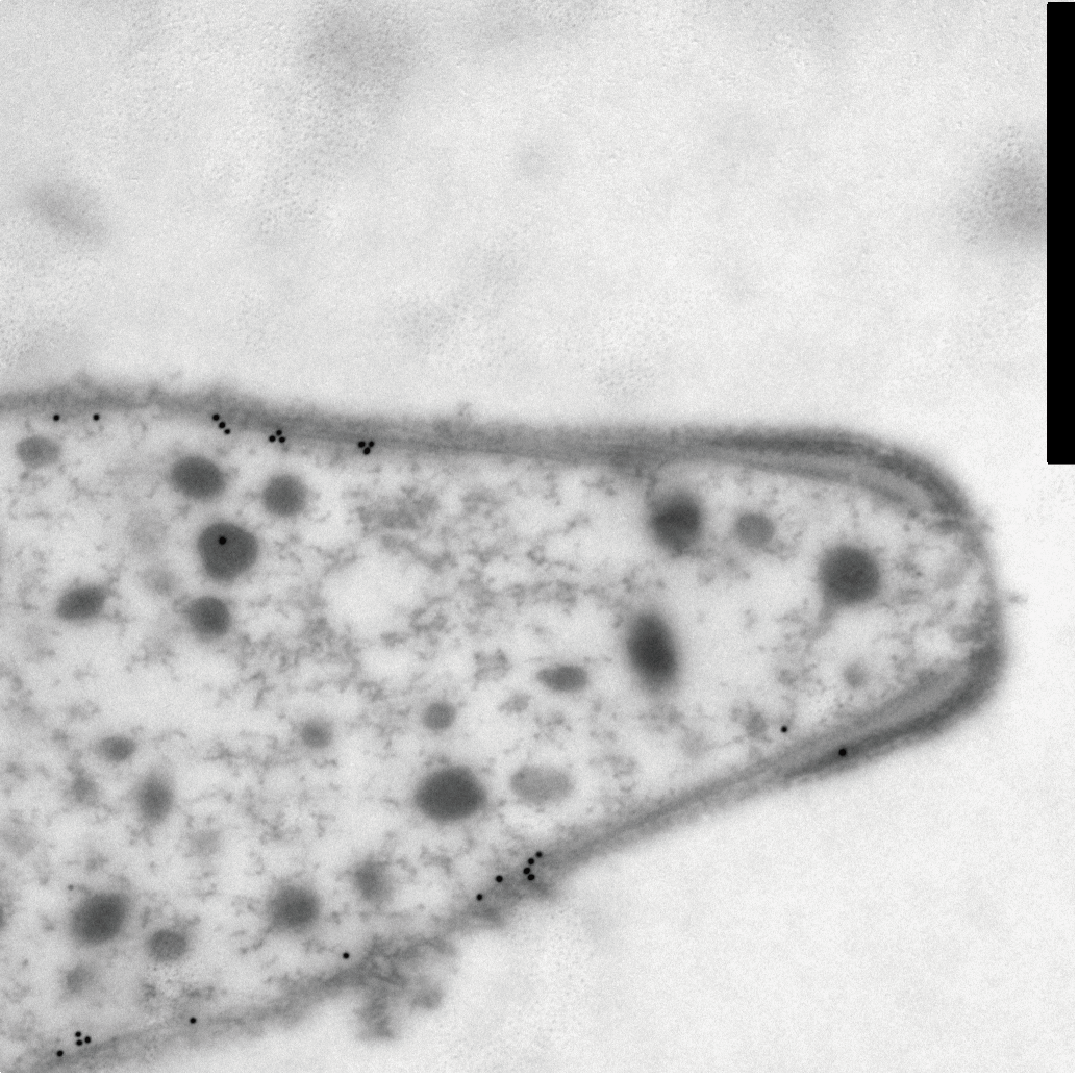

Supplement: S6 Fig — Longitudinal section of an ookinete expressing GFP-tagged PUA26 is shown. Immunoelectron microscopy was performed with anti-GFP antibodies. Colloidal gold particles (15 nm) are mainly localized along the parasite surface except at the apical end. Scale bar, 1 μm. (TIF) [file ppat.1004905.s006.tif]

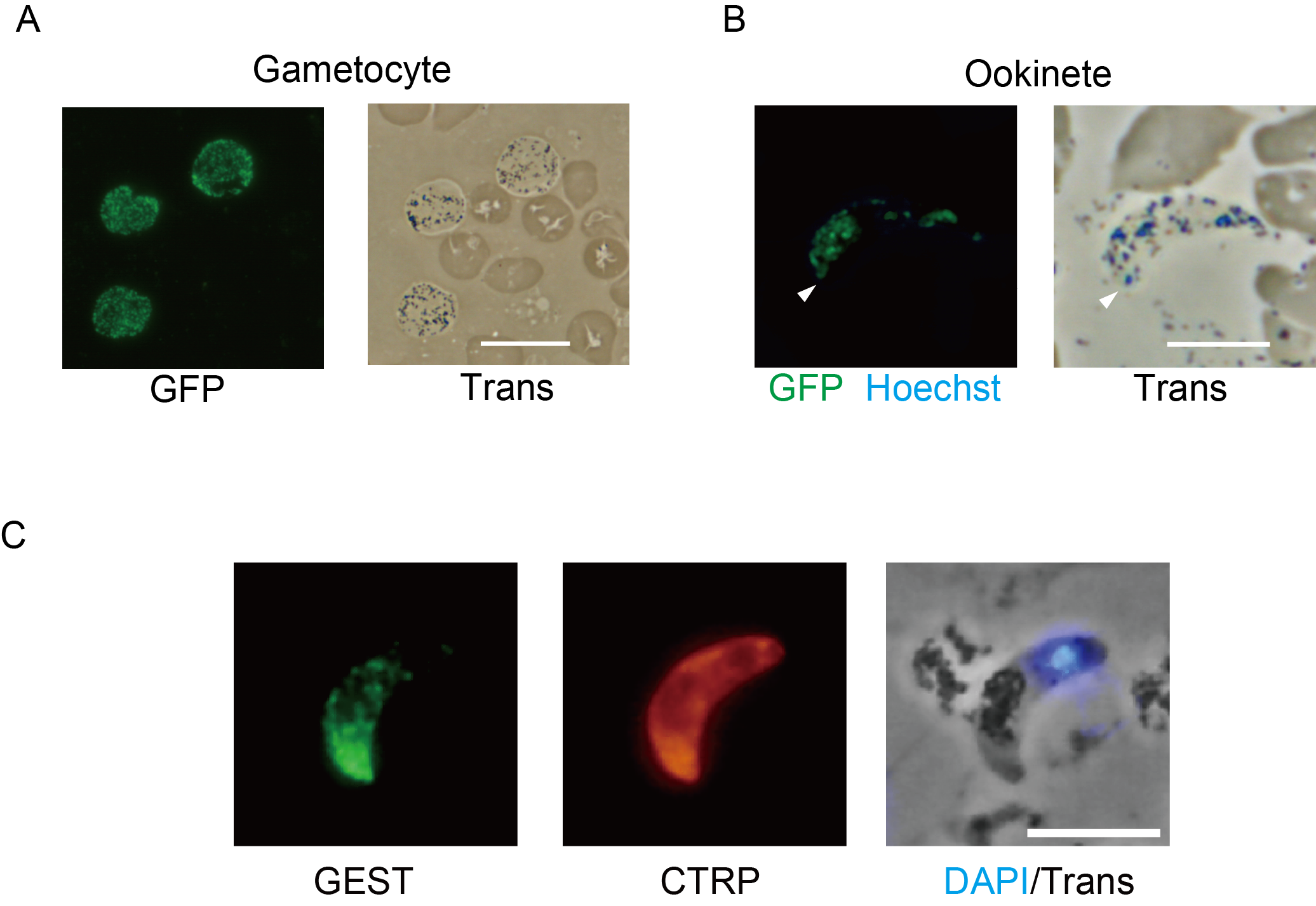

Supplement: S7 Fig — P. berghei parasites expressing GFP-tagged GEST were prepared using pCen-GFP and its expression in ookinetes was investigated. Scale bars, 10 μm. A. Gametocytes in the blood of mice. The tagged protein was observed as particles in the cytoplasm of gametocytes, as previously reported (Talman et al., 2011). B. Ookinetes cultured for 24 h after fertilization. GFP-tagged GEST was observed in vesicle-like particles within the apical portion of the cytoplasm, suggesting that it is a microneme protein. C. Ookinetes were cultured for 22 h after fertilization, fixed with acetone for 1 min, and double-stained with mouse anti-GFP antibody [Dylight 488 (green)] and rabbit anti-CTRP antibody [Dylight 549 (red)]. The nucleus was stained with 4',6-Diamidino-2-phenylindole (DAPI). (TIF) [file ppat.1004905.s007.tif]

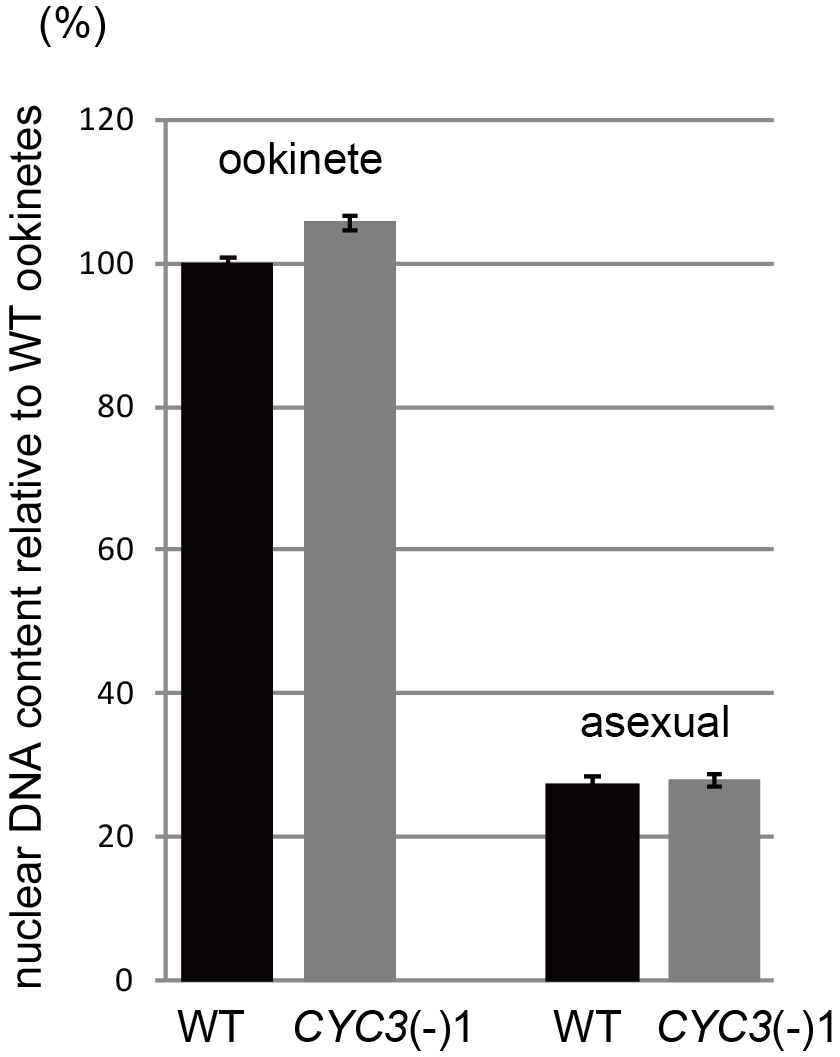

Supplement: S8 Fig — Ookinetes cultured for 24 h were stained with Hoechst 33342. Micrographs were obtained with an Olympus BX60 fluorescence microscope. DNA content of the ookinete nucleus was measured with the AquaCosmos software (Hamamatsu Photonic System). Haploid blood stage parasites were used as controls. In the graph, the values of wild-type ookinetes (tetraploid) were shown as 100%. Values are the mean ± SE of 50 parasites. (TIF) [file ppat.1004905.s008.tif]
