# Supplementary material for: Genome-Wide Identification of the Target Genes of AP2-O, a Plasmodium AP2-Family Transcription Factor
Source: PLoS Pathog. 2015 May 27;11(5):e1004905. doi: 10.1371/journal.ppat.1004905 (PMC4446032; doi:10.1371/journal.ppat.1004905)
Supplement: S1 File — (DOCX) [file ppat.1004905.s009.docx]

**Table S1. Target genes of AP2-O identified in experiment 2**^a^

|  | Gene ID | Functional annotation | In experiment 1 |
| --- | --- | --- | --- |
| 1 | PBANKA_113810 | 14-3-3 protein, putative | Yes |
| 2 | PBANKA_040310 | 1-cys-glutaredoxin-like protein-1, putative (GLP1) | Yes |
| 3 | PBANKA_134780 | 20 kDa chaperonin, putative (CPN20) | Yes |
| 4 | PBANKA_051500 | 25 kDa ookinete surface antigen precursor (P25) | Yes |
| 5 | PBANKA_051490 | 28 kDa ookinete surface protein (P28) | Yes |
| 6 | PBANKA_061750 | 3'-5' exoribonuclease Csl4 homolog, putative | Yes |
| 7 | PBANKA_110420 | 3-methyl-2-oxobutanoate dehydrogenase (lipoamide), putative | Yes |
| 8 | PBANKA_131080 | 40S ribosomal protein S2, putative | Yes |
| 9 | PBANKA_071780 | 60S ribosomal protein L15, putative | Yes |
| 10 | PBANKA_136420 | 60S ribosomal protein L17, putative | No |
| 11 | PBANKA_101950 | 60S ribosomal protein L5, putative | No |
| 12 | PBANKA_101940 | 60S ribosomal protein L7-3, putative | No |
| 13 | PBANKA_110760 | 6-cysteine protein (P38) | Yes |
| 14 | PBANKA_100550 | AAA family ATPase, putative | No |
| 15 | PBANKA_110310 | actin-depolymerizing factor 1 (ADF1) | Yes |
| 16 | PBANKA_061680 | actin-related protein | Yes |
| 17 | PBANKA_020930 | actin-related protein (ARP1) | Yes |
| 18 | PBANKA_111870 | acyl-CoA synthetase, putative | Yes |
| 19 | PBANKA_092910 | acyl-CoA-binding protein, putative | Yes |
| 20 | PBANKA_071760 | adenylate kinase 1, putative | Yes |
| 21 | PBANKA_113110 | adenylosuccinate synthetase, putative | Yes |
| 22 | PBANKA_090600 | alpha/beta hydrolase, putative | Yes |
| 23 | PBANKA_112340 | anaphase-promoting complex subunit, putative | Yes |
| 24 | PBANKA_070320 | apicoplast ribosomal protein L21 precursor, putative | Yes |
| 25 | PBANKA_040760 | asparagine synthetase, putative | No |
| 26 | PBANKA_021020 | aspartyl-tRNA synthetase, putative | Yes |
| 27 | PBANKA_145030 | ATP synthase subunit beta, mitochondrial, putative | Yes |
| 28 | PBANKA_112260 | ATP-dependent DEAD box helicase, putative | Yes |
| 29 | PBANKA_101480 | ATP-dependent DNA helicase, putative | Yes |
| 30 | PBANKA_091990 | ATP-dependent phosphofructokinase, putative | No |
| 31 | PBANKA_134750 | ATP-dependent RNA helicase, putative | No |
| 32 | PBANKA_101320 | ATP-specific succinyl-CoA synthetase beta subunit, putative | Yes |
| 33 | PBANKA_050410 | autophagy-related protein 8, putative (ATG8) | No |
| 34 | PBANKA_133820 | beta-hydroxyacyl-ACP dehydratase, putative (FabZ) | Yes |
| 35 | PBANKA_051100 | biotin--acetyl-CoA-carboxylase, putative | Yes |
| 36 | PBANKA_103640 | BOP1-like protein, putative | Yes |
| 37 | PBANKA_031420 | calcium dependent protein kinase 1 (CDPK1) | Yes |
| 38 | PBANKA_040820 | calcium dependent protein kinase 3 (CDPK3) | Yes |
| 39 | PBANKA_010420 | calcium-binding protein, putative | Yes |
| 40 | PBANKA_123430 | carbon catabolite repressor protein 4, putative (CCR4) | Yes |
| 41 | PBANKA_113320 | cdc2-related kinase 2 (CRK2) | Yes |
| 42 | PBANKA_071870 | CDGSH iron-sulfur domain-containing protein, putative | Yes |
| 43 | PBANKA_111190 | CDK-activating kinase assembly factor, putative (MAT1) | Yes |
| 44 | PBANKA_143230 | cell traversal protein for ookinetes and sporozoites (CelTOS) | Yes |
| 45 | PBANKA_080050 | chitinase (CHT1) | Yes |
| 46 | PBANKA_112190 | chorismate synthase, putative (CS) | Yes |
| 47 | PBANKA_041290 | circumsporozoite- and TRAP-related protein (CTRP) | Yes |
| 48 | PBANKA_031400 | clathrin coat assembly protein, putative | Yes |
| 49 | PBANKA_070920 | clp1-related protein, putative | Yes |
| 50 | PBANKA_122230 | coatomer epsilon subunit, putative | Yes |
| 51 | PBANKA_020370 | conserved Plasmodium membrane protein, unknown function | Yes |
| 52 | PBANKA_010450 | conserved Plasmodium protein, unknown function | Yes |
| 53 | PBANKA_010640 | conserved Plasmodium protein, unknown function | Yes |
| 54 | PBANKA_010650 | conserved Plasmodium protein, unknown function | Yes |
| 55 | PBANKA_010700 | conserved Plasmodium protein, unknown function | Yes |
| 56 | PBANKA_011160 | conserved Plasmodium protein, unknown function | Yes |
| 57 | PBANKA_020170 | conserved Plasmodium protein, unknown function | Yes |
| 58 | PBANKA_020220 | conserved Plasmodium protein, unknown function | Yes |
| 59 | PBANKA_020240 | conserved Plasmodium protein, unknown function | Yes |
| 60 | PBANKA_020680 | conserved Plasmodium protein, unknown function | Yes |
| 61 | PBANKA_020850 | conserved Plasmodium protein, unknown function | Yes |
| 62 | PBANKA_020860 | conserved Plasmodium protein, unknown function | Yes |
| 63 | PBANKA_021000 | conserved Plasmodium protein, unknown function | No |
| 64 | PBANKA_021030 | conserved Plasmodium protein, unknown function | Yes |
| 65 | PBANKA_021110 | conserved Plasmodium protein, unknown function | Yes |
| 66 | PBANKA_021290 | conserved Plasmodium protein, unknown function | No |
| 67 | PBANKA_030310 | conserved Plasmodium protein, unknown function | Yes |
| 68 | PBANKA_030450 | conserved Plasmodium protein, unknown function | No |
| 69 | PBANKA_030550 | conserved Plasmodium protein, unknown function | Yes |
| 70 | PBANKA_031150 | conserved Plasmodium protein, unknown function | Yes |
| 71 | PBANKA_031200 | conserved Plasmodium protein, unknown function | Yes |
| 72 | PBANKA_031350 | conserved Plasmodium protein, unknown function | Yes |
| 73 | PBANKA_031370 | conserved Plasmodium protein, unknown function | Yes |
| 74 | PBANKA_040170 | conserved Plasmodium protein, unknown function | Yes |
| 75 | PBANKA_040580 | conserved Plasmodium protein, unknown function | Yes |
| 76 | PBANKA_040590 | conserved Plasmodium protein, unknown function | Yes |
| 77 | PBANKA_040690 | conserved Plasmodium protein, unknown function | Yes |
| 78 | PBANKA_040890 | conserved Plasmodium protein, unknown function | No |
| 79 | PBANKA_040900 | conserved Plasmodium protein, unknown function | Yes |
| 80 | PBANKA_041065 | conserved Plasmodium protein, unknown function | Yes |
| 81 | PBANKA_041420 | conserved Plasmodium protein, unknown function | Yes |
| 82 | PBANKA_041430 | conserved Plasmodium protein, unknown function | Yes |
| 83 | PBANKA_041720 | conserved Plasmodium protein, unknown function | Yes |
| 84 | PBANKA_050300 | conserved Plasmodium protein, unknown function | Yes |
| 85 | PBANKA_050380 | conserved Plasmodium protein, unknown function | Yes |
| 86 | PBANKA_050440 | conserved Plasmodium protein, unknown function | Yes |
| 87 | PBANKA_050470 | conserved Plasmodium protein, unknown function | Yes |
| 88 | PBANKA_050520 | conserved Plasmodium protein, unknown function | Yes |
| 89 | PBANKA_050530 | conserved Plasmodium protein, unknown function | Yes |
| 90 | PBANKA_050540 | conserved Plasmodium protein, unknown function | Yes |
| 91 | PBANKA_050550 | conserved Plasmodium protein, unknown function | Yes |
| 92 | PBANKA_050600 | conserved Plasmodium protein, unknown function | Yes |
| 93 | PBANKA_050720 | conserved Plasmodium protein, unknown function | Yes |
| 94 | PBANKA_050820 | conserved Plasmodium protein, unknown function | Yes |
| 95 | PBANKA_050830 | conserved Plasmodium protein, unknown function | No |
| 96 | PBANKA_050860 | conserved Plasmodium protein, unknown function | Yes |
| 97 | PBANKA_050870 | conserved Plasmodium protein, unknown function | Yes |
| 98 | PBANKA_050880 | conserved Plasmodium protein, unknown function | Yes |
| 99 | PBANKA_050890 | conserved Plasmodium protein, unknown function | Yes |
| 100 | PBANKA_050950 | conserved Plasmodium protein, unknown function | Yes |
| 101 | PBANKA_050960 | conserved Plasmodium protein, unknown function | Yes |
| 102 | PBANKA_051040 | conserved Plasmodium protein, unknown function | Yes |
| 103 | PBANKA_051050 | conserved Plasmodium protein, unknown function | Yes |
| 104 | PBANKA_051070 | conserved Plasmodium protein, unknown function | Yes |
| 105 | PBANKA_051530 | conserved Plasmodium protein, unknown function | Yes |
| 106 | PBANKA_051810 | conserved Plasmodium protein, unknown function | Yes |
| 107 | PBANKA_052230 | conserved Plasmodium protein, unknown function | No |
| 108 | PBANKA_052370 | conserved Plasmodium protein, unknown function | No |
| 109 | PBANKA_060150 | conserved Plasmodium protein, unknown function | Yes |
| 110 | PBANKA_060300 | conserved Plasmodium protein, unknown function | Yes |
| 111 | PBANKA_060310 | conserved Plasmodium protein, unknown function | Yes |
| 112 | PBANKA_060340 | conserved Plasmodium protein, unknown function | Yes |
| 113 | PBANKA_060350 | conserved Plasmodium protein, unknown function | Yes |
| 114 | PBANKA_060400 | conserved Plasmodium protein, unknown function | Yes |
| 115 | PBANKA_061070 | conserved Plasmodium protein, unknown function | No |
| 116 | PBANKA_061120 | conserved Plasmodium protein, unknown function | Yes |
| 117 | PBANKA_061150 | conserved Plasmodium protein, unknown function | Yes |
| 118 | PBANKA_061210 | conserved Plasmodium protein, unknown function | Yes |
| 119 | PBANKA_061240 | conserved Plasmodium protein, unknown function | Yes |
| 120 | PBANKA_061290 | conserved Plasmodium protein, unknown function | No |
| 121 | PBANKA_061300 | conserved Plasmodium protein, unknown function | Yes |
| 122 | PBANKA_061620 | conserved Plasmodium protein, unknown function | No |
| 123 | PBANKA_061640 | conserved Plasmodium protein, unknown function | Yes |
| 124 | PBANKA_061650 | conserved Plasmodium protein, unknown function | Yes |
| 125 | PBANKA_061780 | conserved Plasmodium protein, unknown function | Yes |
| 126 | PBANKA_061850 | conserved Plasmodium protein, unknown function | Yes |
| 127 | PBANKA_061870 | conserved Plasmodium protein, unknown function | Yes |
| 128 | PBANKA_062060 | conserved Plasmodium protein, unknown function | Yes |
| 129 | PBANKA_062070 | conserved Plasmodium protein, unknown function | Yes |
| 130 | PBANKA_062090 | conserved Plasmodium protein, unknown function | Yes |
| 131 | PBANKA_062100 | conserved Plasmodium protein, unknown function | Yes |
| 132 | PBANKA_062110 | conserved Plasmodium protein, unknown function | Yes |
| 133 | PBANKA_062150 | conserved Plasmodium protein, unknown function | Yes |
| 134 | PBANKA_062230 | conserved Plasmodium protein, unknown function | Yes |
| 135 | PBANKA_070480 | conserved Plasmodium protein, unknown function | Yes |
| 136 | PBANKA_070490 | conserved Plasmodium protein, unknown function | Yes |
| 137 | PBANKA_070660 | conserved Plasmodium protein, unknown function | Yes |
| 138 | PBANKA_070850 | conserved Plasmodium protein, unknown function | No |
| 139 | PBANKA_070960 | conserved Plasmodium protein, unknown function | Yes |
| 140 | PBANKA_071110 | conserved Plasmodium protein, unknown function | Yes |
| 141 | PBANKA_071250 | conserved Plasmodium protein, unknown function | Yes |
| 142 | PBANKA_071320 | conserved Plasmodium protein, unknown function | Yes |
| 143 | PBANKA_071450 | conserved Plasmodium protein, unknown function | Yes |
| 144 | PBANKA_071680 | conserved Plasmodium protein, unknown function | Yes |
| 145 | PBANKA_071910 | conserved Plasmodium protein, unknown function | Yes |
| 146 | PBANKA_072090 | conserved Plasmodium protein, unknown function | Yes |
| 147 | PBANKA_072100 | conserved Plasmodium protein, unknown function | Yes |
| 148 | PBANKA_080720 | conserved Plasmodium protein, unknown function | Yes |
| 149 | PBANKA_081020 | conserved Plasmodium protein, unknown function | Yes |
| 150 | PBANKA_081030 | conserved Plasmodium protein, unknown function | Yes |
| 151 | PBANKA_081480 | conserved Plasmodium protein, unknown function | Yes |
| 152 | PBANKA_081620 | conserved Plasmodium protein, unknown function | Yes |
| 153 | PBANKA_081650 | conserved Plasmodium protein, unknown function | Yes |
| 154 | PBANKA_081690 | conserved Plasmodium protein, unknown function | Yes |
| 155 | PBANKA_081840 | conserved Plasmodium protein, unknown function | Yes |
| 156 | PBANKA_082120 | conserved Plasmodium protein, unknown function | Yes |
| 157 | PBANKA_082130 | conserved Plasmodium protein, unknown function | Yes |
| 158 | PBANKA_082150 | conserved Plasmodium protein, unknown function | Yes |
| 159 | PBANKA_082320 | conserved Plasmodium protein, unknown function | No |
| 160 | PBANKA_082500 | conserved Plasmodium protein, unknown function | Yes |
| 161 | PBANKA_082510 | conserved Plasmodium protein, unknown function | Yes |
| 162 | PBANKA_082590 | conserved Plasmodium protein, unknown function | Yes |
| 163 | PBANKA_082920 | conserved Plasmodium protein, unknown function | Yes |
| 164 | PBANKA_082950 | conserved Plasmodium protein, unknown function | Yes |
| 165 | PBANKA_082990 | conserved Plasmodium protein, unknown function | Yes |
| 166 | PBANKA_083040 | conserved Plasmodium protein, unknown function | Yes |
| 167 | PBANKA_083080 | conserved Plasmodium protein, unknown function | Yes |
| 168 | PBANKA_083240 | conserved Plasmodium protein, unknown function | No |
| 169 | PBANKA_083280 | conserved Plasmodium protein, unknown function | Yes |
| 170 | PBANKA_083370 | conserved Plasmodium protein, unknown function | Yes |
| 171 | PBANKA_083420 | conserved Plasmodium protein, unknown function | Yes |
| 172 | PBANKA_083470 | conserved Plasmodium protein, unknown function | Yes |
| 173 | PBANKA_083590 | conserved Plasmodium protein, unknown function | Yes |
| 174 | PBANKA_090320 | conserved Plasmodium protein, unknown function | Yes |
| 175 | PBANKA_090610 | conserved Plasmodium protein, unknown function | Yes |
| 176 | PBANKA_090680 | conserved Plasmodium protein, unknown function | No |
| 177 | PBANKA_090700 | conserved Plasmodium protein, unknown function | Yes |
| 178 | PBANKA_090860 | conserved Plasmodium protein, unknown function | Yes |
| 179 | PBANKA_091540 | conserved Plasmodium protein, unknown function | Yes |
| 180 | PBANKA_091600 | conserved Plasmodium protein, unknown function | Yes |
| 181 | PBANKA_091650 | conserved Plasmodium protein, unknown function | Yes |
| 182 | PBANKA_091670 | conserved Plasmodium protein, unknown function | Yes |
| 183 | PBANKA_091690 | conserved Plasmodium protein, unknown function | Yes |
| 184 | PBANKA_091710 | conserved Plasmodium protein, unknown function | Yes |
| 185 | PBANKA_091770 | conserved Plasmodium protein, unknown function | Yes |
| 186 | PBANKA_092030 | conserved Plasmodium protein, unknown function | No |
| 187 | PBANKA_092120 | conserved Plasmodium protein, unknown function | Yes |
| 188 | PBANKA_092160 | conserved Plasmodium protein, unknown function | Yes |
| 189 | PBANKA_092240 | conserved Plasmodium protein, unknown function | Yes |
| 190 | PBANKA_092250 | conserved Plasmodium protein, unknown function | Yes |
| 191 | PBANKA_092400 | conserved Plasmodium protein, unknown function | Yes |
| 192 | PBANKA_092450 | conserved Plasmodium protein, unknown function | Yes |
| 193 | PBANKA_092560 | conserved Plasmodium protein, unknown function | Yes |
| 194 | PBANKA_092800 | conserved Plasmodium protein, unknown function | Yes |
| 195 | PBANKA_093010 | conserved Plasmodium protein, unknown function | Yes |
| 196 | PBANKA_093110 | conserved Plasmodium protein, unknown function | Yes |
| 197 | PBANKA_093660 | conserved Plasmodium protein, unknown function | Yes |
| 198 | PBANKA_093670 | conserved Plasmodium protein, unknown function | Yes |
| 199 | PBANKA_093750 | conserved Plasmodium protein, unknown function | Yes |
| 200 | PBANKA_093880 | conserved Plasmodium protein, unknown function | Yes |
| 201 | PBANKA_094100 | conserved Plasmodium protein, unknown function | No |
| 202 | PBANKA_094110 | conserved Plasmodium protein, unknown function | Yes |
| 203 | PBANKA_094170 | conserved Plasmodium protein, unknown function | No |
| 204 | PBANKA_100640 | conserved Plasmodium protein, unknown function | Yes |
| 205 | PBANKA_100990 | conserved Plasmodium protein, unknown function | Yes |
| 206 | PBANKA_101260 | conserved Plasmodium protein, unknown function | Yes |
| 207 | PBANKA_101390 | conserved Plasmodium protein, unknown function | Yes |
| 208 | PBANKA_101470 | conserved Plasmodium protein, unknown function | Yes |
| 209 | PBANKA_101580 | conserved Plasmodium protein, unknown function | Yes |
| 210 | PBANKA_101900 | conserved Plasmodium protein, unknown function | Yes |
| 211 | PBANKA_101920 | conserved Plasmodium protein, unknown function | Yes |
| 212 | PBANKA_101935 | conserved Plasmodium protein, unknown function | Yes |
| 213 | PBANKA_102050 | conserved Plasmodium protein, unknown function | Yes |
| 214 | PBANKA_102080 | conserved Plasmodium protein, unknown function | Yes |
| 215 | PBANKA_102100 | conserved Plasmodium protein, unknown function | Yes |
| 216 | PBANKA_102120 | conserved Plasmodium protein, unknown function | Yes |
| 217 | PBANKA_102380 | conserved Plasmodium protein, unknown function | Yes |
| 218 | PBANKA_102570 | conserved Plasmodium protein, unknown function | Yes |
| 219 | PBANKA_103890 | conserved Plasmodium protein, unknown function | Yes |
| 220 | PBANKA_110390 | conserved Plasmodium protein, unknown function | Yes |
| 221 | PBANKA_110590 | conserved Plasmodium protein, unknown function | Yes |
| 222 | PBANKA_110690 | conserved Plasmodium protein, unknown function | Yes |
| 223 | PBANKA_110750 | conserved Plasmodium protein, unknown function | Yes |
| 224 | PBANKA_110910 | conserved Plasmodium protein, unknown function | Yes |
| 225 | PBANKA_110990 | conserved Plasmodium protein, unknown function | Yes |
| 226 | PBANKA_111000 | conserved Plasmodium protein, unknown function | Yes |
| 227 | PBANKA_111200 | conserved Plasmodium protein, unknown function | Yes |
| 228 | PBANKA_111610 | conserved Plasmodium protein, unknown function | Yes |
| 229 | PBANKA_111640 | conserved Plasmodium protein, unknown function | Yes |
| 230 | PBANKA_111680 | conserved Plasmodium protein, unknown function | Yes |
| 231 | PBANKA_111760 | conserved Plasmodium protein, unknown function | Yes |
| 232 | PBANKA_111820 | conserved Plasmodium protein, unknown function | No |
| 233 | PBANKA_111830 | conserved Plasmodium protein, unknown function | No |
| 234 | PBANKA_111840 | conserved Plasmodium protein, unknown function | Yes |
| 235 | PBANKA_111880 | conserved Plasmodium protein, unknown function | Yes |
| 236 | PBANKA_111920 | conserved Plasmodium protein, unknown function | Yes |
| 237 | PBANKA_111980 | conserved Plasmodium protein, unknown function | Yes |
| 238 | PBANKA_112010 | conserved Plasmodium protein, unknown function | Yes |
| 239 | PBANKA_112100 | conserved Plasmodium protein, unknown function | No |
| 240 | PBANKA_112250 | conserved Plasmodium protein, unknown function | Yes |
| 241 | PBANKA_112310 | conserved Plasmodium protein, unknown function | Yes |
| 242 | PBANKA_112320 | conserved Plasmodium protein, unknown function | Yes |
| 243 | PBANKA_112330 | conserved Plasmodium protein, unknown function | Yes |
| 244 | PBANKA_112460 | conserved Plasmodium protein, unknown function | Yes |
| 245 | PBANKA_112470 | conserved Plasmodium protein, unknown function | Yes |
| 246 | PBANKA_112950 | conserved Plasmodium protein, unknown function | Yes |
| 247 | PBANKA_113150 | conserved Plasmodium protein, unknown function | Yes |
| 248 | PBANKA_114140 | conserved Plasmodium protein, unknown function | Yes |
| 249 | PBANKA_114160 | conserved Plasmodium protein, unknown function | No |
| 250 | PBANKA_114190 | conserved Plasmodium protein, unknown function | Yes |
| 251 | PBANKA_114380 | conserved Plasmodium protein, unknown function | Yes |
| 252 | PBANKA_120070 | conserved Plasmodium protein, unknown function | Yes |
| 253 | PBANKA_120450 | conserved Plasmodium protein, unknown function | Yes |
| 254 | PBANKA_120460 | conserved Plasmodium protein, unknown function | Yes |
| 255 | PBANKA_120470 | conserved Plasmodium protein, unknown function | Yes |
| 256 | PBANKA_120940 | conserved Plasmodium protein, unknown function | Yes |
| 257 | PBANKA_120970 | conserved Plasmodium protein, unknown function | Yes |
| 258 | PBANKA_122080 | conserved Plasmodium protein, unknown function | Yes |
| 259 | PBANKA_122140 | conserved Plasmodium protein, unknown function | Yes |
| 260 | PBANKA_122170 | conserved Plasmodium protein, unknown function | No |
| 261 | PBANKA_122180 | conserved Plasmodium protein, unknown function | No |
| 262 | PBANKA_122270 | conserved Plasmodium protein, unknown function | Yes |
| 263 | PBANKA_122320 | conserved Plasmodium protein, unknown function | Yes |
| 264 | PBANKA_122540 | conserved Plasmodium protein, unknown function | Yes |
| 265 | PBANKA_122610 | conserved Plasmodium protein, unknown function | Yes |
| 266 | PBANKA_122730 | conserved Plasmodium protein, unknown function | No |
| 267 | PBANKA_122780 | conserved Plasmodium protein, unknown function | Yes |
| 268 | PBANKA_122790 | conserved Plasmodium protein, unknown function | Yes |
| 269 | PBANKA_122830 | conserved Plasmodium protein, unknown function | Yes |
| 270 | PBANKA_122910 | conserved Plasmodium protein, unknown function | Yes |
| 271 | PBANKA_123060 | conserved Plasmodium protein, unknown function | Yes |
| 272 | PBANKA_123070 | conserved Plasmodium protein, unknown function | Yes |
| 273 | PBANKA_123120 | conserved Plasmodium protein, unknown function | Yes |
| 274 | PBANKA_123200 | conserved Plasmodium protein, unknown function | No |
| 275 | PBANKA_123280 | conserved Plasmodium protein, unknown function | Yes |
| 276 | PBANKA_123370 | conserved Plasmodium protein, unknown function | Yes |
| 277 | PBANKA_123440 | conserved Plasmodium protein, unknown function | Yes |
| 278 | PBANKA_123460 | conserved Plasmodium protein, unknown function | Yes |
| 279 | PBANKA_123570 | conserved Plasmodium protein, unknown function | No |
| 280 | PBANKA_123580 | conserved Plasmodium protein, unknown function | No |
| 281 | PBANKA_123600 | conserved Plasmodium protein, unknown function | Yes |
| 282 | PBANKA_124010 | conserved Plasmodium protein, unknown function | Yes |
| 283 | PBANKA_124140 | conserved Plasmodium protein, unknown function | Yes |
| 284 | PBANKA_124260 | conserved Plasmodium protein, unknown function | No |
| 285 | PBANKA_124290 | conserved Plasmodium protein, unknown function | No |
| 286 | PBANKA_124320 | conserved Plasmodium protein, unknown function | No |
| 287 | PBANKA_124350 | conserved Plasmodium protein, unknown function | Yes |
| 288 | PBANKA_131130 | conserved Plasmodium protein, unknown function | Yes |
| 289 | PBANKA_131240 | conserved Plasmodium protein, unknown function | Yes |
| 290 | PBANKA_131280 | conserved Plasmodium protein, unknown function | No |
| 291 | PBANKA_131310 | conserved Plasmodium protein, unknown function | Yes |
| 292 | PBANKA_131860 | conserved Plasmodium protein, unknown function | Yes |
| 293 | PBANKA_132430 | conserved Plasmodium protein, unknown function | No |
| 294 | PBANKA_133080 | conserved Plasmodium protein, unknown function | No |
| 295 | PBANKA_133660 | conserved Plasmodium protein, unknown function | Yes |
| 296 | PBANKA_133680 | conserved Plasmodium protein, unknown function | No |
| 297 | PBANKA_133930 | conserved Plasmodium protein, unknown function | Yes |
| 298 | PBANKA_133950 | conserved Plasmodium protein, unknown function | Yes |
| 299 | PBANKA_134250 | conserved Plasmodium protein, unknown function | Yes |
| 300 | PBANKA_134420 | conserved Plasmodium protein, unknown function | No |
| 301 | PBANKA_134550 | conserved Plasmodium protein, unknown function | Yes |
| 302 | PBANKA_134700 | conserved Plasmodium protein, unknown function | Yes |
| 303 | PBANKA_134730 | conserved Plasmodium protein, unknown function | Yes |
| 304 | PBANKA_134820 | conserved Plasmodium protein, unknown function | Yes |
| 305 | PBANKA_134850 | conserved Plasmodium protein, unknown function | Yes |
| 306 | PBANKA_134870 | conserved Plasmodium protein, unknown function | Yes |
| 307 | PBANKA_135170 | conserved Plasmodium protein, unknown function | Yes |
| 308 | PBANKA_135230 | conserved Plasmodium protein, unknown function | Yes |
| 309 | PBANKA_135240 | conserved Plasmodium protein, unknown function | Yes |
| 310 | PBANKA_135250 | conserved Plasmodium protein, unknown function | Yes |
| 311 | PBANKA_135310 | conserved Plasmodium protein, unknown function | Yes |
| 312 | PBANKA_135380 | conserved Plasmodium protein, unknown function | Yes |
| 313 | PBANKA_135460 | conserved Plasmodium protein, unknown function | Yes |
| 314 | PBANKA_135490 | conserved Plasmodium protein, unknown function | Yes |
| 315 | PBANKA_135950 | conserved Plasmodium protein, unknown function | Yes |
| 316 | PBANKA_136270 | conserved Plasmodium protein, unknown function | Yes |
| 317 | PBANKA_136360 | conserved Plasmodium protein, unknown function | Yes |
| 318 | PBANKA_136410 | conserved Plasmodium protein, unknown function | No |
| 319 | PBANKA_136440 | conserved Plasmodium protein, unknown function | Yes |
| 320 | PBANKA_140440 | conserved Plasmodium protein, unknown function | Yes |
| 321 | PBANKA_140690 | conserved Plasmodium protein, unknown function | No |
| 322 | PBANKA_140820 | conserved Plasmodium protein, unknown function | Yes |
| 323 | PBANKA_140920 | conserved Plasmodium protein, unknown function | Yes |
| 324 | PBANKA_141740 | conserved Plasmodium protein, unknown function | No |
| 325 | PBANKA_142280 | conserved Plasmodium protein, unknown function | Yes |
| 326 | PBANKA_142290 | conserved Plasmodium protein, unknown function | Yes |
| 327 | PBANKA_142430 | conserved Plasmodium protein, unknown function | Yes |
| 328 | PBANKA_143520 | conserved Plasmodium protein, unknown function | Yes |
| 329 | PBANKA_143770 | conserved Plasmodium protein, unknown function | Yes |
| 330 | PBANKA_143780 | conserved Plasmodium protein, unknown function | Yes |
| 331 | PBANKA_144120 | conserved Plasmodium protein, unknown function | Yes |
| 332 | PBANKA_144360 | conserved Plasmodium protein, unknown function | Yes |
| 333 | PBANKA_144370 | conserved Plasmodium protein, unknown function | Yes |
| 334 | PBANKA_144460 | conserved Plasmodium protein, unknown function | Yes |
| 335 | PBANKA_144470 | conserved Plasmodium protein, unknown function | Yes |
| 336 | PBANKA_144880 | conserved Plasmodium protein, unknown function | No |
| 337 | PBANKA_145080 | conserved Plasmodium protein, unknown function | Yes |
| 338 | PBANKA_145120 | conserved Plasmodium protein, unknown function | Yes |
| 339 | PBANKA_145150 | conserved Plasmodium protein, unknown function | Yes |
| 340 | PBANKA_145670 | conserved Plasmodium protein, unknown function | Yes |
| 341 | PBANKA_145770 | conserved Plasmodium protein, unknown function | Yes |
| 342 | PBANKA_145910 | conserved Plasmodium protein, unknown function | Yes |
| 343 | PBANKA_145980 | conserved Plasmodium protein, unknown function | Yes |
| 344 | PBANKA_146100 | conserved Plasmodium protein, unknown function | Yes |
| 345 | PBANKA_146120 | conserved Plasmodium protein, unknown function | Yes |
| 346 | PBANKA_146130 | conserved Plasmodium protein, unknown function | Yes |
| 347 | PBANKA_146390 | conserved Plasmodium protein, unknown function | Yes |
| 348 | PBANKA_071900 | conserved Plasmodium protein, unknown function, fragment | Yes |
| 349 | PBANKA_090230 | conserved Plasmodium protein, unknown function, fragment | Yes |
| 350 | PBANKA_091700 | conserved Plasmodium protein, unknown function, fragment | Yes |
| 351 | PBANKA_041540 | conserved Plasmodium protein, unknown function, pseudogene | Yes |
| 352 | PBANKA_031060 | conserved protein, unknown function | Yes |
| 353 | PBANKA_020090 | conserved rodent malaria protein, unknown function | Yes |
| 354 | PBANKA_122220 | conserved rodent malaria protein, unknown function | No |
| 355 | PBANKA_101700 | CorA-like Mg2 transporter protein, putative | Yes |
| 356 | PBANKA_082110 | CS domain protein, putative | Yes |
| 357 | PBANKA_123320 | cyclin, putative | Yes |
| 358 | PBANKA_081910 | cytochrome b5, putative | Yes |
| 359 | PBANKA_061880 | DEAD box helicase, putative | Yes |
| 360 | PBANKA_123630 | DEAD-box subfamily ATP-dependent helicase, putative | Yes |
| 361 | PBANKA_050580 | deoxyribose-phosphate aldolase, putative | Yes |
| 362 | PBANKA_134000 | dihydrofolate synthase/folylpolyglutamate synthase, putative | Yes |
| 363 | PBANKA_050500 | dihydrolipoamide acyltransferase, putative | Yes |
| 364 | PBANKA_081970 | dihydrouridine synthase, putative | Yes |
| 365 | PBANKA_093130 | dipeptidyl aminopeptidase, putative | Yes |
| 366 | PBANKA_083490 | DNA excision-repair helicase, putative | Yes |
| 367 | PBANKA_021040 | DNA mismatch repair protein, putative | Yes |
| 368 | PBANKA_122680 | DNA repair protein rad54, putative | Yes |
| 369 | PBANKA_071830 | DNA replication licensing factor, putative | Yes |
| 370 | PBANKA_101910 | DNA-directed DNA polymerase, putative | Yes |
| 371 | PBANKA_030320 | DNA-directed RNA polymerase II 16 kDa subunit, putative | Yes |
| 372 | PBANKA_134530 | DNAJ like protein, putative | No |
| 373 | PBANKA_102110 | DnaJ protein, putative | Yes |
| 374 | PBANKA_030840 | dolichol-linked oligosaccharide biosynthesis enzyme, putative | Yes |
| 375 | PBANKA_040560 | EB1 homolog, putative | Yes |
| 376 | PBANKA_081420 | elongation factor 1-beta, putative | Yes |
| 377 | PBANKA_010120 | elongation factor G, putative (EF-G) | Yes |
| 378 | PBANKA_134560 | elongation factor Tu, putative | Yes |
| 379 | PBANKA_135080 | endopeptidase, putative | No |
| 380 | PBANKA_101410 | exodeoxyribonuclease III, putative | Yes |
| 381 | PBANKA_070650 | exonuclease, putative | Yes |
| 382 | PBANKA_141440 | exportin-T, putative | Yes |
| 383 | PBANKA_142230 | FAD-dependent monooxygenase, putative | Yes |
| 384 | PBANKA_020430 | fatty acid elongation protein, GNS1/SUR4 family, putative, pseudogene | Yes |
| 385 | PBANKA_061790 | ferrodoxin reductase-like protein, putative | Yes |
| 386 | PBANKA_146040 | FK506-binding protein (FKBP)-type peptidyl-prolyl isomerase, putative (FKBP35) | Yes |
| 387 | PBANKA_050430 | flagellar outer arm dynein-associated protein, putative | No |
| 388 | PBANKA_082410 | flavodoxin-like protein | Yes |
| 389 | PBANKA_131270 | gamete egress and sporozoite traversal protein (GEST) | Yes |
| 390 | PBANKA_010130 | geranylgeranyltransferase, putative | Yes |
| 391 | PBANKA_133890 | glideosome associated protein with multiple membrane spans 1, putative (GAPM1) | Yes |
| 392 | PBANKA_111530 | glideosome-associated protein 40, putative (GAP40) | No |
| 393 | PBANKA_143760 | glideosome-associated protein 45, putative | Yes |
| 394 | PBANKA_136200 | glutamate - tRNA ligase, putative | Yes |
| 395 | PBANKA_102340 | glutathione reductase, putative (GR) | Yes |
| 396 | PBANKA_111180 | glutathione synthetase (GS) | Yes |
| 397 | PBANKA_041040 | glycogen synthase kinase 3 (GSK3) | Yes |
| 398 | PBANKA_122430 | glycosyltransferase, putative | Yes |
| 399 | PBANKA_134570 | GPI transamidase subunit PIG-U, putative | Yes |
| 400 | PBANKA_070190 | GPI-anchored micronemal antigen, putative (GAMA) | Yes |
| 401 | PBANKA_071370 | GTPase, putative | No |
| 402 | PBANKA_093030 | GTP-binding nuclear protein, putative | Yes |
| 403 | PBANKA_031000 | heat shock 40 kDa protein, putative | Yes |
| 404 | PBANKA_060960 | heat shock protein 20, putative | Yes |
| 405 | PBANKA_081890 | heat shock protein 70, putative | Yes |
| 406 | PBANKA_121910 | heat shock protein 90, putative | Yes |
| 407 | PBANKA_071190 | heat shock protein, putative (HSP70) | Yes |
| 408 | PBANKA_111380 | helicase, putative (UIS26) | Yes |
| 409 | PBANKA_030250 | hexose transporter (HT) | Yes |
| 410 | PBANKA_083480 | histidyl-tRNA synthetase, putative | Yes |
| 411 | PBANKA_082650 | histone deacetylase, putative (HDAC1) | Yes |
| 412 | PBANKA_094180 | histone H2B, putative (H2B) | Yes |
| 413 | PBANKA_134840 | histone H3 variant, putative (CenH3) | Yes |
| 414 | PBANKA_010880 | histone H3, putative | No |
| 415 | PBANKA_020340 | human hepatopoietin-like protein, putative | Yes |
| 416 | PBANKA_090710 | inner membrane complex protein 1b (IMC1b) | Yes |
| 417 | PBANKA_041410 | inorganic pyrophosphatase, putative | Yes |
| 418 | PBANKA_113080 | inositol-polyphosphate 5-phosphatase, putative | Yes |
| 419 | PBANKA_134770 | isoleucine - tRNA ligase, putative | Yes |
| 420 | PBANKA_060950 | kinesin-related protein, putative | Yes |
| 421 | PBANKA_080770 | kinesin-related protein, putative | Yes |
| 422 | PBANKA_103520 | LCCL domain-containing protein (CCp3) | Yes |
| 423 | PBANKA_071400 | meiotic recombination protein DMC1-like protein, putative | Yes |
| 424 | PBANKA_070710 | membrane skeletal protein, putative | Yes |
| 425 | PBANKA_120200 | membrane skeletal protein, putative | Yes |
| 426 | PBANKA_124060 | membrane skeletal protein, putative | Yes |
| 427 | PBANKA_111960 | merozoite surface protein 10, putative (MSP10) | Yes |
| 428 | PBANKA_081530 | met-10 like protein, putative | Yes |
| 429 | PBANKA_123130 | metabolite/drug transporter, putative | Yes |
| 430 | PBANKA_113140 | metacaspase 1 (MCA1) | Yes |
| 431 | PBANKA_122150 | mitochondrial ribosomal protein L1 precursor, putative | Yes |
| 432 | PBANKA_041330 | mitochondrial ribosomal protein L29/L47 precursor, putative | Yes |
| 433 | PBANKA_083380 | mRNA processing protein, putative | Yes |
| 434 | PBANKA_123350 | mRNA-binding protein PUF1 (PUF1) | Yes |
| 435 | PBANKA_133960 | MSF1-like protein, putative | Yes |
| 436 | PBANKA_145950 | myosin light chain 1, putative,myosin A tail domain interacting protein MTIP, putative (MTIP) | Yes |
| 437 | PBANKA_114340 | NADH-cytochrome b5 reductase, putative | Yes |
| 438 | PBANKA_111570 | Ndc80 homolog, putative | No |
| 439 | PBANKA_114240 | nucleoside diphosphate kinase b, putative | No |
| 440 | PBANKA_134720 | nucleotidyltransferase, putative | Yes |
| 441 | PBANKA_121810 | oocyst capsule protein (Cap380) | Yes |
| 442 | PBANKA_133070 | organelle ribosomal protein L22/L17 precursor, putative | No |
| 443 | PBANKA_031250 | origin recognition complex subunit 5, putative (ORC5) | Yes |
| 444 | PBANKA_010740 | ornithine aminotransferase, putative (OAT) | Yes |
| 445 | PBANKA_050740 | orotidine-monophosphate-decarboxylase, putative (OMPDC) | Yes |
| 446 | PBANKA_146300 | osmiophilic body protein (G377) | Yes |
| 447 | PBANKA_082400 | OTU-like cysteine protease, putative | Yes |
| 448 | PBANKA_080940 | P1 nuclease, putative | Yes |
| 449 | PBANKA_061140 | pantothenate kinase, putative | Yes |
| 450 | PBANKA_102260 | pantothenate kinase, putative | Yes |
| 451 | PBANKA_061960 | PelOta protein homologue, putative | Yes |
| 452 | PBANKA_100650 | peptidase, M22 family, putative | Yes |
| 453 | PBANKA_092850 | peptidase, putative | Yes |
| 454 | PBANKA_080930 | peptide deformylase, putative | Yes |
| 455 | PBANKA_122720 | peptidyl-prolyl cis-trans isomerase, putative (CYP81) | No |
| 456 | PBANKA_082420 | perforin like protein 3 (PPLP3) | Yes |
| 457 | PBANKA_071140 | perforin like protein 4 (PPLP4) | Yes |
| 458 | PBANKA_071160 | perforin like protein 5 (PPLP5) | Yes |
| 459 | PBANKA_010770 | permease, putative | Yes |
| 460 | PBANKA_092650 | petidase, M16 family, putative | No |
| 461 | PBANKA_101790 | phosphoenolpyruvate carboxylase (PEPC) | No |
| 462 | PBANKA_020460 | photosensitized INA-labeled protein 1, putative | Yes |
| 463 | PBANKA_040970 | plasmepsin VI | No |
| 464 | PBANKA_040220 | plasmoredoxin (Plrx) | No |
| 465 | PBANKA_061030 | polyubiquitin, putative | Yes |
| 466 | PBANKA_110830 | Pre-mRNA-processing ATP-dependent RNA helicase prp5, putative (PRP5) | Yes |
| 467 | PBANKA_122650 | proteasome beta-subunit, putative | Yes |
| 468 | PBANKA_080820 | proteasome component C8, putative | Yes |
| 469 | PBANKA_122290 | proteasome subunit alpha type 5, putative | No |
| 470 | PBANKA_135090 | protein kinase 6 (PK6) | No |
| 471 | PBANKA_110320 | protein kinase, putative | Yes |
| 472 | PBANKA_134590 | protein kinase, putative | Yes |
| 473 | PBANKA_082620 | protein phosphatase-beta, putative | No |
| 474 | PBANKA_031100 | protein transport protein Sec31, putative (SEC31) | Yes |
| 475 | PBANKA_123080 | pyridoxal kinase-like protein, putative | Yes |
| 476 | PBANKA_134200 | Qa-SNARE protein, putative | Yes |
| 477 | PBANKA_070860 | quinone oxidoreductase, putative | No |
| 478 | PBANKA_111350 | Rab1a, putative | Yes |
| 479 | PBANKA_051380 | RAP protein, putative | Yes |
| 480 | PBANKA_144130 | RAP protein, putative | Yes |
| 481 | PBANKA_020250 | replication factor c protein, putative | Yes |
| 482 | PBANKA_031470 | replication factor C, subunit 2, putative | No |
| 483 | PBANKA_123610 | ribosomal large subunit pseudouridylate synthase, putative | Yes |
| 484 | PBANKA_030570 | ribosome-recycling factor, putative (RRF) | Yes |
| 485 | PBANKA_050570 | RNA helicase, putative | Yes |
| 486 | PBANKA_010390 | RNA-binding protein, putative | No |
| 487 | PBANKA_103620 | RNA-binding protein, putative | No |
| 488 | PBANKA_103630 | RNA-binding protein, putative | Yes |
| 489 | PBANKA_050460 | S-adenosyl-L-methionine-dependent methyltransferase, putative | Yes |
| 490 | PBANKA_103780 | secreted ookinete adhesive protein (SOAP) | Yes |
| 491 | PBANKA_061920 | secreted ookinete protein, putative (PSOP1) | Yes |
| 492 | PBANKA_111340 | secreted ookinete protein, putative (PSOP12) | Yes |
| 493 | PBANKA_114370 | secreted ookinete protein, putative (PSOP2) | Yes |
| 494 | PBANKA_112900 | secreted ookinete protein, putative (PSOP6) | Yes |
| 495 | PBANKA_135340 | secreted ookinete protein, putative (PSOP7) | Yes |
| 496 | PBANKA_020350 | selenocysteine-specific elongation factor selB homologue, putative | Yes |
| 497 | PBANKA_082960 | serine/threonine protein kinase, putative | Yes |
| 498 | PBANKA_135260 | serine/threonine protein kinase, putative | No |
| 499 | PBANKA_146050 | serine/threonine protein kinase, putative | Yes |
| 500 | PBANKA_110770 | SET domain protein, putative | Yes |
| 501 | PBANKA_134620 | signal peptidase 21 kDa subunit, putative (SP21) | Yes |
| 502 | PBANKA_071430 | small heat shock protein HSP20 (HSP20) | Yes |
| 503 | PBANKA_092280 | small nuclear ribonucleoprotein D1, putative (SNRPD1) | Yes |
| 504 | PBANKA_070930 | small ribosomal subunit processing microtubule-associated protein, putative | Yes |
| 505 | PBANKA_131230 | SNARE protein, putative | Yes |
| 506 | PBANKA_081070 | subpellicular microtubule protein 1, putative (SPM1) | Yes |
| 507 | PBANKA_144500 | subpellicular microtubule protein 2, putative (SPM2) | Yes |
| 508 | PBANKA_110710 | subtilisin-like protease 1, putative (SUB1) | Yes |
| 509 | PBANKA_011200 | syntaxin binding protein, putative | Yes |
| 510 | PBANKA_094280 | syntaxin, putative | Yes |
| 511 | PBANKA_020180 | TatD-like deoxyribonuclease, putative | Yes |
| 512 | PBANKA_136130 | TBC domain protein, putative | No |
| 513 | PBANKA_082570 | telomeric repeat binding factor 1, putative | Yes |
| 514 | PBANKA_145000 | tetQ family GTPase, putative | No |
| 515 | PBANKA_082520 | thiamin pyrophosphokinase, putative | Yes |
| 516 | PBANKA_092220 | ThiF family protein, putative | Yes |
| 517 | PBANKA_082020 | thioredoxin, putative | Yes |
| 518 | PBANKA_083520 | transcription factor with AP2 domain(s), putative | Yes |
| 519 | PBANKA_052170 | transcription factor with AP2 domain, putative (ApiAP2) | Yes |
| 520 | PBANKA_051400 | transcription factor, putative | Yes |
| 521 | PBANKA_134380 | transcriptional regulatory protein sir2a (Sir2A) | Yes |
| 522 | PBANKA_134760 | translation initiation factor 6, putative | No |
| 523 | PBANKA_050400 | tRNA methyltransferase, putative | Yes |
| 524 | PBANKA_111370 | tubulin-tyrosine ligase, putative | Yes |
| 525 | PBANKA_122280 | tyrosyl-tRNA synthetase, putative | No |
| 526 | PBANKA_092880 | U2 snRNP auxiliary factor, small subunit, putative | Yes |
| 527 | PBANKA_093780 | U3/U14 snoRNA-associated small subunit rRNA processing protein, putative | Yes |
| 528 | PBANKA_121480 | U4/U6.U5 tri-snRNP-associated protein 1, putative (SART1) | Yes |
| 529 | PBANKA_134020 | U6 snRNA-associated Sm-like protein LSm6, putative (LSM6) | Yes |
| 530 | PBANKA_090450 | ubiquitin activating enzyme (E1) subunit Aos1, putative | Yes |
| 531 | PBANKA_060280 | ubiquitin-conjugating enzyme e2, putative | Yes |
| 532 | PBANKA_110490 | UDP-N-acetyl glucosamine:UMP antiporter, putative | Yes |
| 533 | PBANKA_123400 | vacuolar ATP synthetase, putative | Yes |
| 534 | PBANKA_031310 | vacuolar sorting protein VPS45, putative | Yes |
| 535 | PBANKA_122890 | von willebrand factor a-domain-related protein (WARP) | Yes |
| 536 | PBANKA_020970 | zinc carboxy peptidase, putative | Yes |
| 537 | PBANKA_060860 | zinc finger protein, putative | No |
| 538 | PBANKA_083330 | zinc finger protein, putative | Yes |
| 539 | PBANKA_113490 | zinc finger protein, putative | Yes |
| 540 | PBANKA_123270 | zinc finger protein, putative | Yes |
| 541 | PBANKA_120260 | zinc finger, C3HC4 type, putative | No |

^a^In experiment 2, ChIP-seq was performed in the Solid platform. Mapping of reads was performed in a lifescope program equipped with the system in the default conditions. The mapped reads were filtered using an in-house program under the condition of 60 bp with no mismatch. Peak-calling was performed using MACS2 using IP and input data under the condition of FDR < 0.01 and fold enrichment over input > 5, and 1,111 peaks were identified within the *P. berghei* genome. Target genes of AP2-O were identified from the summits of the peaks using in-house programs. In brief, the binding motif nearest to the summit was identified as a putative binding site of AP2-O in each summit. Genes were identified as AP2-O targets when their 1.2-kbp upstream regions contained the predicted binding sites of AP2-O. Only the binding sites in intergenic regions were used for target prediction. Therefore, when the intergenic region was less than 1.2-kbp, the entire intergenic region was used for target prediction. Gene ID and functional annotation were attributed to each gene according to those in PlasmoDB ver.12.0. Target genes were sorted in alphabetical order of attributed functional annotations. Target genes that are identical to those identified in experiment 1 are indicated by “Yes” in the last column.

**Table S2. Target genes of AP2-O identified in experiment 1**^a^

|  | Gene ID | Functional annotation | In experiment 2 |
| --- | --- | --- | --- |
| 1 | PBANKA_113810 | 14-3-3 protein, putative | Yes |
| 2 | PBANKA_040310 | 1-cys-glutaredoxin-like protein-1, putative (GLP1) | Yes |
| 3 | PBANKA_134780 | 20 kDa chaperonin, putative (CPN20) | Yes |
| 4 | PBANKA_051500 | 25 kDa ookinete surface antigen precursor (P25) | Yes |
| 5 | PBANKA_051490 | 28 kDa ookinete surface protein (P28) | Yes |
| 6 | PBANKA_061750 | 3'-5' exoribonuclease Csl4 homolog, putative | Yes |
| 7 | PBANKA_110420 | 3-methyl-2-oxobutanoate dehydrogenase (lipoamide), putative | Yes |
| 8 | PBANKA_112510 | 3-oxoacyl-acyl-carrier protein synthase, putative (FabB/FabF) | No |
| 9 | PBANKA_131080 | 40S ribosomal protein S2, putative | Yes |
| 10 | PBANKA_071780 | 60S ribosomal protein L15, putative | Yes |
| 11 | PBANKA_110760 | 6-cysteine protein (P38) | Yes |
| 12 | PBANKA_110310 | actin-depolymerizing factor 1 (ADF1) | Yes |
| 13 | PBANKA_061680 | actin-related protein | Yes |
| 14 | PBANKA_020930 | actin-related protein (ARP1) | Yes |
| 15 | PBANKA_111870 | acyl-CoA synthetase, putative | Yes |
| 16 | PBANKA_092910 | acyl-CoA-binding protein, putative | Yes |
| 17 | PBANKA_071760 | adenylate kinase 1, putative | Yes |
| 18 | PBANKA_113110 | adenylosuccinate synthetase, putative | Yes |
| 19 | PBANKA_124100 | ADP-ribosylation factor GTPase-activating protein, putative | No |
| 20 | PBANKA_090600 | alpha/beta hydrolase, putative | Yes |
| 21 | PBANKA_112340 | anaphase-promoting complex subunit, putative | Yes |
| 22 | PBANKA_091500 | apical membrane antigen 1 (AMA1) | No |
| 23 | PBANKA_070320 | apicoplast ribosomal protein L21 precursor, putative | Yes |
| 24 | PBANKA_134740 | apurinic/apyrimidinic endonuclease, putative | No |
| 25 | PBANKA_021020 | aspartyl-tRNA synthetase, putative | Yes |
| 26 | PBANKA_132810 | ATP synthase (C/AC39) subunit, putative | No |
| 27 | PBANKA_145030 | ATP synthase subunit beta, mitochondrial, putative | Yes |
| 28 | PBANKA_112260 | ATP-dependent DEAD box helicase, putative | Yes |
| 29 | PBANKA_101480 | ATP-dependent DNA helicase, putative | Yes |
| 30 | PBANKA_101320 | ATP-specific succinyl-CoA synthetase beta subunit, putative | Yes |
| 31 | PBANKA_133820 | beta-hydroxyacyl-ACP dehydratase, putative (FabZ) | Yes |
| 32 | PBANKA_051100 | biotin--acetyl-CoA-carboxylase, putative | Yes |
| 33 | PBANKA_103640 | BOP1-like protein, putative | Yes |
| 34 | PBANKA_081590 | BSD domain, putative | No |
| 35 | PBANKA_122640 | cactin homolog, putative | No |
| 36 | PBANKA_031420 | calcium dependent protein kinase 1 (CDPK1) | Yes |
| 37 | PBANKA_040820 | calcium dependent protein kinase 3 (CDPK3) | Yes |
| 38 | PBANKA_010420 | calcium-binding protein, putative | Yes |
| 39 | PBANKA_123430 | carbon catabolite repressor protein 4, putative (CCR4) | Yes |
| 40 | PBANKA_113320 | cdc2-related kinase 2 (CRK2) | Yes |
| 41 | PBANKA_071870 | CDGSH iron-sulfur domain-containing protein, putative | Yes |
| 42 | PBANKA_111190 | CDK-activating kinase assembly factor, putative (MAT1) | Yes |
| 43 | PBANKA_143230 | cell traversal protein for ookinetes and sporozoites (CelTOS) | Yes |
| 44 | PBANKA_020630 | centrin, putative | No |
| 45 | PBANKA_051180 | centrin-3, putative | No |
| 46 | PBANKA_080050 | chitinase (CHT1) | Yes |
| 47 | PBANKA_112190 | chorismate synthase, putative (CS) | Yes |
| 48 | PBANKA_041290 | circumsporozoite- and TRAP-related protein (CTRP) | Yes |
| 49 | PBANKA_031400 | clathrin coat assembly protein, putative | Yes |
| 50 | PBANKA_070920 | clp1-related protein, putative | Yes |
| 51 | PBANKA_122230 | coatomer epsilon subunit, putative | Yes |
| 52 | PBANKA_020370 | conserved Plasmodium membrane protein, unknown function | Yes |
| 53 | PBANKA_092250 | conserved Plasmodium protein, unknown function | Yes |
| 54 | PBANKA_110990 | conserved Plasmodium protein, unknown function | Yes |
| 55 | PBANKA_111000 | conserved Plasmodium protein, unknown function | Yes |
| 56 | PBANKA_092240 | conserved Plasmodium protein, unknown function | Yes |
| 57 | PBANKA_110910 | conserved Plasmodium protein, unknown function | Yes |
| 58 | PBANKA_083470 | conserved Plasmodium protein, unknown function | Yes |
| 59 | PBANKA_111640 | conserved Plasmodium protein, unknown function | Yes |
| 60 | PBANKA_093010 | conserved Plasmodium protein, unknown function | Yes |
| 61 | PBANKA_111610 | conserved Plasmodium protein, unknown function | Yes |
| 62 | PBANKA_082150 | conserved Plasmodium protein, unknown function | Yes |
| 63 | PBANKA_102760 | conserved Plasmodium protein, unknown function | No |
| 64 | PBANKA_111760 | conserved Plasmodium protein, unknown function | Yes |
| 65 | PBANKA_134730 | conserved Plasmodium protein, unknown function | Yes |
| 66 | PBANKA_092800 | conserved Plasmodium protein, unknown function | Yes |
| 67 | PBANKA_120345 | conserved Plasmodium protein, unknown function | No |
| 68 | PBANKA_051810 | conserved Plasmodium protein, unknown function | Yes |
| 69 | PBANKA_082120 | conserved Plasmodium protein, unknown function | Yes |
| 70 | PBANKA_082130 | conserved Plasmodium protein, unknown function | Yes |
| 71 | PBANKA_041065 | conserved Plasmodium protein, unknown function | Yes |
| 72 | PBANKA_136440 | conserved Plasmodium protein, unknown function | Yes |
| 73 | PBANKA_136360 | conserved Plasmodium protein, unknown function | Yes |
| 74 | PBANKA_123600 | conserved Plasmodium protein, unknown function | Yes |
| 75 | PBANKA_133930 | conserved Plasmodium protein, unknown function | Yes |
| 76 | PBANKA_135250 | conserved Plasmodium protein, unknown function | Yes |
| 77 | PBANKA_122140 | conserved Plasmodium protein, unknown function | Yes |
| 78 | PBANKA_133810 | conserved Plasmodium protein, unknown function | No |
| 79 | PBANKA_131130 | conserved Plasmodium protein, unknown function | Yes |
| 80 | PBANKA_131240 | conserved Plasmodium protein, unknown function | Yes |
| 81 | PBANKA_131310 | conserved Plasmodium protein, unknown function | Yes |
| 82 | PBANKA_060340 | conserved Plasmodium protein, unknown function | Yes |
| 83 | PBANKA_060350 | conserved Plasmodium protein, unknown function | Yes |
| 84 | PBANKA_061300 | conserved Plasmodium protein, unknown function | Yes |
| 85 | PBANKA_133660 | conserved Plasmodium protein, unknown function | Yes |
| 86 | PBANKA_120450 | conserved Plasmodium protein, unknown function | Yes |
| 87 | PBANKA_134580 | conserved Plasmodium protein, unknown function | No |
| 88 | PBANKA_134550 | conserved Plasmodium protein, unknown function | Yes |
| 89 | PBANKA_020850 | conserved Plasmodium protein, unknown function | Yes |
| 90 | PBANKA_020860 | conserved Plasmodium protein, unknown function | Yes |
| 91 | PBANKA_050440 | conserved Plasmodium protein, unknown function | Yes |
| 92 | PBANKA_050470 | conserved Plasmodium protein, unknown function | Yes |
| 93 | PBANKA_050520 | conserved Plasmodium protein, unknown function | Yes |
| 94 | PBANKA_081480 | conserved Plasmodium protein, unknown function | Yes |
| 95 | PBANKA_081020 | conserved Plasmodium protein, unknown function | Yes |
| 96 | PBANKA_081030 | conserved Plasmodium protein, unknown function | Yes |
| 97 | PBANKA_081430 | conserved Plasmodium protein, unknown function | No |
| 98 | PBANKA_111840 | conserved Plasmodium protein, unknown function | Yes |
| 99 | PBANKA_111880 | conserved Plasmodium protein, unknown function | Yes |
| 100 | PBANKA_103890 | conserved Plasmodium protein, unknown function | Yes |
| 101 | PBANKA_111920 | conserved Plasmodium protein, unknown function | Yes |
| 102 | PBANKA_122910 | conserved Plasmodium protein, unknown function | Yes |
| 103 | PBANKA_122780 | conserved Plasmodium protein, unknown function | Yes |
| 104 | PBANKA_122790 | conserved Plasmodium protein, unknown function | Yes |
| 105 | PBANKA_122830 | conserved Plasmodium protein, unknown function | Yes |
| 106 | PBANKA_071910 | conserved Plasmodium protein, unknown function | Yes |
| 107 | PBANKA_123440 | conserved Plasmodium protein, unknown function | Yes |
| 108 | PBANKA_134130 | conserved Plasmodium protein, unknown function | No |
| 109 | PBANKA_123460 | conserved Plasmodium protein, unknown function | Yes |
| 110 | PBANKA_061640 | conserved Plasmodium protein, unknown function | Yes |
| 111 | PBANKA_061650 | conserved Plasmodium protein, unknown function | Yes |
| 112 | PBANKA_140440 | conserved Plasmodium protein, unknown function | Yes |
| 113 | PBANKA_140820 | conserved Plasmodium protein, unknown function | Yes |
| 114 | PBANKA_140920 | conserved Plasmodium protein, unknown function | Yes |
| 115 | PBANKA_112250 | conserved Plasmodium protein, unknown function | Yes |
| 116 | PBANKA_131860 | conserved Plasmodium protein, unknown function | Yes |
| 117 | PBANKA_112950 | conserved Plasmodium protein, unknown function | Yes |
| 118 | PBANKA_134510 | conserved Plasmodium protein, unknown function | No |
| 119 | PBANKA_134480 | conserved Plasmodium protein, unknown function | No |
| 120 | PBANKA_010660 | conserved Plasmodium protein, unknown function | No |
| 121 | PBANKA_010670 | conserved Plasmodium protein, unknown function | No |
| 122 | PBANKA_010640 | conserved Plasmodium protein, unknown function | Yes |
| 123 | PBANKA_010650 | conserved Plasmodium protein, unknown function | Yes |
| 124 | PBANKA_050720 | conserved Plasmodium protein, unknown function | Yes |
| 125 | PBANKA_050820 | conserved Plasmodium protein, unknown function | Yes |
| 126 | PBANKA_120970 | conserved Plasmodium protein, unknown function | Yes |
| 127 | PBANKA_040580 | conserved Plasmodium protein, unknown function | Yes |
| 128 | PBANKA_040590 | conserved Plasmodium protein, unknown function | Yes |
| 129 | PBANKA_062060 | conserved Plasmodium protein, unknown function | Yes |
| 130 | PBANKA_062070 | conserved Plasmodium protein, unknown function | Yes |
| 131 | PBANKA_120940 | conserved Plasmodium protein, unknown function | Yes |
| 132 | PBANKA_062090 | conserved Plasmodium protein, unknown function | Yes |
| 133 | PBANKA_062100 | conserved Plasmodium protein, unknown function | Yes |
| 134 | PBANKA_062110 | conserved Plasmodium protein, unknown function | Yes |
| 135 | PBANKA_062150 | conserved Plasmodium protein, unknown function | Yes |
| 136 | PBANKA_101920 | conserved Plasmodium protein, unknown function | Yes |
| 137 | PBANKA_101900 | conserved Plasmodium protein, unknown function | Yes |
| 138 | PBANKA_101935 | conserved Plasmodium protein, unknown function | Yes |
| 139 | PBANKA_100640 | conserved Plasmodium protein, unknown function | Yes |
| 140 | PBANKA_050610 | conserved Plasmodium protein, unknown function | No |
| 141 | PBANKA_040170 | conserved Plasmodium protein, unknown function | Yes |
| 142 | PBANKA_051070 | conserved Plasmodium protein, unknown function | Yes |
| 143 | PBANKA_021110 | conserved Plasmodium protein, unknown function | Yes |
| 144 | PBANKA_021030 | conserved Plasmodium protein, unknown function | Yes |
| 145 | PBANKA_011160 | conserved Plasmodium protein, unknown function | Yes |
| 146 | PBANKA_071680 | conserved Plasmodium protein, unknown function | Yes |
| 147 | PBANKA_091540 | conserved Plasmodium protein, unknown function | Yes |
| 148 | PBANKA_101580 | conserved Plasmodium protein, unknown function | Yes |
| 149 | PBANKA_144460 | conserved Plasmodium protein, unknown function | Yes |
| 150 | PBANKA_144470 | conserved Plasmodium protein, unknown function | Yes |
| 151 | PBANKA_093660 | conserved Plasmodium protein, unknown function | Yes |
| 152 | PBANKA_093670 | conserved Plasmodium protein, unknown function | Yes |
| 153 | PBANKA_102380 | conserved Plasmodium protein, unknown function | Yes |
| 154 | PBANKA_083450 | conserved Plasmodium protein, unknown function | No |
| 155 | PBANKA_060490 | conserved Plasmodium protein, unknown function | No |
| 156 | PBANKA_083370 | conserved Plasmodium protein, unknown function | Yes |
| 157 | PBANKA_134850 | conserved Plasmodium protein, unknown function | Yes |
| 158 | PBANKA_111200 | conserved Plasmodium protein, unknown function | Yes |
| 159 | PBANKA_081840 | conserved Plasmodium protein, unknown function | Yes |
| 160 | PBANKA_081690 | conserved Plasmodium protein, unknown function | Yes |
| 161 | PBANKA_081650 | conserved Plasmodium protein, unknown function | Yes |
| 162 | PBANKA_081620 | conserved Plasmodium protein, unknown function | Yes |
| 163 | PBANKA_090620 | conserved Plasmodium protein, unknown function | No |
| 164 | PBANKA_090630 | conserved Plasmodium protein, unknown function | No |
| 165 | PBANKA_090620 | conserved Plasmodium protein, unknown function | No |
| 166 | PBANKA_090630 | conserved Plasmodium protein, unknown function | No |
| 167 | PBANKA_090610 | conserved Plasmodium protein, unknown function | Yes |
| 168 | PBANKA_122540 | conserved Plasmodium protein, unknown function | Yes |
| 169 | PBANKA_135460 | conserved Plasmodium protein, unknown function | Yes |
| 170 | PBANKA_135950 | conserved Plasmodium protein, unknown function | Yes |
| 171 | PBANKA_124010 | conserved Plasmodium protein, unknown function | Yes |
| 172 | PBANKA_134870 | conserved Plasmodium protein, unknown function | Yes |
| 173 | PBANKA_143680 | conserved Plasmodium protein, unknown function | No |
| 174 | PBANKA_143520 | conserved Plasmodium protein, unknown function | Yes |
| 175 | PBANKA_143770 | conserved Plasmodium protein, unknown function | Yes |
| 176 | PBANKA_143780 | conserved Plasmodium protein, unknown function | Yes |
| 177 | PBANKA_142430 | conserved Plasmodium protein, unknown function | Yes |
| 178 | PBANKA_145120 | conserved Plasmodium protein, unknown function | Yes |
| 179 | PBANKA_145150 | conserved Plasmodium protein, unknown function | Yes |
| 180 | PBANKA_145080 | conserved Plasmodium protein, unknown function | Yes |
| 181 | PBANKA_091670 | conserved Plasmodium protein, unknown function | Yes |
| 182 | PBANKA_110750 | conserved Plasmodium protein, unknown function | Yes |
| 183 | PBANKA_090320 | conserved Plasmodium protein, unknown function | Yes |
| 184 | PBANKA_135170 | conserved Plasmodium protein, unknown function | Yes |
| 185 | PBANKA_135230 | conserved Plasmodium protein, unknown function | Yes |
| 186 | PBANKA_135240 | conserved Plasmodium protein, unknown function | Yes |
| 187 | PBANKA_060400 | conserved Plasmodium protein, unknown function | Yes |
| 188 | PBANKA_061240 | conserved Plasmodium protein, unknown function | Yes |
| 189 | PBANKA_062230 | conserved Plasmodium protein, unknown function | Yes |
| 190 | PBANKA_102100 | conserved Plasmodium protein, unknown function | Yes |
| 191 | PBANKA_090520 | conserved Plasmodium protein, unknown function | No |
| 192 | PBANKA_041720 | conserved Plasmodium protein, unknown function | Yes |
| 193 | PBANKA_091690 | conserved Plasmodium protein, unknown function | Yes |
| 194 | PBANKA_071450 | conserved Plasmodium protein, unknown function | Yes |
| 195 | PBANKA_091630 | conserved Plasmodium protein, unknown function | No |
| 196 | PBANKA_091665 | conserved Plasmodium protein, unknown function | No |
| 197 | PBANKA_091650 | conserved Plasmodium protein, unknown function | Yes |
| 198 | PBANKA_083590 | conserved Plasmodium protein, unknown function | Yes |
| 199 | PBANKA_091770 | conserved Plasmodium protein, unknown function | Yes |
| 200 | PBANKA_143900 | conserved Plasmodium protein, unknown function | No |
| 201 | PBANKA_093110 | conserved Plasmodium protein, unknown function | Yes |
| 202 | PBANKA_060250 | conserved Plasmodium protein, unknown function | No |
| 203 | PBANKA_103450 | conserved Plasmodium protein, unknown function | No |
| 204 | PBANKA_060170 | conserved Plasmodium protein, unknown function | No |
| 205 | PBANKA_092560 | conserved Plasmodium protein, unknown function | Yes |
| 206 | PBANKA_092450 | conserved Plasmodium protein, unknown function | Yes |
| 207 | PBANKA_092400 | conserved Plasmodium protein, unknown function | Yes |
| 208 | PBANKA_101820 | conserved Plasmodium protein, unknown function | No |
| 209 | PBANKA_072090 | conserved Plasmodium protein, unknown function | Yes |
| 210 | PBANKA_072100 | conserved Plasmodium protein, unknown function | Yes |
| 211 | PBANKA_122080 | conserved Plasmodium protein, unknown function | Yes |
| 212 | PBANKA_136270 | conserved Plasmodium protein, unknown function | Yes |
| 213 | PBANKA_091600 | conserved Plasmodium protein, unknown function | Yes |
| 214 | PBANKA_142280 | conserved Plasmodium protein, unknown function | Yes |
| 215 | PBANKA_142290 | conserved Plasmodium protein, unknown function | Yes |
| 216 | PBANKA_122610 | conserved Plasmodium protein, unknown function | Yes |
| 217 | PBANKA_135380 | conserved Plasmodium protein, unknown function | Yes |
| 218 | PBANKA_135310 | conserved Plasmodium protein, unknown function | Yes |
| 219 | PBANKA_061150 | conserved Plasmodium protein, unknown function | Yes |
| 220 | PBANKA_051530 | conserved Plasmodium protein, unknown function | Yes |
| 221 | PBANKA_061120 | conserved Plasmodium protein, unknown function | Yes |
| 222 | PBANKA_123280 | conserved Plasmodium protein, unknown function | Yes |
| 223 | PBANKA_123230 | conserved Plasmodium protein, unknown function | No |
| 224 | PBANKA_130980 | conserved Plasmodium protein, unknown function | No |
| 225 | PBANKA_112310 | conserved Plasmodium protein, unknown function | Yes |
| 226 | PBANKA_112320 | conserved Plasmodium protein, unknown function | Yes |
| 227 | PBANKA_112330 | conserved Plasmodium protein, unknown function | Yes |
| 228 | PBANKA_112460 | conserved Plasmodium protein, unknown function | Yes |
| 229 | PBANKA_112470 | conserved Plasmodium protein, unknown function | Yes |
| 230 | PBANKA_133050 | conserved Plasmodium protein, unknown function | No |
| 231 | PBANKA_061210 | conserved Plasmodium protein, unknown function | Yes |
| 232 | PBANKA_050530 | conserved Plasmodium protein, unknown function | Yes |
| 233 | PBANKA_050540 | conserved Plasmodium protein, unknown function | Yes |
| 234 | PBANKA_094160 | conserved Plasmodium protein, unknown function | No |
| 235 | PBANKA_040900 | conserved Plasmodium protein, unknown function | Yes |
| 236 | PBANKA_146120 | conserved Plasmodium protein, unknown function | Yes |
| 237 | PBANKA_146130 | conserved Plasmodium protein, unknown function | Yes |
| 238 | PBANKA_120460 | conserved Plasmodium protein, unknown function | Yes |
| 239 | PBANKA_120470 | conserved Plasmodium protein, unknown function | Yes |
| 240 | PBANKA_041350 | conserved Plasmodium protein, unknown function | No |
| 241 | PBANKA_020680 | conserved Plasmodium protein, unknown function | Yes |
| 242 | PBANKA_122270 | conserved Plasmodium protein, unknown function | Yes |
| 243 | PBANKA_146100 | conserved Plasmodium protein, unknown function | Yes |
| 244 | PBANKA_030580 | conserved Plasmodium protein, unknown function | No |
| 245 | PBANKA_030590 | conserved Plasmodium protein, unknown function | No |
| 246 | PBANKA_050860 | conserved Plasmodium protein, unknown function | Yes |
| 247 | PBANKA_050870 | conserved Plasmodium protein, unknown function | Yes |
| 248 | PBANKA_050880 | conserved Plasmodium protein, unknown function | Yes |
| 249 | PBANKA_050890 | conserved Plasmodium protein, unknown function | Yes |
| 250 | PBANKA_071110 | conserved Plasmodium protein, unknown function | Yes |
| 251 | PBANKA_031280 | conserved Plasmodium protein, unknown function | No |
| 252 | PBANKA_102120 | conserved Plasmodium protein, unknown function | Yes |
| 253 | PBANKA_091710 | conserved Plasmodium protein, unknown function | Yes |
| 254 | PBANKA_091730 | conserved Plasmodium protein, unknown function | No |
| 255 | PBANKA_101470 | conserved Plasmodium protein, unknown function | Yes |
| 256 | PBANKA_083080 | conserved Plasmodium protein, unknown function | Yes |
| 257 | PBANKA_082950 | conserved Plasmodium protein, unknown function | Yes |
| 258 | PBANKA_082990 | conserved Plasmodium protein, unknown function | Yes |
| 259 | PBANKA_083040 | conserved Plasmodium protein, unknown function | Yes |
| 260 | PBANKA_083280 | conserved Plasmodium protein, unknown function | Yes |
| 261 | PBANKA_092160 | conserved Plasmodium protein, unknown function | Yes |
| 262 | PBANKA_134700 | conserved Plasmodium protein, unknown function | Yes |
| 263 | PBANKA_110590 | conserved Plasmodium protein, unknown function | Yes |
| 264 | PBANKA_110690 | conserved Plasmodium protein, unknown function | Yes |
| 265 | PBANKA_080720 | conserved Plasmodium protein, unknown function | Yes |
| 266 | PBANKA_110390 | conserved Plasmodium protein, unknown function | Yes |
| 267 | PBANKA_082530 | conserved Plasmodium protein, unknown function | No |
| 268 | PBANKA_082590 | conserved Plasmodium protein, unknown function | Yes |
| 269 | PBANKA_123060 | conserved Plasmodium protein, unknown function | Yes |
| 270 | PBANKA_123070 | conserved Plasmodium protein, unknown function | Yes |
| 271 | PBANKA_051040 | conserved Plasmodium protein, unknown function | Yes |
| 272 | PBANKA_051050 | conserved Plasmodium protein, unknown function | Yes |
| 273 | PBANKA_123120 | conserved Plasmodium protein, unknown function | Yes |
| 274 | PBANKA_124350 | conserved Plasmodium protein, unknown function | Yes |
| 275 | PBANKA_050950 | conserved Plasmodium protein, unknown function | Yes |
| 276 | PBANKA_010700 | conserved Plasmodium protein, unknown function | Yes |
| 277 | PBANKA_020240 | conserved Plasmodium protein, unknown function | Yes |
| 278 | PBANKA_020220 | conserved Plasmodium protein, unknown function | Yes |
| 279 | PBANKA_020170 | conserved Plasmodium protein, unknown function | Yes |
| 280 | PBANKA_122320 | conserved Plasmodium protein, unknown function | Yes |
| 281 | PBANKA_071320 | conserved Plasmodium protein, unknown function | Yes |
| 282 | PBANKA_071250 | conserved Plasmodium protein, unknown function | Yes |
| 283 | PBANKA_060300 | conserved Plasmodium protein, unknown function | Yes |
| 284 | PBANKA_060310 | conserved Plasmodium protein, unknown function | Yes |
| 285 | PBANKA_091780 | conserved Plasmodium protein, unknown function | No |
| 286 | PBANKA_031150 | conserved Plasmodium protein, unknown function | Yes |
| 287 | PBANKA_031200 | conserved Plasmodium protein, unknown function | Yes |
| 288 | PBANKA_094110 | conserved Plasmodium protein, unknown function | Yes |
| 289 | PBANKA_093880 | conserved Plasmodium protein, unknown function | Yes |
| 290 | PBANKA_093750 | conserved Plasmodium protein, unknown function | Yes |
| 291 | PBANKA_082920 | conserved Plasmodium protein, unknown function | Yes |
| 292 | PBANKA_102570 | conserved Plasmodium protein, unknown function | Yes |
| 293 | PBANKA_082930 | conserved Plasmodium protein, unknown function | No |
| 294 | PBANKA_110460 | conserved Plasmodium protein, unknown function | No |
| 295 | PBANKA_090860 | conserved Plasmodium protein, unknown function | Yes |
| 296 | PBANKA_090690 | conserved Plasmodium protein, unknown function | No |
| 297 | PBANKA_090700 | conserved Plasmodium protein, unknown function | Yes |
| 298 | PBANKA_082740 | conserved Plasmodium protein, unknown function | No |
| 299 | PBANKA_124140 | conserved Plasmodium protein, unknown function | Yes |
| 300 | PBANKA_124130 | conserved Plasmodium protein, unknown function | No |
| 301 | PBANKA_041420 | conserved Plasmodium protein, unknown function | Yes |
| 302 | PBANKA_041430 | conserved Plasmodium protein, unknown function | Yes |
| 303 | PBANKA_142920 | conserved Plasmodium protein, unknown function | No |
| 304 | PBANKA_122990 | conserved Plasmodium protein, unknown function | No |
| 305 | PBANKA_134250 | conserved Plasmodium protein, unknown function | Yes |
| 306 | PBANKA_144360 | conserved Plasmodium protein, unknown function | Yes |
| 307 | PBANKA_144370 | conserved Plasmodium protein, unknown function | Yes |
| 308 | PBANKA_113150 | conserved Plasmodium protein, unknown function | Yes |
| 309 | PBANKA_123370 | conserved Plasmodium protein, unknown function | Yes |
| 310 | PBANKA_145910 | conserved Plasmodium protein, unknown function | Yes |
| 311 | PBANKA_145770 | conserved Plasmodium protein, unknown function | Yes |
| 312 | PBANKA_114140 | conserved Plasmodium protein, unknown function | Yes |
| 313 | PBANKA_060150 | conserved Plasmodium protein, unknown function | Yes |
| 314 | PBANKA_040690 | conserved Plasmodium protein, unknown function | Yes |
| 315 | PBANKA_146390 | conserved Plasmodium protein, unknown function | Yes |
| 316 | PBANKA_134690 | conserved Plasmodium protein, unknown function | No |
| 317 | PBANKA_111980 | conserved Plasmodium protein, unknown function | Yes |
| 318 | PBANKA_083420 | conserved Plasmodium protein, unknown function | Yes |
| 319 | PBANKA_120070 | conserved Plasmodium protein, unknown function | Yes |
| 320 | PBANKA_050300 | conserved Plasmodium protein, unknown function | Yes |
| 321 | PBANKA_050280 | conserved Plasmodium protein, unknown function | No |
| 322 | PBANKA_050550 | conserved Plasmodium protein, unknown function | Yes |
| 323 | PBANKA_050600 | conserved Plasmodium protein, unknown function | Yes |
| 324 | PBANKA_061870 | conserved Plasmodium protein, unknown function | Yes |
| 325 | PBANKA_134640 | conserved Plasmodium protein, unknown function | No |
| 326 | PBANKA_061780 | conserved Plasmodium protein, unknown function | Yes |
| 327 | PBANKA_070480 | conserved Plasmodium protein, unknown function | Yes |
| 328 | PBANKA_070490 | conserved Plasmodium protein, unknown function | Yes |
| 329 | PBANKA_070660 | conserved Plasmodium protein, unknown function | Yes |
| 330 | PBANKA_030310 | conserved Plasmodium protein, unknown function | Yes |
| 331 | PBANKA_112010 | conserved Plasmodium protein, unknown function | Yes |
| 332 | PBANKA_061850 | conserved Plasmodium protein, unknown function | Yes |
| 333 | PBANKA_101260 | conserved Plasmodium protein, unknown function | Yes |
| 334 | PBANKA_100990 | conserved Plasmodium protein, unknown function | Yes |
| 335 | PBANKA_111680 | conserved Plasmodium protein, unknown function | Yes |
| 336 | PBANKA_144120 | conserved Plasmodium protein, unknown function | Yes |
| 337 | PBANKA_051300 | conserved Plasmodium protein, unknown function | No |
| 338 | PBANKA_145670 | conserved Plasmodium protein, unknown function | Yes |
| 339 | PBANKA_133950 | conserved Plasmodium protein, unknown function | Yes |
| 340 | PBANKA_145980 | conserved Plasmodium protein, unknown function | Yes |
| 341 | PBANKA_134820 | conserved Plasmodium protein, unknown function | Yes |
| 342 | PBANKA_120280 | conserved Plasmodium protein, unknown function | No |
| 343 | PBANKA_120290 | conserved Plasmodium protein, unknown function | No |
| 344 | PBANKA_010450 | conserved Plasmodium protein, unknown function | Yes |
| 345 | PBANKA_030550 | conserved Plasmodium protein, unknown function | Yes |
| 346 | PBANKA_050380 | conserved Plasmodium protein, unknown function | Yes |
| 347 | PBANKA_114190 | conserved Plasmodium protein, unknown function | Yes |
| 348 | PBANKA_114380 | conserved Plasmodium protein, unknown function | Yes |
| 349 | PBANKA_050960 | conserved Plasmodium protein, unknown function | Yes |
| 350 | PBANKA_144420 | conserved Plasmodium protein, unknown function | No |
| 351 | PBANKA_144430 | conserved Plasmodium protein, unknown function | No |
| 352 | PBANKA_050180 | conserved Plasmodium protein, unknown function | No |
| 353 | PBANKA_031370 | conserved Plasmodium protein, unknown function | Yes |
| 354 | PBANKA_031350 | conserved Plasmodium protein, unknown function | Yes |
| 355 | PBANKA_102050 | conserved Plasmodium protein, unknown function | Yes |
| 356 | PBANKA_102080 | conserved Plasmodium protein, unknown function | Yes |
| 357 | PBANKA_070960 | conserved Plasmodium protein, unknown function | Yes |
| 358 | PBANKA_101390 | conserved Plasmodium protein, unknown function | Yes |
| 359 | PBANKA_135490 | conserved Plasmodium protein, unknown function | Yes |
| 360 | PBANKA_092120 | conserved Plasmodium protein, unknown function | Yes |
| 361 | PBANKA_082500 | conserved Plasmodium protein, unknown function | Yes |
| 362 | PBANKA_082510 | conserved Plasmodium protein, unknown function | Yes |
| 363 | PBANKA_071900 | conserved Plasmodium protein, unknown function, fragment | Yes |
| 364 | PBANKA_090230 | conserved Plasmodium protein, unknown function, fragment | Yes |
| 365 | PBANKA_091700 | conserved Plasmodium protein, unknown function, fragment | Yes |
| 366 | PBANKA_031550 | conserved Plasmodium protein, unknown function, pseudogene | No |
| 367 | PBANKA_041540 | conserved Plasmodium protein, unknown function, pseudogene | Yes |
| 368 | PBANKA_031060 | conserved protein, unknown function | Yes |
| 369 | PBANKA_020090 | conserved rodent malaria protein, unknown function | Yes |
| 370 | PBANKA_090660 | coproporphyrinogen oxidase, putative | No |
| 371 | PBANKA_101700 | CorA-like Mg2 transporter protein, putative | Yes |
| 372 | PBANKA_082110 | CS domain protein, putative | Yes |
| 373 | PBANKA_123320 | cyclin, putative | Yes |
| 374 | PBANKA_081910 | cytochrome b5, putative | Yes |
| 375 | PBANKA_123410 | cytochrome c oxidase assembly protein, putative | No |
| 376 | PBANKA_060260 | cytochrome c1 heme lyase, putative | No |
| 377 | PBANKA_050450 | cytoplasmic dynein intermediate chain, putative | No |
| 378 | PBANKA_061880 | DEAD box helicase, putative | Yes |
| 379 | PBANKA_031510 | DEAD/DEAH helicase, putative | No |
| 380 | PBANKA_123630 | DEAD-box subfamily ATP-dependent helicase, putative | Yes |
| 381 | PBANKA_083460 | delta tubulin, putative | No |
| 382 | PBANKA_111330 | deoxyribodipyrimidine photolyase, putative | No |
| 383 | PBANKA_050580 | deoxyribose-phosphate aldolase, putative | Yes |
| 384 | PBANKA_134000 | dihydrofolate synthase/folylpolyglutamate synthase, putative | Yes |
| 385 | PBANKA_050500 | dihydrolipoamide acyltransferase, putative | Yes |
| 386 | PBANKA_081970 | dihydrouridine synthase, putative | Yes |
| 387 | PBANKA_093130 | dipeptidyl aminopeptidase, putative | Yes |
| 388 | PBANKA_101520 | diphthamide synthesis protein, putative | No |
| 389 | PBANKA_083490 | DNA excision-repair helicase, putative | Yes |
| 390 | PBANKA_021040 | DNA mismatch repair protein, putative | Yes |
| 391 | PBANKA_122680 | DNA repair protein rad54, putative | Yes |
| 392 | PBANKA_071830 | DNA replication licensing factor, putative | Yes |
| 393 | PBANKA_133040 | DNA-3-methyladenine glycosylase, putative | No |
| 394 | PBANKA_092300 | DNA-dependent RNA polymerase, putative | No |
| 395 | PBANKA_101910 | DNA-directed DNA polymerase, putative | Yes |
| 396 | PBANKA_030320 | DNA-directed RNA polymerase II 16 kDa subunit, putative | Yes |
| 397 | PBANKA_102110 | DnaJ protein, putative | Yes |
| 398 | PBANKA_030840 | dolichol-linked oligosaccharide biosynthesis enzyme, putative | Yes |
| 399 | PBANKA_030220 | dynein light chain, putative | No |
| 400 | PBANKA_040560 | EB1 homolog, putative | Yes |
| 401 | PBANKA_081420 | elongation factor 1-beta, putative | Yes |
| 402 | PBANKA_010120 | elongation factor G, putative (EF-G) | Yes |
| 403 | PBANKA_134560 | elongation factor Tu, putative | Yes |
| 404 | PBANKA_122980 | enoyl-acyl carrier reductase (FABI) | No |
| 405 | PBANKA_101410 | exodeoxyribonuclease III, putative | Yes |
| 406 | PBANKA_070650 | exonuclease, putative | Yes |
| 407 | PBANKA_141440 | exportin-T, putative | Yes |
| 408 | PBANKA_142230 | FAD-dependent monooxygenase, putative | Yes |
| 409 | PBANKA_020430 | fatty acid elongation protein, GNS1/SUR4 family, putative, pseudogene | Yes |
| 410 | PBANKA_061790 | ferrodoxin reductase-like protein, putative | Yes |
| 411 | PBANKA_146040 | FK506-binding protein (FKBP)-type peptidyl-prolyl isomerase, putative (FKBP35) | Yes |
| 412 | PBANKA_082410 | flavodoxin-like protein | Yes |
| 413 | PBANKA_082810 | fumarate hydratase, putative | No |
| 414 | PBANKA_131270 | gamete egress and sporozoite traversal protein (GEST) | Yes |
| 415 | PBANKA_010130 | geranylgeranyltransferase, putative | Yes |
| 416 | PBANKA_081580 | GINS complex subunit Psf3, putative | No |
| 417 | PBANKA_133890 | glideosome associated protein with multiple membrane spans 1, putative (GAPM1) | Yes |
| 418 | PBANKA_143760 | glideosome-associated protein 45, putative | Yes |
| 419 | PBANKA_136200 | glutamate - tRNA ligase, putative | Yes |
| 420 | PBANKA_102340 | glutathione reductase, putative (GR) | Yes |
| 421 | PBANKA_111180 | glutathione synthetase (GS) | Yes |
| 422 | PBANKA_041040 | glycogen synthase kinase 3 (GSK3) | Yes |
| 423 | PBANKA_122430 | glycosyltransferase, putative | Yes |
| 424 | PBANKA_134570 | GPI transamidase subunit PIG-U, putative | Yes |
| 425 | PBANKA_070190 | GPI-anchored micronemal antigen, putative (GAMA) | Yes |
| 426 | PBANKA_122570 | GTPase activating protein, putative | No |
| 427 | PBANKA_093030 | GTP-binding nuclear protein, putative | Yes |
| 428 | PBANKA_031000 | heat shock 40 kDa protein, putative | Yes |
| 429 | PBANKA_060960 | heat shock protein 20, putative | Yes |
| 430 | PBANKA_081890 | heat shock protein 70, putative | Yes |
| 431 | PBANKA_092990 | heat shock protein 90, putative | No |
| 432 | PBANKA_121910 | heat shock protein 90, putative | Yes |
| 433 | PBANKA_071190 | heat shock protein, putative (HSP70) | Yes |
| 434 | PBANKA_111380 | helicase, putative (UIS26) | Yes |
| 435 | PBANKA_030250 | hexose transporter (HT) | Yes |
| 436 | PBANKA_083480 | histidyl-tRNA synthetase, putative | Yes |
| 437 | PBANKA_082650 | histone deacetylase, putative (HDAC1) | Yes |
| 438 | PBANKA_111700 | histone H2A, putative (H2A) | No |
| 439 | PBANKA_142060 | histone H2B, putative | No |
| 440 | PBANKA_094180 | histone H2B, putative (H2B) | Yes |
| 441 | PBANKA_134840 | histone H3 variant, putative (CenH3) | Yes |
| 442 | PBANKA_020340 | human hepatopoietin-like protein, putative | Yes |
| 443 | PBANKA_090710 | inner membrane complex protein 1b (IMC1b) | Yes |
| 444 | PBANKA_041410 | inorganic pyrophosphatase, putative | Yes |
| 445 | PBANKA_113080 | inositol-polyphosphate 5-phosphatase, putative | Yes |
| 446 | PBANKA_092980 | insulinase, putative | No |
| 447 | PBANKA_092980 | insulinase, putative | No |
| 448 | PBANKA_134770 | isoleucine - tRNA ligase, putative | Yes |
| 449 | PBANKA_060950 | kinesin-related protein, putative | Yes |
| 450 | PBANKA_080770 | kinesin-related protein, putative | Yes |
| 451 | PBANKA_103520 | LCCL domain-containing protein (CCp3) | Yes |
| 452 | PBANKA_071400 | meiotic recombination protein DMC1-like protein, putative | Yes |
| 453 | PBANKA_124060 | membrane skeletal protein, putative | Yes |
| 454 | PBANKA_070710 | membrane skeletal protein, putative | Yes |
| 455 | PBANKA_120200 | membrane skeletal protein, putative | Yes |
| 456 | PBANKA_111960 | merozoite surface protein 10, putative (MSP10) | Yes |
| 457 | PBANKA_051280 | merozoite TRAP-like protein, putative (MTRAP) | No |
| 458 | PBANKA_081530 | met-10 like protein, putative | Yes |
| 459 | PBANKA_123130 | metabolite/drug transporter, putative | Yes |
| 460 | PBANKA_113140 | metacaspase 1 (MCA1) | Yes |
| 461 | PBANKA_122150 | mitochondrial ribosomal protein L1 precursor, putative | Yes |
| 462 | PBANKA_041330 | mitochondrial ribosomal protein L29/L47 precursor, putative | Yes |
| 463 | PBANKA_061340 | mitochondrial ribosomal protein S12 precursor, putative | No |
| 464 | PBANKA_051130 | mitochondrial ribosomal protein S22 precursor, putative | No |
| 465 | PBANKA_091160 | mitochondrial ribosomal protein S9 precursor, putative | No |
| 466 | PBANKA_101330 | mitogen-activated protein kinase 1 (MAP1) | No |
| 467 | PBANKA_083380 | mRNA processing protein, putative | Yes |
| 468 | PBANKA_123350 | mRNA-binding protein PUF1 (PUF1) | Yes |
| 469 | PBANKA_133960 | MSF1-like protein, putative | Yes |
| 470 | PBANKA_145950 | myosin light chain 1, putative,myosin A tail domain interacting protein MTIP, putative (MTIP) | Yes |
| 471 | PBANKA_110330 | myosin pfm-b, putative | No |
| 472 | PBANKA_114340 | NADH-cytochrome b5 reductase, putative | Yes |
| 473 | PBANKA_020410 | N-terminal acetyltransferase, putative | No |
| 474 | PBANKA_134720 | nucleotidyltransferase, putative | Yes |
| 475 | PBANKA_121810 | oocyst capsule protein (Cap380) | Yes |
| 476 | PBANKA_031250 | origin recognition complex subunit 5, putative (ORC5) | Yes |
| 477 | PBANKA_010740 | ornithine aminotransferase, putative (OAT) | Yes |
| 478 | PBANKA_050740 | orotidine-monophosphate-decarboxylase, putative (OMPDC) | Yes |
| 479 | PBANKA_146300 | osmiophilic body protein (G377) | Yes |
| 480 | PBANKA_082400 | OTU-like cysteine protease, putative | Yes |
| 481 | PBANKA_080940 | P1 nuclease, putative | Yes |
| 482 | PBANKA_102260 | pantothenate kinase, putative | Yes |
| 483 | PBANKA_061140 | pantothenate kinase, putative | Yes |
| 484 | PBANKA_020920 | parasite-infected erythrocyte surface protein (PIESP15) | No |
| 485 | PBANKA_061960 | PelOta protein homologue, putative | Yes |
| 486 | PBANKA_100650 | peptidase, M22 family, putative | Yes |
| 487 | PBANKA_092850 | peptidase, putative | Yes |
| 488 | PBANKA_080930 | peptide deformylase, putative | Yes |
| 489 | PBANKA_082420 | perforin like protein 3 (PPLP3) | Yes |
| 490 | PBANKA_071140 | perforin like protein 4 (PPLP4) | Yes |
| 491 | PBANKA_071160 | perforin like protein 5 (PPLP5) | Yes |
| 492 | PBANKA_010770 | permease, putative | Yes |
| 493 | PBANKA_020460 | photosensitized INA-labeled protein 1, putative | Yes |
| 494 | PBANKA_122250 | plasmepsin X | No |
| 495 | PBANKA_061030 | polyubiquitin, putative | Yes |
| 496 | PBANKA_060800 | porphobilinogen deaminase, putative | No |
| 497 | PBANKA_110830 | Pre-mRNA-processing ATP-dependent RNA helicase prp5, putative (PRP5) | Yes |
| 498 | PBANKA_122650 | proteasome beta-subunit, putative | Yes |
| 499 | PBANKA_080820 | proteasome component C8, putative | Yes |
| 500 | PBANKA_134590 | protein kinase, putative | Yes |
| 501 | PBANKA_082680 | protein kinase, putative | No |
| 502 | PBANKA_110320 | protein kinase, putative | Yes |
| 503 | PBANKA_040940 | protein kinase, putative (PKRP) | No |
| 504 | PBANKA_031100 | protein transport protein Sec31, putative (SEC31) | Yes |
| 505 | PBANKA_123080 | pyridoxal kinase-like protein, putative | Yes |
| 506 | PBANKA_134200 | Qa-SNARE protein, putative | Yes |
| 507 | PBANKA_111350 | Rab1a, putative | Yes |
| 508 | PBANKA_092470 | RAP protein, putative | No |
| 509 | PBANKA_051380 | RAP protein, putative | Yes |
| 510 | PBANKA_144130 | RAP protein, putative | Yes |
| 511 | PBANKA_020250 | replication factor c protein, putative | Yes |
| 512 | PBANKA_123610 | ribosomal large subunit pseudouridylate synthase, putative | Yes |
| 513 | PBANKA_030570 | ribosome-recycling factor, putative (RRF) | Yes |
| 514 | PBANKA_050570 | RNA helicase, putative | Yes |
| 515 | PBANKA_103630 | RNA-binding protein, putative | Yes |
| 516 | PBANKA_050460 | S-adenosyl-L-methionine-dependent methyltransferase, putative | Yes |
| 517 | PBANKA_030690 | Sec61-gamma subunit of protein translocation complex, putative | No |
| 518 | PBANKA_103780 | secreted ookinete adhesive protein (SOAP) | Yes |
| 519 | PBANKA_061920 | secreted ookinete protein, putative (PSOP1) | Yes |
| 520 | PBANKA_111340 | secreted ookinete protein, putative (PSOP12) | Yes |
| 521 | PBANKA_114370 | secreted ookinete protein, putative (PSOP2) | Yes |
| 522 | PBANKA_112900 | secreted ookinete protein, putative (PSOP6) | Yes |
| 523 | PBANKA_135340 | secreted ookinete protein, putative (PSOP7) | Yes |
| 524 | PBANKA_020350 | selenocysteine-specific elongation factor selB homologue, putative | Yes |
| 525 | PBANKA_146050 | serine/threonine protein kinase, putative | Yes |
| 526 | PBANKA_082960 | serine/threonine protein kinase, putative | Yes |
| 527 | PBANKA_040740 | serine/threonine protein kinase, putative (ARK2) | No |
| 528 | PBANKA_052320 | serpentine receptor, putative (SR12) | No |
| 529 | PBANKA_110770 | SET domain protein, putative | Yes |
| 530 | PBANKA_134620 | signal peptidase 21 kDa subunit, putative (SP21) | Yes |
| 531 | PBANKA_071430 | small heat shock protein HSP20 (HSP20) | Yes |
| 532 | PBANKA_092280 | small nuclear ribonucleoprotein D1, putative (SNRPD1) | Yes |
| 533 | PBANKA_031520 | small nuclear ribonucleoprotein Sm D2, putative (SNRPD2) | No |
| 534 | PBANKA_070930 | small ribosomal subunit processing microtubule-associated protein, putative | Yes |
| 535 | PBANKA_131230 | SNARE protein, putative | Yes |
| 536 | PBANKA_071080 | Snf2-related CBP activator, putative (SRCAP) | No |
| 537 | PBANKA_100620 | sporozoite invasion-associated protein 1 (SIAP1) | No |
| 538 | PBANKA_081070 | subpellicular microtubule protein 1, putative (SPM1) | Yes |
| 539 | PBANKA_144500 | subpellicular microtubule protein 2, putative (SPM2) | Yes |
| 540 | PBANKA_110710 | subtilisin-like protease 1, putative (SUB1) | Yes |
| 541 | PBANKA_011200 | syntaxin binding protein, putative | Yes |
| 542 | PBANKA_094280 | syntaxin, putative | Yes |
| 543 | PBANKA_020180 | TatD-like deoxyribonuclease, putative | Yes |
| 544 | PBANKA_040520 | T-complex protein beta subunit, putative | No |
| 545 | PBANKA_082570 | telomeric repeat binding factor 1, putative | Yes |
| 546 | PBANKA_082520 | thiamin pyrophosphokinase, putative | Yes |
| 547 | PBANKA_092220 | ThiF family protein, putative | Yes |
| 548 | PBANKA_082020 | thioredoxin, putative | Yes |
| 549 | PBANKA_110800 | transcription factor IIb, putative | No |
| 550 | PBANKA_113010 | Transcription factor Tfb4, putative | No |
| 551 | PBANKA_083520 | transcription factor with AP2 domain(s), putative | Yes |
| 552 | PBANKA_052170 | transcription factor with AP2 domain, putative (ApiAP2) | Yes |
| 553 | PBANKA_051400 | transcription factor, putative | Yes |
| 554 | PBANKA_134380 | transcriptional regulatory protein sir2a (Sir2A) | Yes |
| 555 | PBANKA_020840 | transporter, putative | No |
| 556 | PBANKA_050400 | tRNA methyltransferase, putative | Yes |
| 557 | PBANKA_111370 | tubulin-tyrosine ligase, putative | Yes |
| 558 | PBANKA_092880 | U2 snRNP auxiliary factor, small subunit, putative | Yes |
| 559 | PBANKA_093780 | U3/U14 snoRNA-associated small subunit rRNA processing protein, putative | Yes |
| 560 | PBANKA_121480 | U4/U6.U5 tri-snRNP-associated protein 1, putative (SART1) | Yes |
| 561 | PBANKA_134020 | U6 snRNA-associated Sm-like protein LSm6, putative (LSM6) | Yes |
| 562 | PBANKA_090450 | ubiquitin activating enzyme (E1) subunit Aos1, putative | Yes |
| 563 | PBANKA_071380 | ubiquitin-activating enzyme, putative | No |
| 564 | PBANKA_060280 | ubiquitin-conjugating enzyme e2, putative | Yes |
| 565 | PBANKA_110490 | UDP-N-acetyl glucosamine:UMP antiporter, putative | Yes |
| 566 | PBANKA_123400 | vacuolar ATP synthetase, putative | Yes |
| 567 | PBANKA_031310 | vacuolar sorting protein VPS45, putative | Yes |
| 568 | PBANKA_122890 | von willebrand factor a-domain-related protein (WARP) | Yes |
| 569 | PBANKA_020970 | zinc carboxy peptidase, putative | Yes |
| 570 | PBANKA_123270 | zinc finger protein, putative | Yes |
| 571 | PBANKA_083330 | zinc finger protein, putative | Yes |
| 572 | PBANKA_071230 | zinc finger protein, putative | No |
| 573 | PBANKA_113490 | zinc finger protein, putative | Yes |

^a^ In experiment 1, ChIP-seq was performed in illumina. Obtained reads were mapped on the reference sequence with a condition of Peak-calling performed using MACS2 using IP and INPUT data under the condition of FDR < 0.01　and fold enrichment over input > 5, and 1,540 peaks were identified within the *P. berghei* genome. Target genes of AP2-O were identified from the summits of the peaks using the same in-house programs as those in experiment 2. Gene ID and functional annotation were attributed to each gene according to those in PlasmoDB ver.12.0. Data were sorted in alphabetical order of attributed functional annotations. Target genes that are identical to those identified in experiment 2 are indicated by “Yes” in the last column.

**Table S3. Six-base sequences enriched around the summits of AP2-O peaks identified using ChIP-seq analysis (experiment 2)**^a^

|  | sequences | p-values | Corresponding motifs |
| --- | --- | --- | --- |
| 1 | GCTAGC | 4.94E-324 | [TC] [AG]GC[TC] [AG] |
| 2 | TAGCTA | 2.48E-260 | [TC] [AG]GC[TC] [AG] |
| 3 | AGCTAG | 5.46E-252 | [TC] [AG]GC[TC] [AG] |
| 4 | CTAGCT | 3.19E-244 | [TC] [AG]GC[TC] [AG] |
| 5 | GGCTAG | 1.16E-128 | [TC] [AG]GC[TC] [AG] |
| 6 | CTAGCC | 6.15E-116 | [TC] [AG]GC[TC] [AG] |
| 7 | TGGCTA | 8.04E-116 | [TC] [AG]GC[TC] [AG] |
| 8 | TAGCCA | 7.05E-107 | [TC] [AG]GC[TC] [AG] |
| 9 | ATAGCT | 4.94E-94 | [TC] [AG]GC[TC] [AG] |
| 10 | AGCTAT | 1.88E-86 | [TC] [AG]GC[TC] [AG] |
| 11 | AGCCAA | 3.57E-62 | [TC] [AG]GC[TC] [AG] |
| 12 | TTGGCT | 3.94E-62 | [TC] [AG]GC[TC] [AG] |
| 13 | AGCTAA | 6.24E-57 | [TC] [AG]GC[TC] [AG] |
| 14 | GCTAGT | 8.40E-57 | [TC] [AG]GC[TC] [AG] |
| 15 | ATGAAC | 2.68E-55 | TGAACA |
| 16 | TTAGCT | 4.93E-54 | [TC] [AG]GC[TC] [AG] |
| 17 | TGTTCA | 1.51E-51 | T GAACA |
| 18 | AATAGC | 3.37E-51 | [TC] [AG]GC[TC] [AG] |
| 19 | TGAACA | 3.41E-51 | TGAACA |
| 20 | GCTATT | 3.72E-48 | [TC] [AG]GC[TC] [AG] |

^a^ Fisher’s exact tests were performed to compare the presence of each six-base sequence (second column) in 100-bp regions around the summits of identified peaks with its presence in other 100-bp genome regions. The results were ordered according to the p-values obtained from this analysis. Twenty sequences with the lowest p-values are shown. Corresponding motifs are indicated when at least four of the six bases matched with the primary or secondary motif (fourth column). Reverse complementary sequences (for example, tgaaca and tgttca) were considered to correspond to the same motif (i.e., tgaaca).

**Table S4. Six-base sequences enriched around the summits of AP2-O peaks identified by ChIP-seq analysis in experiment 1**^a^

|  | sequences | p-values | Corresponding motif^[[1]](#footnote-1)^ |
| --- | --- | --- | --- |
| 1 | TAGCTA | 0 | [TC] [AG]GC[TC] [AG] |
| 2 | GCTAGC | 0 | [TC] [AG]GC[TC] [AG] |
| 3 | AGCTAG | 0 | [TC] [AG]GC[TC] [AG] |
| 4 | CTAGCT | 0 | [TC] [AG]GC[TC] [AG] |
| 5 | GGCTAG | 1.62E-179 | [TC] [AG]GC[TC] [AG] |
| 6 | TGGCTA | 3.56E-164 | [TC] [AG]GC[TC] [AG] |
| 7 | CTAGCC | 4.23E-155 | [TC] [AG]GC[TC] [AG] |
| 8 | TAGCCA | 1.61E-148 | [TC] [AG]GC[TC] [AG] |
| 9 | ATAGCT | 3.91E-120 | [TC] [AG]GC[TC] [AG] |
| 10 | AGCTAT | 5.57E-110 | [TC] [AG]GC[TC] [AG] |
| 11 | AGCCAA | 2.96E-89 | [TC] [AG]GC[TC] [AG] |
| 12 | GCTAGT | 7.69E-83 | [TC] [AG]GC[TC] [AG] |
| 13 | AGCTAA | 9.55E-82 | [TC] [AG]GC[TC] [AG] |
| 14 | TTGGCT | 1.69E-79 | [TC] [AG]GC[TC] [AG] |
| 15 | TTAGCT | 1.46E-72 | [TC] [AG]GC[TC] [AG] |
| 16 | ACTAGC | 1.30E-70 | [TC] [AG]GC[TC] [AG] |
| 17 | ATGAAC | 8.82E-69 | TGAACA |
| 18 | TGAACA | 5.58E-66 | TGAACA |
| 19 | CAGCTA | 1.94E-64 | [TC] [AG]GC[TC] [AG] |
| 20 | TGTTCA | 2.81E-63 | TGAACA |

^a^ Fisher’s exact tests were performed to determine the probability of the occurrence of each six-base sequence (second column) as in Table S3. The results were ordered according to the p-values obtained, and AP2-O binding motif sequences that frequently appear in the region were identified (fourth column). Reverse complementary sequences (for example, tgaaca and tgttca) were considered to correspond to the same motif (i.e., tgaaca).

**Table S5. Six-base sequences enriched around the summits of AP2-O peaks without the primary motif (experiment 2)**^a^

|  | sequences | p-values | Corresponding motifs |
| --- | --- | --- | --- |
| 1 | tgaaca | 1.92E-21 | TG[ATC]ACA |
| 2 | tgcaca | 2.56E-18 | TG[ATC]ACA |
| 3 | atgaac | 2.58E-18 | TG[ATC]ACA |
| 4 | tgttca | 3.44E-18 | TG[ATC]ACA |
| 5 | gttcat | 4.83E-18 | TG[ATC]ACA |
| 6 | ctgaac | 4.45E-17 | TG[ATC]ACA |
| 7 | tgtaca | 9.07E-17 | TG[ATC]ACA |
| 8 | gttcag | 3.65E-15 | TG[ATC]ACA |
| 9 | ctgttc | 3.66E-15 | TG[ATC]ACA |
| 10 | atgtac | 6.84E-14 | TG[ATC]ACA |
| 11 | gcacac | 1.72E-11 | TG[ATC]ACA |
| 12 | gtgcat | 2.66E-11 | TG[ATC]ACA |
| 13 | gtacat | 3.42E-11 | TG[ATC]ACA |
| 14 | agtcag | 1.34E-10 | – |
| 15 | catgta | 1.43E-10 | – |
| 16 | tagcga | 3.80E-10 | – |
| 17 | caagtc | 4.77E-10 | – |
| 18 | tgcatg | 6.02E-09 | – |
| 19 | tcagaa | 1.62E-08 | – |
| 20 | atgcac | 2.91E-08 | TG[ATC]ACA |

^a^ The same analysis as in Table S3 was performed on AP2-O peaks without the primary motif [TC] [AG]GC[TC] [AG]. Twenty sequences with the lowest p-values are shown. Reverse complementary sequences (for example, tgaaca and tgttca) were considered to correspond to the same motif (i.e., tgaaca).

**Table S6. Six-base sequences enriched around the summits of AP2-O peaks without the primary motif [TC] [AG]GC[TC] [AG] in experiment 1**^a^

|  | sequences | p-values | Corresponding motifs |
| --- | --- | --- | --- |
| 1 | tgaaca | 1.06E-38 | TG[ATC]ACA |
| 2 | atgaac | 4.15E-34 | TG[ATC]ACA |
| 3 | tgcaca | 1.43E-30 | TG[ATC]ACA |
| 4 | ctgaac | 6.94E-30 | TG[ATC]ACA |
| 5 | tgttca | 5.50E-29 | TG[ATC]ACA |
| 6 | atgtac | 1.05E-28 | TG[ATC]ACA |
| 7 | gttcat | 8.46E-28 | TG[ATC]ACA |
| 8 | acatgt | 4.14E-26 | – |
| 9 | tgtaca | 2.91E-25 | TG[ATC]ACA |
| 10 | gtgcat | 1.20E-24 | TG[ATC]ACA |
| 11 | tgtgca | 4.62E-24 | TG[ATC]ACA |
| 12 | gttcag | 1.35E-23 | TG[ATC]ACA |
| 13 | gtacat | 4.60E-23 | TG[ATC]ACA |
| 14 | ctgttc | 8.14E-23 | TG[ATC]ACA |
| 15 | catgta | 1.07E-22 | – |
| 16 | tacatg | 4.64E-22 | – |
| 17 | tgcatg | 3.46E-20 | – |
| 18 | catgca | 7.61E-20 | – |
| 19 | cctgaa | 1.28E-16 | – |
| 20 | cctgac | 1.42E-16 | – |

^a^ The same analysis as in Table S3 was performed on AP2-O peaks without the primary motif [TC] [AG]GC[TC] [AG] in experiment 1. Twenty sequences with the lowest p-values are shown.

**Table S7. Classification of target genes**^a^

|  | categories |  |  | Gene ID | KO | Functional Annotations |
| --- | --- | --- | --- | --- | --- | --- |
| 1 | pellicle | 1 | IMC1-related | PBANKA_090710 |  | inner membrane complex protein 1b (IMC1b) |
| 2 |  | 2 |  | PBANKA_070710 | ko | membrane skeletal protein, putative |
| 3 |  | 3 |  | PBANKA_120200 |  | membrane skeletal protein, putative |
| 4 |  | 4 |  | PBANKA_124060 |  | membrane skeletal protein, putative |
| 5 |  | 5 |  | PBANKA_121910 |  | heat shock protein 90, putative |
| 6 |  | 6 |  | PBANKA_136440 |  | conserved Plasmodium protein, unknown function |
| 7 |  | 7 |  | PBANKA_102570 |  | conserved Plasmodium protein, unknown function |
| 8 |  | 8 |  | PBANKA_135490 |  | conserved Plasmodium protein, unknown function |
| 9 |  | 9 | other IMC proteins | PBANKA_120940 |  | conserved Plasmodium protein, unknown function (ISP1/2) |
| 10 |  | 10 |  | PBANKA_132430 |  | conserved Plasmodium protein, unknown function (ISP3) |
| 11 |  | 11 |  | PBANKA_020460 |  | photosensitized INA-labeled protein 1, putative |
| 12 |  | 12 |  | PBANKA_133890 |  | glideosome associated protein with multiple membrane spans 1, putative (GAPM1) |
| 13 |  | 13 |  | PBANKA_111530 |  | glideosome-associated protein 40, putative (GAP40) |
| 14 |  | 14 |  | PBANKA_143760 |  | glideosome-associated protein 45, putative |
| 15 |  | 15 |  | PBANKA_081900 | * | secreted acid phosphatase, putative,glideosome-associated protein 50, putative (GAP50) |
| 16 |  | 16 |  | PBANKA_145950 |  | myosin light chain 1, putative,myosin A tail domain interacting protein MTIP, putative (MTIP) |
| 17 |  | 17 |  | PBANKA_145120 |  | p25-alpha family protein, putative (apicortin) |
| 18 |  | 18 |  | PBANKA_110310 |  | actin-depolymerizing factor 1 (ADF1) |
| 19 |  | 19 |  | PBANKA_071430 |  | small heat shock protein HSP20 (HSP20) |
| 20 |  | 20 | subpellicle | PBANKA_111370 |  | tubulin-tyrosine ligase, putative |
| 21 |  | 21 |  | PBANKA_081070 |  | subpellicular microtubule protein 1, putative (SPM1) |
| 22 |  | 22 |  | PBANKA_144500 |  | subpellicular microtubule protein 2, putative (SPM2) |
| 23 | aicomplexa | 1 | PUA1 | PBANKA_031350 |  | conserved Plasmodium protein, unknown function |
| 24 |  | 2 | PUA2 | PBANKA_040690 |  | conserved Plasmodium protein, unknown function |
| 25 |  | 3 | PUA3 | PBANKA_041540 |  | conserved Plasmodium protein, unknown function |
| 26 |  | 4 | PUA4 | PBANKA_050520 |  | conserved Plasmodium protein, unknown function |
| 27 |  | 5 | PUA5 | PBANKA_060340 |  | conserved Plasmodium protein, unknown function |
| 28 |  | 6 | PUA6 | PBANKA_061120 |  | conserved Plasmodium protein, unknown function |
| 29 |  | 7 | PUA7 | PBANKA_061210 |  | conserved Plasmodium protein, unknown function |
| 30 |  | 8 | PUA8 | PBANKA_061640 |  | conserved Plasmodium protein, unknown function |
| 31 |  | 9 | PUA9 | PBANKA_061650 |  | conserved Plasmodium protein, unknown function |
| 32 |  | 10 | PUA10 | PBANKA_070960 |  | conserved Plasmodium protein, unknown function |
| 33 |  | 11 | PUA11 | PBANKA_071110 |  | conserved Plasmodium protein, unknown function |
| 34 |  | 12 | PUA12 | PBANKA_071450 |  | conserved Plasmodium protein, unknown function |
| 35 |  | 13 | PUA13 | PBANKA_072100 |  | conserved Plasmodium protein, unknown function |
| 36 |  | 14 | PUA14 | PBANKA_081020 |  | conserved Plasmodium protein, unknown function |
| 37 |  | 15 | PUA15 | PBANKA_082510 |  | conserved Plasmodium protein, unknown function |
| 38 |  | 16 | PUA16 | PBANKA_082920 |  | conserved Plasmodium protein, unknown function |
| 39 |  | 17 | PUA17 | PBANKA_083040 |  | conserved Plasmodium protein, unknown function |
| 40 |  | 18 | PUA18 | PBANKA_091600 |  | conserved Plasmodium protein, unknown function |
| 41 |  | 19 | PUA19 | PBANKA_092120 |  | conserved Plasmodium protein, unknown function |
| 42 |  | 20 | PUA20 | PBANKA_092240 |  | conserved Plasmodium protein, unknown function |
| 43 |  | 21 | PUA21 | PBANKA_093110 |  | conserved Plasmodium protein, unknown function |
| 44 |  | 22 | PUA22 | PBANKA_101470 |  | conserved Plasmodium protein, unknown function |
| 45 |  | 23 | PUA23 | PBANKA_110590 |  | conserved Plasmodium protein, unknown function |
| 46 |  | 24 | PUA24 | PBANKA_112460 |  | conserved Plasmodium protein, unknown function |
| 47 |  | 25 | PUA25 | PBANKA_122140 |  | conserved Plasmodium protein, unknown function |
| 48 |  | 26 | PUA26 | PBANKA_122540 | ko | IAAP |
| 49 |  | 27 | PUA27 | PBANKA_123460 |  | conserved Plasmodium protein, unknown function |
| 50 |  | 28 | PUA28 | PBANKA_124010 |  | conserved Plasmodium protein, unknown function |
| 51 |  | 29 | PUA29 | PBANKA_124290 |  | conserved Plasmodium protein, unknown function |
| 52 |  | 30 | PUA30 | PBANKA_131130 |  | conserved Plasmodium protein, unknown function |
| 53 |  | 31 | PUA31 | PBANKA_131240 |  | conserved Plasmodium protein, unknown function |
| 54 |  | 32 | PUA32 | PBANKA_134700 |  | conserved Plasmodium protein, unknown function |
| 55 |  | 33 | PUA33 | PBANKA_135240 |  | conserved Plasmodium protein, unknown function |
| 56 |  | 34 | PUA34 | PBANKA_135460 |  | conserved Plasmodium protein, unknown function |
| 57 |  | 35 | PUA35 | PBANKA_142290 |  | conserved Plasmodium protein, unknown function |
| 58 |  | 36 | PUA36 | PBANKA_145150 |  | conserved Plasmodium protein, unknown function |
| 59 | secretory proteins | 1 |  | PBANKA_143230 |  | cell traversal protein for ookinetes and sporozoites (CelTOS) |
| 60 |  | 2 |  | PBANKA_080050 |  | chitinase (CHT1) |
| 61 |  | 3 |  | PBANKA_041290 |  | circumsporozoite- and TRAP-related protein (CTRP) |
| 62 |  | 4 |  | PBANKA_082110 | ko | CS domain protein, putative |
| 63 |  | 5 |  | PBANKA_070190 |  | GPI-anchored micronemal antigen, putative (GAMA)(PSOP9) |
| 64 |  | 6 |  | PBANKA_131270 |  | gamete egress and sporozoite traversal protein (GEST) |
| 65 |  | 7 |  | PBANKA_146300 |  | osmiophilic body protein (G377) |
| 66 |  | 8 |  | PBANKA_082420 |  | perforin like protein 3 (PPLP3) |
| 67 |  | 9 |  | PBANKA_071140 | ko | perforin like protein 4 (PPLP4) |
| 68 |  | 10 |  | PBANKA_071160 |  | perforin like protein 5 (PPLP5) |
| 69 |  | 11 |  | PBANKA_103780 |  | secreted ookinete adhesive protein (SOAP) |
| 70 |  | 12 |  | PBANKA_061920 |  | secreted ookinete protein, putative (PSOP1) |
| 71 |  | 13 |  | PBANKA_111340 |  | secreted ookinete protein, putative (PSOP12) |
| 72 |  | 14 |  | PBANKA_114370 |  | secreted ookinete protein, putative (PSOP2) |
| 73 |  | 15 |  | PBANKA_112900 |  | secreted ookinete protein, putative (PSOP6) |
| 74 |  | 16 |  | PBANKA_135340 |  | secreted ookinete protein, putative (PSOP7) |
| 75 |  | 17 |  | PBANKA_110710 |  | subtilisin-like protease 1, putative (SUB1) |
| 76 |  | 18 |  | PBANKA_122890 |  | von willebrand factor a-domain-related protein (WARP) |
| 77 |  | 19 | POM1 | PBANKA_020170 |  | conserved Plasmodium protein, unknown function |
| 78 |  | 20 | POM2 | PBANKA_041720 | ko | conserved Plasmodium protein, unknown function |
| 79 |  | 21 | POM3 | PBANKA_050950 |  | conserved Plasmodium protein, unknown function |
| 80 |  | 22 | POM4 | PBANKA_052370 |  | conserved Plasmodium protein, unknown function |
| 81 |  | 23 | POM5 | PBANKA_070480 |  | conserved Plasmodium protein, unknown function |
| 82 |  | 24 | POM6 | PBANKA_070490 |  | conserved Plasmodium protein, unknown function |
| 83 |  | 25 | POM7 | PBANKA_080720 | ko | conserved Plasmodium protein, unknown function |
| 84 |  | 26 | POM8 | PBANKA_082120 |  | conserved Plasmodium protein, unknown function |
| 85 |  | 27 | POM9 | PBANKA_082150 |  | conserved Plasmodium protein, unknown function |
| 86 |  | 28 | POM10 | PBANKA_083080 |  | conserved Plasmodium protein, unknown function |
| 87 |  | 29 | POM11 | PBANKA_083370 |  | conserved Plasmodium protein, unknown function |
| 88 |  | 30 | POM12 | PBANKA_094110 |  | conserved Plasmodium protein, unknown function |
| 89 |  | 31 | POM13 | PBANKA_110690 |  | conserved Plasmodium protein, unknown function |
| 90 |  | 32 | POM14 | PBANKA_112320 |  | conserved Plasmodium protein, unknown function |
| 91 |  | 33 | POM15 | PBANKA_112330 |  | conserved Plasmodium protein, unknown function |
| 92 |  | 34 | POM16 | PBANKA_114380 | ko | conserved Plasmodium protein, unknown function |
| 93 |  | 35 | POM17 | PBANKA_120450 |  | conserved Plasmodium protein, unknown function |
| 94 |  | 36 | POM18 | PBANKA_123370 |  | conserved Plasmodium protein, unknown function |
| 95 |  | 37 | POM19 | PBANKA_123600 |  | conserved Plasmodium protein, unknown function |
| 96 |  | 38 | POM20 | PBANKA_124350 |  | conserved Plasmodium protein, unknown function |
| 97 |  | 39 | POM21 | PBANKA_134420 |  | conserved Plasmodium protein, unknown function |
| 98 |  | 40 | POM22 | PBANKA_135230 |  | conserved Plasmodium protein, unknown function |
| 99 |  | 41 | POM23 | PBANKA_135250 |  | conserved Plasmodium protein, unknown function |
| 100 |  | 42 | POM24 | PBANKA_136270 |  | conserved Plasmodium protein, unknown function |
| 101 |  | 43 | POM25 | PBANKA_145770 | ko | conserved Plasmodium protein, unknown function |
| 102 | vesicular protein transport | 1 |  | PBANKA_031400 |  | clathrin coat assembly protein, putative |
| 103 |  | 2 |  | PBANKA_122230 |  | coatomer epsilon subunit, putative |
| 104 |  | 3 |  | PBANKA_145910 |  | conserved Plasmodium protein, unknown function (vacuolar-sorting receptor) |
| 105 |  | 4 |  | PBANKA_021110 |  | conserved Plasmodium protein, unknown function (Vps 53-like) |
| 106 |  | 5 |  | PBANKA_050430 |  | flagellar outer arm dynein-associated protein, putative |
| 107 |  | 6 |  | PBANKA_060950 |  | kinesin-related protein, putative |
| 108 |  | 7 |  | PBANKA_080770 |  | kinesin-related protein, putative |
| 109 |  | 8 |  | PBANKA_031100 |  | protein transport protein Sec31, putative (SEC31) |
| 110 |  | 9 |  | PBANKA_134200 |  | Qa-SNARE protein, putative |
| 111 |  | 10 |  | PBANKA_111350 |  | Rab1a, putative |
| 112 |  | 11 |  | PBANKA_131230 |  | SNARE protein, putative |
| 113 |  | 12 |  | PBANKA_011200 |  | syntaxin binding protein, putative |
| 114 |  | 13 |  | PBANKA_094280 |  | syntaxin, putative |
| 115 |  | 14 |  | PBANKA_136130 |  | TBC domain protein, putative |
| 116 |  | 15 |  | PBANKA_123400 |  | vacuolar ATP synthetase, putative |
| 117 |  | 16 |  | PBANKA_031310 |  | vacuolar sorting protein VPS45, putative |
| 118 | putative surface protein | 1 |  | PBANKA_051500 |  | 25 kDa ookinete surface antigen precursor (P25) |
| 119 |  | 2 |  | PBANKA_051490 |  | 28 kDa ookinete surface protein (P28) |
| 120 |  | 3 |  | PBANKA_110760 | ko | 6-cysteine protein (P38) |
| 121 |  | 4 |  | PBANKA_111960 |  | merozoite surface protein 10, putative (MSP10) |
| 122 |  | 5 | POS1 | PBANKA_031370 |  | conserved Plasmodium protein, unknown function |
| 123 |  | 6 | POS2 | PBANKA_050550 |  | conserved Plasmodium protein, unknown function |
| 124 |  | 7 | POS3 | PBANKA_072090 |  | conserved Plasmodium protein, unknown function |
| 125 |  | 8 | POS4 | PBANKA_091650 |  | conserved Plasmodium protein, unknown function |
| 126 |  | 9 | POS5 | PBANKA_091710 |  | conserved Plasmodium protein, unknown function |
| 127 |  | 10 | POS6 | PBANKA_092560 |  | conserved Plasmodium protein, unknown function |
| 128 |  | 11 | POS7 | PBANKA_100640 | ko | conserved Plasmodium protein, unknown function |
| 129 |  | 12 | POS8 | PBANKA_111920 | ko | conserved Plasmodium protein, unknown function |
| 130 |  | 13 | POS9 | PBANKA_120070 | ko | conserved Plasmodium protein, unknown function |
| 131 |  | 14 | POS10 | PBANKA_135170 |  | conserved Plasmodium protein, unknown function |
| 132 | redox system | 1 |  | PBANKA_040310 |  | 1-cys-glutaredoxin-like protein-1, putative (GLP1) |
| 133 |  | 2 |  | PBANKA_061790 |  | ferrodoxin reductase-like protein, putative |
| 134 |  | 3 |  | PBANKA_082410 |  | flavodoxin-like protein |
| 135 |  | 4 |  | PBANKA_081980 | * | gamma-glutamylcysteine synthetase (gammaGCS) |
| 136 |  | 5 |  | PBANKA_102340 |  | glutathione reductase, putative (GR) |
| 137 |  | 6 |  | PBANKA_111180 |  | glutathione synthetase (GS) |
| 138 |  | 7 |  | PBANKA_114340 |  | NADH-cytochrome b5 reductase, putative |
| 139 |  | 8 |  | PBANKA_040220 |  | plasmoredoxin (Plrx) |
| 140 |  | 9 |  | PBANKA_082020 |  | thioredoxin, putative |
| 141 | oocyst development | 1 |  | PBANKA_123320 |  | cyclin, putative |
| 142 |  | 2 |  | PBANKA_103520 |  | LCCL domain-containing protein (CCp3) |
| 143 |  | 3 |  | PBANKA_121810 |  | oocyst capsule protein (Cap380) |
| 144 | protein kinase | 1 |  | PBANKA_031420 |  | calcium dependent protein kinase 1 (CDPK1) |
| 145 |  | 2 |  | PBANKA_040820 |  | calcium dependent protein kinase 3 (CDPK3) |
| 146 |  | 3 |  | PBANKA_113320 |  | cdc2-related kinase 2 (CRK2) |
| 147 |  | 4 |  | PBANKA_111190 |  | CDK-activating kinase assembly factor, putative (MAT1) |
| 148 |  | 5 |  | PBANKA_041040 |  | glycogen synthase kinase 3 (GSK3) |
| 149 |  | 6 |  | PBANKA_135090 |  | protein kinase 6 (PK6) |
| 150 |  | 7 |  | PBANKA_110320 |  | protein kinase, putative |
| 151 |  | 8 |  | PBANKA_134590 |  | protein kinase, putative |
| 152 |  | 9 |  | PBANKA_082960 |  | serine/threonine protein kinase, putative |
| 153 |  | 10 |  | PBANKA_146050 |  | serine/threonine protein kinase, putative |
| 154 | RNA-binding | 1 |  | PBANKA_112260 |  | ATP-dependent DEAD box helicase, putative |
| 155 |  | 2 |  | PBANKA_134750 |  | ATP-dependent RNA helicase, putative |
| 156 |  | 3 |  | PBANKA_061880 |  | DEAD box helicase, putative |
| 157 |  | 4 |  | PBANKA_123630 |  | DEAD-box subfamily ATP-dependent helicase, putative |
| 158 |  | 5 |  | PBANKA_123350 |  | mRNA-binding protein PUF1 (PUF1) |
| 159 |  | 6 |  | PBANKA_051380 |  | RAP protein, putative |
| 160 |  | 7 |  | PBANKA_144130 |  | RAP protein, putative |
| 161 |  | 8 |  | PBANKA_050570 |  | RNA helicase, putative |
| 162 |  | 9 |  | PBANKA_103620 |  | RNA-binding protein, putative |
| 163 |  | 10 |  | PBANKA_010390 |  | RNA-binding protein, putative |
| 164 |  | 11 |  | PBANKA_103630 |  | RNA-binding protein, putative |
| 165 |  | 12 |  | PBANKA_123270 |  | zinc finger protein, putative |
| 166 |  | 13 |  | PBANKA_113490 |  | zinc finger protein, putative |
| 167 |  | 14 |  | PBANKA_060860 |  | zinc finger protein, putative |
| 168 |  | 15 |  | PBANKA_120260 |  | zinc finger, C3HC4 type, putative |
| 169 | organella | 1 | apicoplast | PBANKA_134780 |  | 20 kDa chaperonin, putative (CPN20) |
| 170 |  | 2 |  | PBANKA_092910 |  | acyl-CoA-binding protein, putative |
| 171 |  | 3 |  | PBANKA_070320 |  | apicoplast ribosomal protein L21 precursor, putative |
| 172 |  | 4 |  | PBANKA_133820 |  | beta-hydroxyacyl-ACP dehydratase, putative (FabZ) |
| 173 |  | 5 |  | PBANKA_112190 |  | chorismate synthase, putative (CS) |
| 174 |  | 6 |  | PBANKA_050500 |  | dihydrolipoamide acyltransferase, putative |
| 175 |  | 7 |  | PBANKA_081970 |  | dihydrouridine synthase, putative |
| 176 |  | 8 |  | PBANKA_010120 |  | elongation factor G, putative (EF-G) |
| 177 |  | 9 |  | PBANKA_083480 |  | histidyl-tRNA synthetase, putative |
| 178 |  | 10 |  | PBANKA_081530 |  | met-10 like protein, putative |
| 179 |  | 11 |  | PBANKA_133070 |  | organelle ribosomal protein L22/L17 precursor, putative |
| 180 |  | 12 |  | PBANKA_080930 |  | peptide deformylase, putative |
| 181 |  | 13 |  | PBANKA_030570 |  | ribosome-recycling factor, putative (RRF) |
| 182 |  | 14 |  | PBANKA_050460 |  | S-adenosyl-L-methionine-dependent methyltransferase, putative |
| 183 |  | 15 |  | PBANKA_135260 |  | serine/threonine protein kinase, putative |
| 184 |  | 16 |  | PBANKA_020180 |  | TatD-like deoxyribonuclease, putative |
| 185 |  | 17 |  | PBANKA_145000 |  | tetQ family GTPase, putative |
| 186 |  | 18 |  | PBANKA_082520 |  | thiamin pyrophosphokinase, putative |
| 187 |  | 19 |  | PBANKA_083330 |  | zinc finger protein, putative |
| 188 |  | 20 | mitochondrion | PBANKA_110420 |  | 3-methyl-2-oxobutanoate dehydrogenase (lipoamide), putative |
| 189 |  | 21 |  | PBANKA_071760 |  | adenylate kinase 1, putative |
| 190 |  | 22 |  | PBANKA_145030 |  | ATP synthase subunit beta, mitochondrial, putative |
| 191 |  | 23 |  | PBANKA_101320 |  | ATP-specific succinyl-CoA synthetase beta subunit, putative |
| 192 |  | 24 |  | PBANKA_071870 |  | CDGSH iron-sulfur domain-containing protein, putative |
| 193 |  | 25 |  | PBANKA_101700 |  | CorA-like Mg2 transporter protein, putative |
| 194 |  | 26 |  | PBANKA_081910 |  | cytochrome b5, putative |
| 195 |  | 27 |  | PBANKA_122150 |  | mitochondrial ribosomal protein L1 precursor, putative |
| 196 |  | 28 |  | PBANKA_041330 |  | mitochondrial ribosomal protein L29/L47 precursor, putative |
| 197 |  | 29 |  | PBANKA_133960 |  | MSF1-like protein, putative |
| 198 | proteolysis (including ubiquitination) | 1 |  | PBANKA_112340 |  | anaphase-promoting complex subunit, putative |
| 199 |  | 2 |  | PBANKA_093130 |  | dipeptidyl aminopeptidase, putative |
| 200 |  | 3 |  | PBANKA_135080 |  | endopeptidase, putative |
| 201 |  | 4 |  | PBANKA_113140 |  | metacaspase 1 (MCA1) |
| 202 |  | 5 |  | PBANKA_082400 |  | OTU-like cysteine protease, putative |
| 203 |  | 6 |  | PBANKA_100650 |  | peptidase, M22 family, putative |
| 204 |  | 7 |  | PBANKA_092850 |  | peptidase, putative |
| 205 |  | 8 |  | PBANKA_092650 |  | petidase, M16 family, putative |
| 206 |  | 9 |  | PBANKA_040970 |  | plasmepsin VI |
| 207 |  | 10 |  | PBANKA_061030 |  | polyubiquitin, putative |
| 208 |  | 11 |  | PBANKA_122650 |  | proteasome beta-subunit, putative |
| 209 |  | 12 |  | PBANKA_080820 |  | proteasome component C8, putative |
| 210 |  | 13 |  | PBANKA_122290 |  | proteasome subunit alpha type 5, putative |
| 211 |  | 14 |  | PBANKA_134620 |  | signal peptidase 21 kDa subunit, putative (SP21) |
| 212 |  | 15 |  | PBANKA_090450 |  | ubiquitin activating enzyme (E1) subunit Aos1, putative |
| 213 |  | 16 |  | PBANKA_060280 |  | ubiquitin-conjugating enzyme e2, putative |
| 214 |  | 17 |  | PBANKA_020970 |  | zinc carboxy peptidase, putative |
| 215 | DNA replication and repair | 1 |  | PBANKA_101480 |  | ATP-dependent DNA helicase, putative |
| 216 |  | 2 |  | PBANKA_083490 |  | DNA excision-repair helicase, putative |
| 217 |  | 3 |  | PBANKA_021040 |  | DNA mismatch repair protein, putative |
| 218 |  | 4 |  | PBANKA_122680 |  | DNA repair protein rad54, putative |
| 219 |  | 5 |  | PBANKA_071830 |  | DNA replication licensing factor, putative |
| 220 |  | 6 |  | PBANKA_101910 |  | DNA-directed DNA polymerase, putative |
| 221 |  | 7 |  | PBANKA_101410 |  | exodeoxyribonuclease III, putative |
| 222 |  | 8 |  | PBANKA_111380 |  | helicase, putative (UIS26) |
| 223 |  | 9 |  | PBANKA_071400 |  | meiotic recombination protein DMC1-like protein, putative |
| 224 |  | 10 |  | PBANKA_031250 |  | origin recognition complex subunit 5, putative (ORC5) |
| 225 |  | 11 |  | PBANKA_020250 |  | replication factor c protein, putative |
| 226 |  | 12 |  | PBANKA_031470 |  | replication factor C, subunit 2, putative |
| 227 | transcription and translation | 1 |  | PBANKA_061750 |  | 3'-5' exoribonuclease Csl4 homolog, putative |
| 228 |  | 2 |  | PBANKA_131080 |  | 40S ribosomal protein S2, putative |
| 229 |  | 3 |  | PBANKA_071780 |  | 60S ribosomal protein L15, putative |
| 230 |  | 4 |  | PBANKA_136420 |  | 60S ribosomal protein L17, putative |
| 231 |  | 5 |  | PBANKA_101950 |  | 60S ribosomal protein L5, putative |
| 232 |  | 6 |  | PBANKA_101940 |  | 60S ribosomal protein L7-3, putative |
| 233 |  | 7 |  | PBANKA_021020 |  | aspartyl-tRNA synthetase, putative |
| 234 |  | 8 |  | PBANKA_103640 |  | BOP1-like protein, putative |
| 235 |  | 9 |  | PBANKA_123430 |  | carbon catabolite repressor protein 4, putative (CCR4) |
| 236 |  | 10 |  | PBANKA_070920 |  | clp1-related protein, putative |
| 237 |  | 11 |  | PBANKA_030320 |  | DNA-directed RNA polymerase II 16 kDa subunit, putative |
| 238 |  | 12 |  | PBANKA_081420 |  | elongation factor 1-beta, putative |
| 239 |  | 13 |  | PBANKA_134560 |  | elongation factor Tu, putative |
| 240 |  | 14 |  | PBANKA_141440 |  | exportin-T, putative |
| 241 |  | 15 |  | PBANKA_136200 |  | glutamate - tRNA ligase, putative |
| 242 |  | 16 |  | PBANKA_082650 |  | histone deacetylase, putative (HDAC1) |
| 243 |  | 17 |  | PBANKA_134770 |  | isoleucine - tRNA ligase, putative |
| 244 |  | 18 |  | PBANKA_083380 |  | mRNA processing protein, putative |
| 245 |  | 19 |  | PBANKA_061960 |  | PelOta protein homologue, putative |
| 246 |  | 20 |  | PBANKA_110830 |  | Pre-mRNA-processing ATP-dependent RNA helicase prp5, putative (PRP5) |
| 247 |  | 21 |  | PBANKA_123610 |  | ribosomal large subunit pseudouridylate synthase, putative |
| 248 |  | 22 |  | PBANKA_020350 |  | selenocysteine-specific elongation factor selB homologue, putative |
| 249 |  | 23 |  | PBANKA_110770 |  | SET domain protein, putative |
| 250 |  | 24 |  | PBANKA_092280 |  | small nuclear ribonucleoprotein D1, putative (SNRPD1) |
| 251 |  | 25 |  | PBANKA_070930 |  | small ribosomal subunit processing microtubule-associated protein, putative |
| 252 |  | 26 |  | PBANKA_083520 |  | transcription factor with AP2 domain(s), putative |
| 253 |  | 27 |  | PBANKA_052170 |  | transcription factor with AP2 domain, putative (ApiAP2) |
| 254 |  | 28 |  | PBANKA_051400 |  | transcription factor, putative |
| 255 |  | 29 |  | PBANKA_134760 |  | translation initiation factor 6, putative |
| 256 |  | 30 |  | PBANKA_050400 |  | tRNA methyltransferase, putative |
| 257 |  | 31 |  | PBANKA_122280 |  | tyrosyl-tRNA synthetase, putative |
| 258 |  | 32 |  | PBANKA_092880 |  | U2 snRNP auxiliary factor, small subunit, putative |
| 259 |  | 33 |  | PBANKA_093780 |  | U3/U14 snoRNA-associated small subunit rRNA processing protein, putative |
| 260 |  | 34 |  | PBANKA_121480 |  | U4/U6.U5 tri-snRNP-associated protein 1, putative (SART1) |
| 261 |  | 35 |  | PBANKA_134020 |  | U6 snRNA-associated Sm-like protein LSm6, putative (LSM6) |
| 262 | others | 1 |  | PBANKA_094180 |  | histone H2B, putative (H2B) |
| 263 |  | 2 |  | PBANKA_082620 |  | protein phosphatase-beta, putative |
| 264 |  | 3 |  | PBANKA_010130 |  | geranylgeranyltransferase, putative |
| 265 |  | 4 |  | PBANKA_134570 |  | GPI transamidase subunit PIG-U, putative |
| 266 |  | 5 |  | PBANKA_134840 |  | histone H3 variant, putative (CenH3) |
| 267 |  | 6 |  | PBANKA_010880 |  | histone H3, putative |
| 268 |  | 7 |  | PBANKA_111570 |  | Ndc80 homolog, putative |
| 269 |  | 8 |  | PBANKA_082570 |  | telomeric repeat binding factor 1, putative |
| 270 |  | 9 |  | PBANKA_134380 |  | transcriptional regulatory protein sir2a (Sir2A) |
| 271 |  | 10 |  | PBANKA_113810 |  | 14-3-3 protein, putative |
| 272 |  | 11 |  | PBANKA_100550 |  | AAA family ATPase, putative |
| 273 |  | 12 |  | PBANKA_061680 |  | actin-related protein |
| 274 |  | 13 |  | PBANKA_020930 |  | actin-related protein (ARP1) |
| 275 |  | 14 |  | PBANKA_111870 |  | acyl-CoA synthetase, putative |
| 276 |  | 15 |  | PBANKA_113110 |  | adenylosuccinate synthetase, putative |
| 277 |  | 16 |  | PBANKA_090600 |  | alpha/beta hydrolase, putative |
| 278 |  | 17 |  | PBANKA_040760 |  | asparagine synthetase, putative |
| 279 |  | 18 |  | PBANKA_091990 |  | ATP-dependent phosphofructokinase, putative |
| 280 |  | 19 |  | PBANKA_050410 |  | autophagy-related protein 8, putative (ATG8) |
| 281 |  | 20 |  | PBANKA_051100 |  | biotin--acetyl-CoA-carboxylase, putative |
| 282 |  | 21 |  | PBANKA_010420 |  | calcium-binding protein, putative |
| 283 |  | 22 |  | PBANKA_050580 |  | deoxyribose-phosphate aldolase, putative |
| 284 |  | 23 |  | PBANKA_134000 |  | dihydrofolate synthase/folylpolyglutamate synthase, putative |
| 285 |  | 24 |  | PBANKA_134530 |  | DNAJ like protein, putative |
| 286 |  | 25 |  | PBANKA_102110 |  | DnaJ protein, putative |
| 287 |  | 26 |  | PBANKA_030840 |  | dolichol-linked oligosaccharide biosynthesis enzyme, putative |
| 288 |  | 27 |  | PBANKA_040560 |  | EB1 homolog, putative |
| 289 |  | 28 |  | PBANKA_070650 |  | exonuclease, putative |
| 290 |  | 29 |  | PBANKA_142230 |  | FAD-dependent monooxygenase, putative |
| 291 |  | 30 |  | PBANKA_020430 |  | fatty acid elongation protein, GNS1/SUR4 family, putative, pseudogene |
| 292 |  | 31 |  | PBANKA_146040 |  | FK506-binding protein (FKBP)-type peptidyl-prolyl isomerase, putative (FKBP35) |
| 293 |  | 32 |  | PBANKA_122430 |  | glycosyltransferase, putative |
| 294 |  | 33 |  | PBANKA_071370 |  | GTPase, putative |
| 295 |  | 34 |  | PBANKA_093030 |  | GTP-binding nuclear protein, putative |
| 296 |  | 35 |  | PBANKA_031000 |  | heat shock 40 kDa protein, putative |
| 297 |  | 36 |  | PBANKA_060960 |  | heat shock protein 20, putative |
| 298 |  | 37 |  | PBANKA_081890 |  | heat shock protein 70, putative |
| 299 |  | 38 |  | PBANKA_071190 |  | heat shock protein, putative (HSP70) |
| 300 |  | 39 |  | PBANKA_030250 |  | hexose transporter (HT) |
| 301 |  | 40 |  | PBANKA_020340 |  | human hepatopoietin-like protein, putative |
| 302 |  | 41 |  | PBANKA_041410 |  | inorganic pyrophosphatase, putative |
| 303 |  | 42 |  | PBANKA_113080 |  | inositol-polyphosphate 5-phosphatase, putative |
| 304 |  | 43 |  | PBANKA_123130 |  | metabolite/drug transporter, putative |
| 305 |  | 44 |  | PBANKA_114240 |  | nucleoside diphosphate kinase b, putative |
| 306 |  | 45 |  | PBANKA_134720 |  | nucleotidyltransferase, putative |
| 307 |  | 46 |  | PBANKA_010740 |  | ornithine aminotransferase, putative (OAT) |
| 308 |  | 47 |  | PBANKA_050740 |  | orotidine-monophosphate-decarboxylase, putative (OMPDC) |
| 309 |  | 48 |  | PBANKA_080940 |  | P1 nuclease, putative |
| 310 |  | 49 |  | PBANKA_061140 |  | pantothenate kinase, putative |
| 311 |  | 50 |  | PBANKA_102260 |  | pantothenate kinase, putative |
| 312 |  | 51 |  | PBANKA_122720 |  | peptidyl-prolyl cis-trans isomerase, putative (CYP81) |
| 313 |  | 52 |  | PBANKA_010770 |  | permease, putative |
| 314 |  | 53 |  | PBANKA_101790 |  | phosphoenolpyruvate carboxylase (PEPC) |
| 315 |  | 54 |  | PBANKA_123080 |  | pyridoxal kinase-like protein, putative |
| 316 |  | 55 |  | PBANKA_070860 |  | quinone oxidoreductase, putative |
| 317 |  | 56 |  | PBANKA_092220 |  | ThiF family protein, putative |
| 318 |  | 57 |  | PBANKA_110490 |  | UDP-N-acetyl glucosamine:UMP antiporter, putative |
| 319 | unannotated genes | 1 |  | PBANKA_010450 |  | conserved Plasmodium protein, unknown function |
| 320 |  | 2 |  | PBANKA_010640 |  | conserved Plasmodium protein, unknown function |
| 321 |  | 3 |  | PBANKA_010650 |  | conserved Plasmodium protein, unknown function |
| 322 |  | 4 |  | PBANKA_010700 |  | conserved Plasmodium protein, unknown function |
| 323 |  | 5 |  | PBANKA_011160 |  | conserved Plasmodium protein, unknown function |
| 324 |  | 6 |  | PBANKA_020220 |  | conserved Plasmodium protein, unknown function |
| 325 |  | 7 |  | PBANKA_020240 |  | conserved Plasmodium protein, unknown function |
| 326 |  | 8 |  | PBANKA_020680 |  | conserved Plasmodium protein, unknown function |
| 327 |  | 9 |  | PBANKA_020850 |  | conserved Plasmodium protein, unknown function |
| 328 |  | 10 |  | PBANKA_020860 |  | conserved Plasmodium protein, unknown function |
| 329 |  | 11 |  | PBANKA_021000 |  | conserved Plasmodium protein, unknown function |
| 330 |  | 12 |  | PBANKA_021030 |  | conserved Plasmodium protein, unknown function |
| 331 |  | 13 |  | PBANKA_021290 |  | conserved Plasmodium protein, unknown function |
| 332 |  | 14 |  | PBANKA_030310 |  | conserved Plasmodium protein, unknown function |
| 333 |  | 15 |  | PBANKA_030450 |  | conserved Plasmodium protein, unknown function |
| 334 |  | 16 |  | PBANKA_030550 |  | conserved Plasmodium protein, unknown function |
| 335 |  | 17 |  | PBANKA_031150 |  | conserved Plasmodium protein, unknown function |
| 336 |  | 18 |  | PBANKA_031200 |  | conserved Plasmodium protein, unknown function |
| 337 |  | 19 |  | PBANKA_040170 |  | conserved Plasmodium protein, unknown function |
| 338 |  | 20 |  | PBANKA_040580 |  | conserved Plasmodium protein, unknown function |
| 339 |  | 21 |  | PBANKA_040590 |  | conserved Plasmodium protein, unknown function |
| 340 |  | 22 |  | PBANKA_040890 |  | conserved Plasmodium protein, unknown function |
| 341 |  | 23 |  | PBANKA_040900 |  | conserved Plasmodium protein, unknown function |
| 342 |  | 24 |  | PBANKA_041065 |  | conserved Plasmodium protein, unknown function |
| 343 |  | 25 |  | PBANKA_041420 |  | conserved Plasmodium protein, unknown function |
| 344 |  | 26 |  | PBANKA_041430 |  | conserved Plasmodium protein, unknown function |
| 345 |  | 27 |  | PBANKA_050300 |  | conserved Plasmodium protein, unknown function |
| 346 |  | 28 |  | PBANKA_050380 |  | conserved Plasmodium protein, unknown function |
| 347 |  | 29 |  | PBANKA_050440 |  | conserved Plasmodium protein, unknown function |
| 348 |  | 30 |  | PBANKA_050470 |  | conserved Plasmodium protein, unknown function |
| 349 |  | 31 |  | PBANKA_050530 |  | conserved Plasmodium protein, unknown function |
| 350 |  | 32 |  | PBANKA_050540 |  | conserved Plasmodium protein, unknown function |
| 351 |  | 33 |  | PBANKA_050600 |  | conserved Plasmodium protein, unknown function |
| 352 |  | 34 |  | PBANKA_050720 |  | conserved Plasmodium protein, unknown function |
| 353 |  | 35 |  | PBANKA_050820 |  | conserved Plasmodium protein, unknown function |
| 354 |  | 36 |  | PBANKA_050830 |  | conserved Plasmodium protein, unknown function |
| 355 |  | 37 |  | PBANKA_050860 |  | conserved Plasmodium protein, unknown function |
| 356 |  | 38 |  | PBANKA_050870 |  | conserved Plasmodium protein, unknown function |
| 357 |  | 39 |  | PBANKA_050880 |  | conserved Plasmodium protein, unknown function |
| 358 |  | 40 |  | PBANKA_050890 |  | conserved Plasmodium protein, unknown function |
| 359 |  | 41 |  | PBANKA_050960 |  | conserved Plasmodium protein, unknown function |
| 360 |  | 42 |  | PBANKA_051040 |  | conserved Plasmodium protein, unknown function |
| 361 |  | 43 |  | PBANKA_051050 |  | conserved Plasmodium protein, unknown function |
| 362 |  | 44 |  | PBANKA_051070 |  | conserved Plasmodium protein, unknown function |
| 363 |  | 45 |  | PBANKA_051530 |  | conserved Plasmodium protein, unknown function |
| 364 |  | 46 |  | PBANKA_051810 |  | conserved Plasmodium protein, unknown function |
| 365 |  | 47 |  | PBANKA_052230 |  | conserved Plasmodium protein, unknown function |
| 366 |  | 48 |  | PBANKA_060150 |  | conserved Plasmodium protein, unknown function |
| 367 |  | 49 |  | PBANKA_060300 |  | conserved Plasmodium protein, unknown function |
| 368 |  | 50 |  | PBANKA_060310 |  | conserved Plasmodium protein, unknown function |
| 369 |  | 51 |  | PBANKA_060350 |  | conserved Plasmodium protein, unknown function |
| 370 |  | 52 |  | PBANKA_060400 |  | conserved Plasmodium protein, unknown function |
| 371 |  | 53 |  | PBANKA_061070 |  | conserved Plasmodium protein, unknown function |
| 372 |  | 54 |  | PBANKA_061150 |  | conserved Plasmodium protein, unknown function |
| 373 |  | 55 |  | PBANKA_061240 |  | conserved Plasmodium protein, unknown function |
| 374 |  | 56 |  | PBANKA_061290 |  | conserved Plasmodium protein, unknown function |
| 375 |  | 57 |  | PBANKA_061300 |  | conserved Plasmodium protein, unknown function |
| 376 |  | 58 |  | PBANKA_061620 |  | conserved Plasmodium protein, unknown function |
| 377 |  | 59 |  | PBANKA_061780 |  | conserved Plasmodium protein, unknown function |
| 378 |  | 60 |  | PBANKA_061850 |  | conserved Plasmodium protein, unknown function |
| 379 |  | 61 |  | PBANKA_061870 |  | conserved Plasmodium protein, unknown function |
| 380 |  | 62 |  | PBANKA_062060 |  | conserved Plasmodium protein, unknown function |
| 381 |  | 63 |  | PBANKA_062070 |  | conserved Plasmodium protein, unknown function |
| 382 |  | 64 |  | PBANKA_062090 |  | conserved Plasmodium protein, unknown function |
| 383 |  | 65 |  | PBANKA_062100 |  | conserved Plasmodium protein, unknown function |
| 384 |  | 66 |  | PBANKA_062110 |  | conserved Plasmodium protein, unknown function |
| 385 |  | 67 |  | PBANKA_062150 |  | conserved Plasmodium protein, unknown function |
| 386 |  | 68 |  | PBANKA_062230 |  | conserved Plasmodium protein, unknown function |
| 387 |  | 69 |  | PBANKA_070660 |  | conserved Plasmodium protein, unknown function |
| 388 |  | 70 |  | PBANKA_070850 |  | conserved Plasmodium protein, unknown function |
| 389 |  | 71 |  | PBANKA_071250 |  | conserved Plasmodium protein, unknown function |
| 390 |  | 72 |  | PBANKA_071320 |  | conserved Plasmodium protein, unknown function |
| 391 |  | 73 |  | PBANKA_071680 |  | conserved Plasmodium protein, unknown function |
| 392 |  | 74 |  | PBANKA_071910 |  | conserved Plasmodium protein, unknown function |
| 393 |  | 75 |  | PBANKA_081030 |  | conserved Plasmodium protein, unknown function |
| 394 |  | 76 |  | PBANKA_081480 |  | conserved Plasmodium protein, unknown function |
| 395 |  | 77 |  | PBANKA_081620 |  | conserved Plasmodium protein, unknown function |
| 396 |  | 78 |  | PBANKA_081650 |  | conserved Plasmodium protein, unknown function |
| 397 |  | 79 |  | PBANKA_081690 |  | conserved Plasmodium protein, unknown function |
| 398 |  | 80 |  | PBANKA_081840 |  | conserved Plasmodium protein, unknown function |
| 399 |  | 81 |  | PBANKA_082130 |  | conserved Plasmodium protein, unknown function |
| 400 |  | 82 |  | PBANKA_082320 |  | conserved Plasmodium protein, unknown function |
| 401 |  | 83 |  | PBANKA_082500 |  | conserved Plasmodium protein, unknown function |
| 402 |  | 84 |  | PBANKA_082590 |  | conserved Plasmodium protein, unknown function |
| 403 |  | 85 |  | PBANKA_082950 |  | conserved Plasmodium protein, unknown function |
| 404 |  | 86 |  | PBANKA_082990 |  | conserved Plasmodium protein, unknown function |
| 405 |  | 87 |  | PBANKA_083240 |  | conserved Plasmodium protein, unknown function |
| 406 |  | 88 |  | PBANKA_083280 |  | conserved Plasmodium protein, unknown function |
| 407 |  | 89 |  | PBANKA_083420 |  | conserved Plasmodium protein, unknown function |
| 408 |  | 90 |  | PBANKA_083470 |  | conserved Plasmodium protein, unknown function |
| 409 |  | 91 |  | PBANKA_083590 |  | conserved Plasmodium protein, unknown function |
| 410 |  | 92 |  | PBANKA_090320 |  | conserved Plasmodium protein, unknown function |
| 411 |  | 93 |  | PBANKA_090610 |  | conserved Plasmodium protein, unknown function |
| 412 |  | 94 |  | PBANKA_090680 |  | conserved Plasmodium protein, unknown function |
| 413 |  | 95 |  | PBANKA_090700 |  | conserved Plasmodium protein, unknown function |
| 414 |  | 96 |  | PBANKA_090860 |  | conserved Plasmodium protein, unknown function |
| 415 |  | 97 |  | PBANKA_091540 |  | conserved Plasmodium protein, unknown function |
| 416 |  | 98 |  | PBANKA_091670 |  | conserved Plasmodium protein, unknown function |
| 417 |  | 99 |  | PBANKA_091690 |  | conserved Plasmodium protein, unknown function |
| 418 |  | 100 |  | PBANKA_091770 |  | conserved Plasmodium protein, unknown function |
| 419 |  | 101 |  | PBANKA_092030 |  | conserved Plasmodium protein, unknown function |
| 420 |  | 102 |  | PBANKA_092160 |  | conserved Plasmodium protein, unknown function |
| 421 |  | 103 |  | PBANKA_092250 |  | conserved Plasmodium protein, unknown function |
| 422 |  | 104 |  | PBANKA_092400 |  | conserved Plasmodium protein, unknown function |
| 423 |  | 105 |  | PBANKA_092450 |  | conserved Plasmodium protein, unknown function |
| 424 |  | 106 |  | PBANKA_092800 |  | conserved Plasmodium protein, unknown function |
| 425 |  | 107 |  | PBANKA_093010 |  | conserved Plasmodium protein, unknown function |
| 426 |  | 108 |  | PBANKA_093660 |  | conserved Plasmodium protein, unknown function |
| 427 |  | 109 |  | PBANKA_093670 |  | conserved Plasmodium protein, unknown function |
| 428 |  | 110 |  | PBANKA_093750 |  | conserved Plasmodium protein, unknown function |
| 429 |  | 111 |  | PBANKA_093880 |  | conserved Plasmodium protein, unknown function |
| 430 |  | 112 |  | PBANKA_094100 |  | conserved Plasmodium protein, unknown function |
| 431 |  | 113 |  | PBANKA_094170 |  | conserved Plasmodium protein, unknown function |
| 432 |  | 114 |  | PBANKA_100990 |  | conserved Plasmodium protein, unknown function |
| 433 |  | 115 |  | PBANKA_101260 |  | conserved Plasmodium protein, unknown function |
| 434 |  | 116 |  | PBANKA_101390 |  | conserved Plasmodium protein, unknown function |
| 435 |  | 117 |  | PBANKA_101580 |  | conserved Plasmodium protein, unknown function |
| 436 |  | 118 |  | PBANKA_101900 |  | conserved Plasmodium protein, unknown function |
| 437 |  | 119 |  | PBANKA_101920 |  | conserved Plasmodium protein, unknown function |
| 438 |  | 120 |  | PBANKA_101935 |  | conserved Plasmodium protein, unknown function |
| 439 |  | 121 |  | PBANKA_102050 |  | conserved Plasmodium protein, unknown function |
| 440 |  | 122 |  | PBANKA_102080 |  | conserved Plasmodium protein, unknown function |
| 441 |  | 123 |  | PBANKA_102100 |  | conserved Plasmodium protein, unknown function |
| 442 |  | 124 |  | PBANKA_102120 |  | conserved Plasmodium protein, unknown function |
| 443 |  | 125 |  | PBANKA_102380 |  | conserved Plasmodium protein, unknown function |
| 444 |  | 126 |  | PBANKA_103890 |  | conserved Plasmodium protein, unknown function |
| 445 |  | 127 |  | PBANKA_110390 |  | conserved Plasmodium protein, unknown function |
| 446 |  | 128 |  | PBANKA_110750 |  | conserved Plasmodium protein, unknown function |
| 447 |  | 129 |  | PBANKA_110910 |  | conserved Plasmodium protein, unknown function |
| 448 |  | 130 |  | PBANKA_110990 |  | conserved Plasmodium protein, unknown function |
| 449 |  | 131 |  | PBANKA_111000 |  | conserved Plasmodium protein, unknown function |
| 450 |  | 132 |  | PBANKA_111200 |  | conserved Plasmodium protein, unknown function |
| 451 |  | 133 |  | PBANKA_111610 |  | conserved Plasmodium protein, unknown function |
| 452 |  | 134 |  | PBANKA_111640 |  | conserved Plasmodium protein, unknown function |
| 453 |  | 135 |  | PBANKA_111680 |  | conserved Plasmodium protein, unknown function |
| 454 |  | 136 |  | PBANKA_111760 |  | conserved Plasmodium protein, unknown function |
| 455 |  | 137 |  | PBANKA_111820 |  | conserved Plasmodium protein, unknown function |
| 456 |  | 138 |  | PBANKA_111830 |  | conserved Plasmodium protein, unknown function |
| 457 |  | 139 |  | PBANKA_111840 |  | conserved Plasmodium protein, unknown function |
| 458 |  | 140 |  | PBANKA_111880 |  | conserved Plasmodium protein, unknown function |
| 459 |  | 141 |  | PBANKA_111980 |  | conserved Plasmodium protein, unknown function |
| 460 |  | 142 |  | PBANKA_112010 |  | conserved Plasmodium protein, unknown function |
| 461 |  | 143 |  | PBANKA_112100 |  | conserved Plasmodium protein, unknown function |
| 462 |  | 144 |  | PBANKA_112250 |  | conserved Plasmodium protein, unknown function |
| 463 |  | 145 |  | PBANKA_112310 |  | conserved Plasmodium protein, unknown function |
| 464 |  | 146 |  | PBANKA_112470 |  | conserved Plasmodium protein, unknown function |
| 465 |  | 147 |  | PBANKA_112950 |  | conserved Plasmodium protein, unknown function |
| 466 |  | 148 |  | PBANKA_113150 |  | conserved Plasmodium protein, unknown function |
| 467 |  | 149 |  | PBANKA_114140 |  | conserved Plasmodium protein, unknown function |
| 468 |  | 150 |  | PBANKA_114160 |  | conserved Plasmodium protein, unknown function |
| 469 |  | 151 |  | PBANKA_114190 |  | conserved Plasmodium protein, unknown function |
| 470 |  | 152 |  | PBANKA_120460 |  | conserved Plasmodium protein, unknown function |
| 471 |  | 153 |  | PBANKA_120470 |  | conserved Plasmodium protein, unknown function |
| 472 |  | 154 |  | PBANKA_120970 |  | conserved Plasmodium protein, unknown function |
| 473 |  | 155 |  | PBANKA_122080 |  | conserved Plasmodium protein, unknown function |
| 474 |  | 156 |  | PBANKA_122170 |  | conserved Plasmodium protein, unknown function |
| 475 |  | 157 |  | PBANKA_122180 |  | conserved Plasmodium protein, unknown function |
| 476 |  | 158 |  | PBANKA_122270 |  | conserved Plasmodium protein, unknown function |
| 477 |  | 159 |  | PBANKA_122320 |  | conserved Plasmodium protein, unknown function |
| 478 |  | 160 |  | PBANKA_122610 |  | conserved Plasmodium protein, unknown function |
| 479 |  | 161 |  | PBANKA_122730 |  | conserved Plasmodium protein, unknown function |
| 480 |  | 162 |  | PBANKA_122780 |  | conserved Plasmodium protein, unknown function |
| 481 |  | 163 |  | PBANKA_122790 |  | conserved Plasmodium protein, unknown function |
| 482 |  | 164 |  | PBANKA_122830 |  | conserved Plasmodium protein, unknown function |
| 483 |  | 165 |  | PBANKA_122910 |  | conserved Plasmodium protein, unknown function |
| 484 |  | 166 |  | PBANKA_123060 |  | conserved Plasmodium protein, unknown function |
| 485 |  | 167 |  | PBANKA_123070 |  | conserved Plasmodium protein, unknown function |
| 486 |  | 168 |  | PBANKA_123120 |  | conserved Plasmodium protein, unknown function |
| 487 |  | 169 |  | PBANKA_123200 |  | conserved Plasmodium protein, unknown function |
| 488 |  | 170 |  | PBANKA_123280 |  | conserved Plasmodium protein, unknown function |
| 489 |  | 171 |  | PBANKA_123440 |  | conserved Plasmodium protein, unknown function |
| 490 |  | 172 |  | PBANKA_123570 |  | conserved Plasmodium protein, unknown function |
| 491 |  | 173 |  | PBANKA_123580 |  | conserved Plasmodium protein, unknown function |
| 492 |  | 174 |  | PBANKA_124140 |  | conserved Plasmodium protein, unknown function |
| 493 |  | 175 |  | PBANKA_124260 |  | conserved Plasmodium protein, unknown function |
| 494 |  | 176 |  | PBANKA_124320 |  | conserved Plasmodium protein, unknown function |
| 495 |  | 177 |  | PBANKA_131280 |  | conserved Plasmodium protein, unknown function |
| 496 |  | 178 |  | PBANKA_131310 |  | conserved Plasmodium protein, unknown function |
| 497 |  | 179 |  | PBANKA_131860 |  | conserved Plasmodium protein, unknown function |
| 498 |  | 180 |  | PBANKA_133080 |  | conserved Plasmodium protein, unknown function |
| 499 |  | 181 |  | PBANKA_133660 |  | conserved Plasmodium protein, unknown function |
| 500 |  | 182 |  | PBANKA_133680 |  | conserved Plasmodium protein, unknown function |
| 501 |  | 183 |  | PBANKA_133930 |  | conserved Plasmodium protein, unknown function |
| 502 |  | 184 |  | PBANKA_133950 |  | conserved Plasmodium protein, unknown function |
| 503 |  | 185 |  | PBANKA_134250 |  | conserved Plasmodium protein, unknown function |
| 504 |  | 186 |  | PBANKA_134550 |  | conserved Plasmodium protein, unknown function |
| 505 |  | 187 |  | PBANKA_134730 |  | conserved Plasmodium protein, unknown function |
| 506 |  | 188 |  | PBANKA_134820 |  | conserved Plasmodium protein, unknown function |
| 507 |  | 189 |  | PBANKA_134850 |  | conserved Plasmodium protein, unknown function |
| 508 |  | 190 |  | PBANKA_134870 |  | conserved Plasmodium protein, unknown function |
| 509 |  | 191 |  | PBANKA_135310 |  | conserved Plasmodium protein, unknown function |
| 510 |  | 192 |  | PBANKA_135380 |  | conserved Plasmodium protein, unknown function |
| 511 |  | 193 |  | PBANKA_135950 |  | conserved Plasmodium protein, unknown function |
| 512 |  | 194 |  | PBANKA_136360 |  | conserved Plasmodium protein, unknown function |
| 513 |  | 195 |  | PBANKA_136410 |  | conserved Plasmodium protein, unknown function |
| 514 |  | 196 |  | PBANKA_140440 |  | conserved Plasmodium protein, unknown function |
| 515 |  | 197 |  | PBANKA_140690 |  | conserved Plasmodium protein, unknown function |
| 516 |  | 198 |  | PBANKA_140820 |  | conserved Plasmodium protein, unknown function |
| 517 |  | 199 |  | PBANKA_140920 |  | conserved Plasmodium protein, unknown function |
| 518 |  | 200 |  | PBANKA_141740 |  | conserved Plasmodium protein, unknown function |
| 519 |  | 201 |  | PBANKA_142280 |  | conserved Plasmodium protein, unknown function |
| 520 |  | 202 |  | PBANKA_142430 |  | conserved Plasmodium protein, unknown function |
| 521 |  | 203 |  | PBANKA_143520 |  | conserved Plasmodium protein, unknown function |
| 522 |  | 204 |  | PBANKA_143770 |  | conserved Plasmodium protein, unknown function |
| 523 |  | 205 |  | PBANKA_143780 |  | conserved Plasmodium protein, unknown function |
| 524 |  | 206 |  | PBANKA_144120 |  | conserved Plasmodium protein, unknown function |
| 525 |  | 207 |  | PBANKA_144360 |  | conserved Plasmodium protein, unknown function |
| 526 |  | 208 |  | PBANKA_144370 |  | conserved Plasmodium protein, unknown function |
| 527 |  | 209 |  | PBANKA_144460 |  | conserved Plasmodium protein, unknown function |
| 528 |  | 210 |  | PBANKA_144470 |  | conserved Plasmodium protein, unknown function |
| 529 |  | 211 |  | PBANKA_144880 |  | conserved Plasmodium protein, unknown function |
| 530 |  | 212 |  | PBANKA_145080 |  | conserved Plasmodium protein, unknown function |
| 531 |  | 213 |  | PBANKA_145670 |  | conserved Plasmodium protein, unknown function |
| 532 |  | 214 |  | PBANKA_145980 |  | conserved Plasmodium protein, unknown function |
| 533 |  | 215 |  | PBANKA_146100 |  | conserved Plasmodium protein, unknown function |
| 534 |  | 216 |  | PBANKA_146120 |  | conserved Plasmodium protein, unknown function |
| 535 |  | 217 |  | PBANKA_146130 |  | conserved Plasmodium protein, unknown function |
| 536 |  | 218 |  | PBANKA_146390 |  | conserved Plasmodium protein, unknown function |
| 537 |  | 219 |  | PBANKA_071900 |  | conserved Plasmodium protein, unknown function, fragment |
| 538 |  | 220 |  | PBANKA_090230 |  | conserved Plasmodium protein, unknown function, fragment |
| 539 |  | 221 |  | PBANKA_091700 |  | conserved Plasmodium protein, unknown function, fragment |
| 540 |  | 222 |  | PBANKA_031060 |  | conserved protein, unknown function |
| 541 |  | 223 |  | PBANKA_020090 |  | conserved rodent malaria protein, unknown function |
| 542 |  | 224 |  | PBANKA_122220 |  | conserved rodent malaria protein, unknown function |
| 543 |  | 225 |  | PBANKA_020370 |  | conserved Plasmodium membrane protein, unknown function |

^a^ Target genes in Table S1 were classified into several categories according to their putative functions, localizations, and structures. They were arranged according to the order they were mentioned in the text. Asterisks indicate genes that have peaks over 1200-bp upstream.

**Table S8. Comparison of the predicted target genes with microneme proteome**

|  | Gene ID^a^ | functional annotation | AP2-O target | microneme^b^ |
| --- | --- | --- | --- | --- |
| 1 | PBANKA_051500 | 25 kDa ookinete surface antigen precursor (P25) | Yes |  |
| 2 | PBANKA_051490 | 28 kDa ookinete surface protein (P28) | Yes |  |
| 3 | PBANKA_131080 | 40S ribosomal protein S2, putative | Yes |  |
| 4 | PBANKA_071780 | 60S ribosomal protein L15, putative | Yes |  |
| 5 | PBANKA_136420 | 60S ribosomal protein L17, putative | Yes |  |
| 6 | PBANKA_101950 | 60S ribosomal protein L5, putative | Yes |  |
| 7 | PBANKA_110760 | 6-cysteine protein (P38) | Yes |  |
| 8 | PBANKA_110310 | actin-depolymerizing factor 1 (ADF1) | Yes |  |
| 9 | PBANKA_145030 | ATP synthase subunit beta, mitochondrial, putative | Yes |  |
| 10 | PBANKA_101320 | ATP-specific succinyl-CoA synthetase beta subunit, putative | Yes |  |
| 11 | PBANKA_031420 | calcium dependent protein kinase 1 (CDPK1) | Yes |  |
| 12 | PBANKA_143230 | cell traversal protein for ookinetes and sporozoites (CelTOS) | Yes | microneme |
| 13 | PBANKA_080050 | chitinase (CHT1) | Yes | microneme |
| 14 | PBANKA_041290 | circumsporozoite- and TRAP-related protein (CTRP) | Yes | microneme |
| 15 | PBANKA_145770 | conserved Plasmodium protein, unknown function | Yes |  |
| 16 | PBANKA_142430 | conserved Plasmodium protein, unknown function | Yes |  |
| 17 | PBANKA_140920 | conserved Plasmodium protein, unknown function | Yes |  |
| 18 | PBANKA_135460 | conserved Plasmodium protein, unknown function | Yes |  |
| 19 | PBANKA_134250 | conserved Plasmodium protein, unknown function | Yes |  |
| 20 | PBANKA_122540 | conserved Plasmodium protein, unknown function | Yes |  |
| 21 | PBANKA_114380 | conserved Plasmodium protein, unknown function | Yes |  |
| 22 | PBANKA_111680 | conserved Plasmodium protein, unknown function | Yes |  |
| 23 | PBANKA_094110 | conserved Plasmodium protein, unknown function | Yes |  |
| 24 | PBANKA_091670 | conserved Plasmodium protein, unknown function | Yes |  |
| 25 | PBANKA_083040 | conserved Plasmodium protein, unknown function | Yes |  |
| 26 | PBANKA_070660 | conserved Plasmodium protein, unknown function | Yes |  |
| 27 | PBANKA_041720 | conserved Plasmodium protein, unknown function | Yes |  |
| 28 | PBANKA_093130 | dipeptidyl aminopeptidase, putative | Yes |  |
| 29 | PBANKA_131270 | gamete egress and sporozoite traversal protein (GEST) | Yes |  |
| 30 | PBANKA_111530 | glideosome-associated protein 40, putative (GAP40) | Yes |  |
| 31 | PBANKA_102340 | glutathione reductase, putative (GR) | Yes |  |
| 32 | PBANKA_070190 | GPI-anchored micronemal antigen, putative (GAMA) | Yes | microneme |
| 33 | PBANKA_093030 | GTP-binding nuclear protein, putative | Yes |  |
| 34 | PBANKA_031000 | heat shock 40 kDa protein, putative | Yes |  |
| 35 | PBANKA_081890 | heat shock protein 70, putative | Yes |  |
| 36 | PBANKA_071190 | heat shock protein, putative (HSP70) | Yes |  |
| 37 | PBANKA_030250 | hexose transporter (HT) | Yes |  |
| 38 | PBANKA_094180 | histone H2B, putative (H2B) | Yes |  |
| 39 | PBANKA_134840 | histone H3 variant, putative (CenH3) | Yes |  |
| 40 | PBANKA_103520 | LCCL domain-containing protein (CCp3) | Yes |  |
| 41 | PBANKA_120200 | membrane skeletal protein, putative | Yes |  |
| 42 | PBANKA_070710 | membrane skeletal protein, putative | Yes |  |
| 43 | PBANKA_145950 | myosin light chain 1, putative,myosin A tail domain interacting protein MTIP, putative (MTIP) | Yes |  |
| 44 | PBANKA_146300 | osmiophilic body protein (G377) | Yes |  |
| 45 | PBANKA_071140 | perforin like protein 4 (PPLP4) | Yes | microneme |
| 46 | PBANKA_061030 | polyubiquitin, putative | Yes |  |
| 47 | PBANKA_031100 | protein transport protein Sec31, putative (SEC31) | Yes |  |
| 48 | PBANKA_103780 | secreted ookinete adhesive protein (SOAP) | Yes | microneme |
| 49 | PBANKA_061920 | secreted ookinete protein, putative (PSOP1) | Yes | microneme |
| 50 | PBANKA_111340 | secreted ookinete protein, putative (PSOP12) | Yes | microneme |
| 51 | PBANKA_114370 | secreted ookinete protein, putative (PSOP2) | Yes | microneme |
| 52 | PBANKA_135340 | secreted ookinete protein, putative (PSOP7) | Yes | microneme |
| 53 | PBANKA_071430 | small heat shock protein HSP20 (HSP20) | Yes |  |
| 54 | PBANKA_092280 | small nuclear ribonucleoprotein D1, putative (SNRPD1) | Yes |  |
| 55 | PBANKA_081070 | subpellicular microtubule protein 1, putative (SPM1) | Yes |  |
| 56 | PBANKA_094280 | syntaxin, putative | Yes |  |
| 57 | PBANKA_082020 | thioredoxin, putative | Yes |  |
| 58 | PBANKA_122890 | von willebrand factor a-domain-related protein (WARP) | Yes | microneme |
| 59 | PBANKA_071260 | 14-3-3 protein, putative | No |  |
| 60 | PBANKA_120660 | 26S proteasome regulatory subunit 4, putative | No |  |
| 61 | PBANKA_141000 | 26S proteasome regulatory subunit 7, putative | No |  |
| 62 | PBANKA_132970 | 26S proteasome subunit, putative | No |  |
| 63 | PBANKA_113510 | 40S ribosomal protein S13, putative | No |  |
| 64 | PBANKA_123100 | 40S ribosomal protein S14, putative | No |  |
| 65 | PBANKA_141630 | 40S ribosomal protein S15/S19, putative | No |  |
| 66 | PBANKA_142360 | 40S ribosomal protein S16, putative | No |  |
| 67 | PBANKA_145610 | 40S ribosomal protein S17, putative | No |  |
| 68 | PBANKA_092210 | 40S ribosomal protein S18, putative | No |  |
| 69 | PBANKA_052280 | 40S ribosomal protein S19, putative | No |  |
| 70 | PBANKA_040530 | 40S ribosomal protein S23, putative | No |  |
| 71 | PBANKA_123420 | 40S ribosomal protein S24, putative | No |  |
| 72 | PBANKA_102200 | 40S ribosomal protein S25, putative | No |  |
| 73 | PBANKA_031450 | 40S ribosomal protein S26e, putative | No |  |
| 74 | PBANKA_132930 | 40S ribosomal protein S3, putative | No |  |
| 75 | PBANKA_121560 | 40S ribosomal protein S3A, putative | No |  |
| 76 | PBANKA_094150 | 40S ribosomal protein S4, putative | No |  |
| 77 | PBANKA_135510 | 40S ribosomal protein S6, putative | No |  |
| 78 | PBANKA_140130 | 40S ribosomal protein S7, putative | No |  |
| 79 | PBANKA_103390 | 40S ribosomal protein S8e, putative | No |  |
| 80 | PBANKA_123480 | 40S ribosomal protein S9, putative | No |  |
| 81 | PBANKA_144680 | 60 kDa chaperonin, putative (CPN60) | No |  |
| 82 | PBANKA_040770 | 60S acidic ribosomal protein P2, putative | No |  |
| 83 | PBANKA_094360 | 60S acidic ribosomal protein, putative | No |  |
| 84 | PBANKA_102840 | 60S ribosomal protein L10, putative | No |  |
| 85 | PBANKA_130510 | 60S ribosomal protein L10a, putative | No |  |
| 86 | PBANKA_142350 | 60S ribosomal protein L13-2, putative | No |  |
| 87 | PBANKA_101310 | 60S ribosomal protein L14, putative | No |  |
| 88 | PBANKA_135440 | 60S ribosomal protein L18, putative | No |  |
| 89 | PBANKA_135450 | 60S ribosomal protein L18-2, putative | No |  |
| 90 | PBANKA_122920 | 60S ribosomal protein L19, putative | No |  |
| 91 | PBANKA_134670 | 60S ribosomal protein L23, putative | No |  |
| 92 | PBANKA_140760 | 60S ribosomal protein L24, putative | No |  |
| 93 | PBANKA_132440 | 60S ribosomal protein L27, putative | No |  |
| 94 | PBANKA_090650 | 60S ribosomal protein L28, putative | No |  |
| 95 | PBANKA_051190 | 60S ribosomal protein L3, putative | No |  |
| 96 | PBANKA_050360 | 60S ribosomal protein L30e, putative | No |  |
| 97 | PBANKA_110340 | 60S ribosomal protein L31, putative | No |  |
| 98 | PBANKA_041750 | 60S ribosomal protein L32, putative | No |  |
| 99 | PBANKA_090640 | 60S ribosomal protein L35ae, putative | No |  |
| 100 | PBANKA_091810 | 60S ribosomal protein L38e, putative | No |  |
| 101 | PBANKA_110670 | 60S ribosomal protein L4, putative | No |  |
| 102 | PBANKA_114170 | 60S ribosomal protein L40/UBI, putative | No |  |
| 103 | PBANKA_135190 | 60S ribosomal protein L6-2, putative | No |  |
| 104 | PBANKA_040550 | 60S ribosomal protein L7, putative | No |  |
| 105 | PBANKA_123170 | 60S ribosomal protein L8, putative | No |  |
| 106 | PBANKA_091800 | 60S ribosomal protein, putative | No |  |
| 107 | PBANKA_135970 | 6-cysteine protein (P47) | No |  |
| 108 | PBANKA_145930 | actin I | No |  |
| 109 | PBANKA_145330 | acyl-CoA synthetase, putative | No |  |
| 110 | PBANKA_123990 | acyl-CoA synthetase, putative | No |  |
| 111 | PBANKA_133740 | adenosine-diphosphatase, putative | No |  |
| 112 | PBANKA_120720 | adenylate kinase, putative (AK1) | No |  |
| 113 | PBANKA_041770 | alpha tubulin 1 | No |  |
| 114 | PBANKA_131810 | aminopeptidase P, putative (APP) | No |  |
| 115 | PBANKA_091500 | apical membrane antigen 1 (AMA1) | No | other stage |
| 116 | PBANKA_130820 | apicoplast 1-acyl-sn-glycerol-3-phosphate acyltransferase, putative | No |  |
| 117 | PBANKA_093770 | apicoplast ribosomal protein L36e precursor, putative | No |  |
| 118 | PBANKA_144650 | asparagine-rich protein, putative | No |  |
| 119 | PBANKA_135770 | aspartate carbamoyltransferase, putative | No |  |
| 120 | PBANKA_132810 | ATP synthase (C/AC39) subunit, putative | No |  |
| 121 | PBANKA_031380 | ATP synthase F1, alpha subunit, putative | No |  |
| 122 | PBANKA_061040 | ATPase, putative | No |  |
| 123 | PBANKA_132830 | ATP-dependent protease la, putative | No |  |
| 124 | PBANKA_121770 | ATP-dependent RNA Helicase (DOZI) | No |  |
| 125 | PBANKA_030680 | ATP-dependent RNA helicase UAP56, putative (UAP56) | No |  |
| 126 | PBANKA_041670 | bacterial histone-like protein, putative (HU) | No |  |
| 127 | PBANKA_141110 | branched-chain alpha keto-acid dehydrogenase, putative | No |  |
| 128 | PBANKA_061520 | calcium dependent protein kinase 4 (CDPK4) | No |  |
| 129 | PBANKA_020700 | calcium-transporting ATPase, putative (SERCA) | No |  |
| 130 | PBANKA_145260 | calcyclin binding protein, putative | No |  |
| 131 | PBANKA_143800 | CAMP-dependent protein kinase regulatory subunit, putative (PKAr) | No |  |
| 132 | PBANKA_140670 | carbamoyl phosphate synthetase, putative | No |  |
| 133 | PBANKA_122160 | cell division cycle ATPase, putative | No |  |
| 134 | PBANKA_111860 | cell division cycle protein 48 homologue, putative | No |  |
| 135 | PBANKA_131040 | centrin, putative | No |  |
| 136 | PBANKA_020630 | centrin, putative | No |  |
| 137 | PBANKA_071600 | chromosome associated protein, putative | No |  |
| 138 | PBANKA_143470 | clathrin heavy chain, putative | No |  |
| 139 | PBANKA_071420 | ClpB protein, putative | No |  |
| 140 | PBANKA_131740 | co-chaperone p23, putative | No |  |
| 141 | PBANKA_145250 | conserved Plasmodium protein, unknown function | No |  |
| 142 | PBANKA_144950 | conserved Plasmodium protein, unknown function | No |  |
| 143 | PBANKA_144900 | conserved Plasmodium protein, unknown function | No |  |
| 144 | PBANKA_144480 | conserved Plasmodium protein, unknown function | No |  |
| 145 | PBANKA_142630 | conserved Plasmodium protein, unknown function | No |  |
| 146 | PBANKA_141480 | conserved Plasmodium protein, unknown function | No |  |
| 147 | PBANKA_133880 | conserved Plasmodium protein, unknown function | No |  |
| 148 | PBANKA_132600 | conserved Plasmodium protein, unknown function | No |  |
| 149 | PBANKA_121470 | conserved Plasmodium protein, unknown function | No |  |
| 150 | PBANKA_121170 | conserved Plasmodium protein, unknown function | No |  |
| 151 | PBANKA_114280 | conserved Plasmodium protein, unknown function | No |  |
| 152 | PBANKA_113780 | conserved Plasmodium protein, unknown function | No |  |
| 153 | PBANKA_113500 | conserved Plasmodium protein, unknown function | No |  |
| 154 | PBANKA_103060 | conserved Plasmodium protein, unknown function | No |  |
| 155 | PBANKA_101970 | conserved Plasmodium protein, unknown function | No |  |
| 156 | PBANKA_101280 | conserved Plasmodium protein, unknown function | No |  |
| 157 | PBANKA_101000 | conserved Plasmodium protein, unknown function | No |  |
| 158 | PBANKA_100790 | conserved Plasmodium protein, unknown function | No |  |
| 159 | PBANKA_094250 | conserved Plasmodium protein, unknown function | No |  |
| 160 | PBANKA_092820 | conserved Plasmodium protein, unknown function | No |  |
| 161 | PBANKA_092480 | conserved Plasmodium protein, unknown function | No |  |
| 162 | PBANKA_092320 | conserved Plasmodium protein, unknown function | No |  |
| 163 | PBANKA_091140 | conserved Plasmodium protein, unknown function | No |  |
| 164 | PBANKA_090620 | conserved Plasmodium protein, unknown function | No |  |
| 165 | PBANKA_082670 | conserved Plasmodium protein, unknown function | No |  |
| 166 | PBANKA_081850 | conserved Plasmodium protein, unknown function | No |  |
| 167 | PBANKA_081670 | conserved Plasmodium protein, unknown function | No |  |
| 168 | PBANKA_081540 | conserved Plasmodium protein, unknown function | No |  |
| 169 | PBANKA_081350 | conserved Plasmodium protein, unknown function | No |  |
| 170 | PBANKA_081340 | conserved Plasmodium protein, unknown function | No |  |
| 171 | PBANKA_080150 | conserved Plasmodium protein, unknown function | No |  |
| 172 | PBANKA_071220 | conserved Plasmodium protein, unknown function | No |  |
| 173 | PBANKA_061860 | conserved Plasmodium protein, unknown function | No |  |
| 174 | PBANKA_052300 | conserved Plasmodium protein, unknown function | No |  |
| 175 | PBANKA_051990 | conserved Plasmodium protein, unknown function | No |  |
| 176 | PBANKA_051790 | conserved Plasmodium protein, unknown function | No |  |
| 177 | PBANKA_030130 | conserved Plasmodium protein, unknown function | No |  |
| 178 | PBANKA_020500 | conserved Plasmodium protein, unknown function | No |  |
| 179 | PBANKA_020360 | conserved Plasmodium protein, unknown function | No |  |
| 180 | PBANKA_010530 | conserved Plasmodium protein, unknown function | No |  |
| 181 | PBANKA_114580 | conserved rodent malaria protein, unknown function | No |  |
| 182 | PBANKA_094340 | CPW-WPC family protein, putative | No |  |
| 183 | PBANKA_132170 | cysteine proteinase, putative | No |  |
| 184 | PBANKA_140060 | cytoadherence linked asexual protein, putative | No |  |
| 185 | PBANKA_144700 | cytochrome b5, putative | No |  |
| 186 | PBANKA_103800 | cytochrome c, putative | No |  |
| 187 | PBANKA_070670 | dicarboxylate/tricarboxylate carrier, putative (DTC) | No |  |
| 188 | PBANKA_141910 | dihydrolipamide succinyltransferase component of 2-oxoglutarate dehydrogenase, putative | No |  |
| 189 | PBANKA_144690 | dihydrolipoamide dehydrogenase, putative (mLipDH) | No |  |
| 190 | PBANKA_142330 | DNA/RNA-binding protein Alba 1, putative (ALBA1) | No |  |
| 191 | PBANKA_120440 | DNA/RNA-binding protein Alba 3, putative (ALBA3) | No |  |
| 192 | PBANKA_136030 | DNA/RNA-binding protein Alba 4, putative (ALBA4) | No |  |
| 193 | PBANKA_133640 | DnaJ protein, putative | No |  |
| 194 | PBANKA_112780 | DnaJ protein, putative | No |  |
| 195 | PBANKA_091180 | DnaJ protein, putative | No |  |
| 196 | PBANKA_070680 | DnaJ protein, putative | No |  |
| 197 | PBANKA_141730 | DnaJ/SEC63 protein, putative | No |  |
| 198 | PBANKA_082000 | DNAJ-like molecular chaperone protein, putative | No |  |
| 199 | PBANKA_120910 | dolichyl-phosphate-mannose protein mannosyltransferase, putative | No |  |
| 200 | PBANKA_090360 | dynamin-like protein, putative | No |  |
| 201 | PBANKA_051700 | early transcribed membrane protein (ETRAMP) | No | other stage |
| 202 | PBANKA_020160 | early transcribed membrane protein (ETRAMP) | No | other stage |
| 203 | PBANKA_052480 | early transcribed membrane protein (SEP1) | No | other stage |
| 204 | PBANKA_052420 | early transcribed membrane protein (SEP2) | No | other stage |
| 205 | PBANKA_091230 | EBNA2 binding protein P100 homologue, putative | No |  |
| 206 | PBANKA_040280 | EH (Eps15 homology) protein, putative | No |  |
| 207 | PBANKA_121890 | elongation factor 1 (EF-1), putative | No |  |
| 208 | PBANKA_131480 | elongation factor 2, putative | No |  |
| 209 | PBANKA_093840 | endoplasmic reticulum-resident calcium binding protein, putative | No |  |
| 210 | PBANKA_143730 | endoplasmin homolog precursor, putative | No |  |
| 211 | PBANKA_121430 | enolase, putative (ENO) | No |  |
| 212 | PBANKA_093160 | ER membrane protein Sec12, putative (SEC12) | No |  |
| 213 | PBANKA_100060 | erythrocyte membrane antigen 1 | No | other stage |
| 214 | PBANKA_080120 | erythrocyte membrane-associated antigen, putative | No | other stage |
| 215 | PBANKA_133190 | eukaryotic initiation factor 4a, putative (eIF4A) | No |  |
| 216 | PBANKA_141130 | eukaryotic translation initation factor 4 gamma, putative (EIF4G) | No |  |
| 217 | PBANKA_103190 | eukaryotic translation initiation factor 2 gamma subunit, putative | No |  |
| 218 | PBANKA_120900 | eukaryotic translation initiation factor 2, beta, putative | No |  |
| 219 | PBANKA_060320 | eukaryotic translation initiation factor 5a, putative (EIF5A) | No |  |
| 220 | PBANKA_071520 | eukaryotic translation initiation factor, putative | No |  |
| 221 | PBANKA_123220 | FACT complex subunit SPT16 (FACT-L) | No |  |
| 222 | PBANKA_040480 | FAD-dependent glycerol-3-phosphate dehydrogenase, putative | No |  |
| 223 | PBANKA_113700 | falcilysin, putative (FLN) | No |  |
| 224 | PBANKA_051820 | flavoprotein subunit of succinate dehydrogenase, putative | No |  |
| 225 | PBANKA_130860 | fructose-bisphosphate aldolase 2 (ALDO2) | No |  |
| 226 | PBANKA_132640 | glyceraldehyde-3-phosphate dehydrogenase, putative | No |  |
| 227 | PBANKA_142850 | glycerol-3-phosphate acyltransferase, putative | No |  |
| 228 | PBANKA_102660 | GTP-binding protein, putative | No |  |
| 229 | PBANKA_093120 | heat shock protein 101, putative | No |  |
| 230 | PBANKA_121400 | heat shock protein 60, putative | No |  |
| 231 | PBANKA_135720 | heat shock protein 70 (hsp70), putative | No |  |
| 232 | PBANKA_080570 | heat shock protein 90, putative (HSP90) | No |  |
| 233 | PBANKA_091440 | heat shock protein hsp70 homologue, putative (UIS24) | No |  |
| 234 | PBANKA_093830 | heat shock protein, putative | No |  |
| 235 | PBANKA_060190 | high mobility group protein, putative (HMGB1) | No |  |
| 236 | PBANKA_071290 | high mobility group protein, putative (HMGB2) | No |  |
| 237 | PBANKA_121760 | histone H2A variant, putative (H2A.Z) | No |  |
| 238 | PBANKA_111700 | histone H2A, putative (H2A) | No |  |
| 239 | PBANKA_142060 | histone H2B, putative | No |  |
| 240 | PBANKA_061090 | HSP40, subfamily A, putative | No |  |
| 241 | PBANKA_041450 | HVA22/TB2/DP1 family protein, putative | No |  |
| 242 | PBANKA_143660 | inner membrane complex protein 1h (IMC1h) | No |  |
| 243 | PBANKA_136550 | intra-erythrocytic P. berghei-induced structures protein 1 (IBIS1) | No | other stage |
| 244 | PBANKA_135860 | isocitrate dehydrogenase (NADP), mitochondrial precursor, putative (IDH) | No |  |
| 245 | PBANKA_123880 | karyopherin beta, putative | No |  |
| 246 | PBANKA_130070 | LCCL domain-containing protein (CCp1) | No |  |
| 247 | PBANKA_020450 | LCCL domain-containing protein (CCp5) | No |  |
| 248 | PBANKA_131950 | LCCL domain-containing protein CCP2 (CCP2) | No |  |
| 249 | PBANKA_134010 | L-lactate dehydrogenase (LDH) | No |  |
| 250 | PBANKA_136290 | lysine -- tRNA ligase, putative | No |  |
| 251 | PBANKA_141030 | M1-family aminopeptidase, putative | No | microneme |
| 252 | PBANKA_143220 | male development gene 1 (MDV1) | No |  |
| 253 | PBANKA_040270 | membrane skeletal protein, putative | No |  |
| 254 | PBANKA_083100 | merozoite surface protein 1 (MSP1) | No | other stage |
| 255 | PBANKA_134910 | merozoite surface protein 7 (MSP7) | No | other stage |
| 256 | PBANKA_110220 | merozoite surface protein 8 (MSP8) | No | other stage |
| 257 | PBANKA_144330 | merozoite surface protein 9, putative | No | other stage |
| 258 | PBANKA_090140 | mitochondrial ATP synthase delta subunit, putative | No |  |
| 259 | PBANKA_111650 | mitochondrial import receptor subunit, putative | No |  |
| 260 | PBANKA_123790 | mitochondrial processing peptidase alpha subunit, putative | No |  |
| 261 | PBANKA_030900 | mitochondrial ribosomal protein L12 precursor, putative | No |  |
| 262 | PBANKA_134900 | MSP7-like protein (MSRP2) | No | other stage |
| 263 | PBANKA_031330 | MtN3-like protein | No |  |
| 264 | PBANKA_123780 | multidrug resistance protein, putative (MDR1) | No |  |
| 265 | PBANKA_092000 | multiprotein bridging factor type 1, putative | No |  |
| 266 | PBANKA_135570 | myosin A (MyoA) | No |  |
| 267 | PBANKA_061180 | N-acetyltransferase, putative | No |  |
| 268 | PBANKA_100950 | NAD(P)H-dependent glutamate synthase, putative | No |  |
| 269 | PBANKA_040160 | N-ethylmaleimide-sensitive fusion protein, putative | No |  |
| 270 | PBANKA_081990 | nucleosome assembly protein (NAPS) | No |  |
| 271 | PBANKA_060270 | nucleosome assembly protein 1, putative (NAPL) | No |  |
| 272 | PBANKA_020920 | parasite-infected erythrocyte surface protein (PIESP15) | No |  |
| 273 | PBANKA_093220 | peptidyl-prolyl cis-trans isomerase, putative | No |  |
| 274 | PBANKA_121650 | peptidyl-prolyl cis-trans isomerase, putative (CYP19A) | No |  |
| 275 | PBANKA_100480 | peptidyl-tRNA hydrolase, putative | No |  |
| 276 | PBANKA_112040 | Pfs77 homologue, putative | No |  |
| 277 | PBANKA_082340 | phosphoglycerate kinase, putative (PGK) | No |  |
| 278 | PBANKA_081520 | phospholipid or glycerol acyltransferase, putative | No |  |
| 279 | PBANKA_134030 | phosphoribosylpyrophosphate synthetase, putative | No |  |
| 280 | PBANKA_103440 | plasmepsin IV (PM4) | No |  |
| 281 | PBANKA_122250 | plasmepsin X | No |  |
| 282 | PBANKA_143920 | polyadenylate-binding protein, putative (PABP) | No |  |
| 283 | PBANKA_093960 | polyadenylate-binding protein-interacting protein 1, putative (PAIP1) | No |  |
| 284 | PBANKA_030360 | proteasome 26S regulatory subunit, putative | No |  |
| 285 | PBANKA_070280 | protein disulfide isomerase | No |  |
| 286 | PBANKA_091430 | protein disulfide isomerase related protein, putative | No | microneme |
| 287 | PBANKA_112560 | pyruvate kinase, putative | No |  |
| 288 | PBANKA_121020 | QF122 antigen, putative | No |  |
| 289 | PBANKA_141890 | Rab GTPase 11a (Rab11a) | No |  |
| 290 | PBANKA_111230 | Rab1b, putative | No |  |
| 291 | PBANKA_041820 | Rab7, putative | No |  |
| 292 | PBANKA_070390 | receptor for activated C kinase, putative (RACK) | No |  |
| 293 | PBANKA_031600 | replication factor C subunit 1, putative | No |  |
| 294 | PBANKA_041600 | RhopH3, putative | No | other stage |
| 295 | PBANKA_080450 | rhoptry associated membrane antigen, putative (RAMA) | No | other stage |
| 296 | PBANKA_071310 | rhoptry neck protein 5, putative (RON5) | No | other stage |
| 297 | PBANKA_083020 | rhoptry protein-2, putative | No | other stage |
| 298 | PBANKA_061970 | rhoptry-associated leucine zipper-like protein 1, putative (RALP1) | No | other stage |
| 299 | PBANKA_103210 | rhoptry-associated protein 1, putative (RAP1) | No | other stage |
| 300 | PBANKA_110140 | rhoptry-associated protein 2/3 (RAP2/3) | No | other stage |
| 301 | PBANKA_111750 | ribosomal protein L27a, putative | No |  |
| 302 | PBANKA_103270 | RNA binding protein Bruno, putative (HoBo) | No |  |
| 303 | PBANKA_030690 | Sec61-gamma subunit of protein translocation complex, putative | No |  |
| 304 | PBANKA_081900 | secreted acid phosphatase, putative,glideosome-associated protein 50, putative (GAP50) | No |  |
| 305 | PBANKA_020480 | secreted ookinete protein, putative (PSOP24) | No | microneme |
| 306 | PBANKA_110300 | Ser/Arg-rich splicing factor, putative | No |  |
| 307 | PBANKA_145020 | serine hydroxymethyltransferase, putative (SHMT) | No |  |
| 308 | PBANKA_131380 | signal recognition particle 54 kDa protein, putative | No |  |
| 309 | PBANKA_071880 | small GTP-binding protein sar1, putative (SAR1) | No |  |
| 310 | PBANKA_131550 | sortilin, putative | No |  |
| 311 | PBANKA_092440 | splicing factor, putative | No |  |
| 312 | PBANKA_040520 | T-complex protein beta subunit, putative | No |  |
| 313 | PBANKA_091620 | TCP-1/cpn60 chaperonin family, putative | No |  |
| 314 | PBANKA_040650 | TCP-1/cpn60 chaperonin, putative | No |  |
| 315 | PBANKA_130280 | thioredoxin peroxidase 1 (TPx1) | No |  |
| 316 | PBANKA_136520 | thioredoxin-related protein, putative | No |  |
| 317 | PBANKA_130130 | trailer hitch homolog, putative (CITH) | No |  |
| 318 | PBANKA_101850 | transcription factor 3b, putative | No |  |
| 319 | PBANKA_120590 | transcription factor with AP2 domain(s), putative (ApiAP2) | No |  |
| 320 | PBANKA_145690 | translation initiation factor SUI1, putative | No |  |
| 321 | PBANKA_130220 | translocation protein sec62, putative | No |  |
| 322 | PBANKA_100850 | translocon component PTEX150 (PTEX150) | No | other stage |
| 323 | PBANKA_120690 | tubulin beta chain, putative | No |  |
| 324 | PBANKA_060760 | U6 snRNA-associated Sm-like protein LSm7, putative (LSM7) | No |  |
| 325 | PBANKA_082300 | ubiquitin-like protein, putative | No |  |
| 326 | PBANKA_141040 | vacuolar ATP synthase subunit a, putative | No |  |
| 327 | PBANKA_083530 | vacuolar ATP synthase subunit e, putative | No |  |
| 328 | PBANKA_133840 | vacuolar ATP synthase subunit g, putative | No |  |
| 329 | PBANKA_122380 | vacuolar proton-translocating ATPase subunit A, putative | No |  |
| 330 | PBANKA_130370 | vesicle-associated membrane protein, putative | No |  |

^a^ Old gene IDs attributed to microneme proteome were replaced with new ones according to recent annotation in PlasmoDB. By this process, redundant genes were removed and the number of proteins was reduced from 345 to 330.

^b^ Microneme proteins (microneme) and putative contaminants from other stages (other stage) are indicated.

1. [↑](#footnote-ref-1)
