# Supplementary material for: Genome-Wide Identification of the Target Genes of AP2-O, a Plasmodium AP2-Family Transcription Factor
Source: PLoS Pathog. 2015 May 27;11(5):e1004905. doi: 10.1371/journal.ppat.1004905 (PMC4446032; doi:10.1371/journal.ppat.1004905)
Supplement: S2 File — (DOCX) [file ppat.1004905.s010.docx]

**Table S9. Conversion rates to ookinetes in mutant parasites^a^**

| genotype | Female gametocyte  /20 fields | Ookinetes/20 fields | conversion rate(mean%±SE) |
| --- | --- | --- | --- |
| Wild-types | 136 | 246 | 62.7 (1.28) |
| Wild-types | 123 | 214 |  |
| Wild-types | 170 | 257 |  |
|  |  |  |  |
| *IMC1i*(–) clone1 | 106 | 139 | 56.4 (0.71) |
| *IMC1i*(–) clone1 | 86 | 116 |  |
| *IMC1i*(–) clone1 | 67 | 82 |  |
|  |  |  |  |
| *IAAP*(–) clone1 | 108 | 192 | 63.7 (0.19) |
| *IAAP*(–) clone1 | 128 | 224 |  |
| *IAAP*(–) clone1 | 133 | 230 |  |
|  |  |  |  |
| *PPLP4*(–) clone1 | 121 | 302 | 67.9 (1.94) |
| *PPLP4*(–) clone1 | 90 | 165 |  |
| *PPLP4*(–) clone1 | 97 | 202 |  |
|  |  |  |  |
| *POS8*(–) clone1 | 87 | 180 | 66.1 (1.16) |
| *POS8*(–) clone1 | 108 | 190 |  |
| *POS8*(–) clone1 | 128 | 260 |  |
|  |  |  |  |
| *CYC3*(–) clone1 | 98 | 295 | 71.8 (1.98) |
| *CYC3*(–) clone1 | 104 | 270 |  |
| *CYC3*(–) clone1 | 95 | 204 |  |

^a^ Three independent experiments were performed in each clone. The number of female gametocytes and ookinetes were counted under a microscope at × 100 magnification. The values are means of 20 fields.

**Table S10. Oocyst formation of *IMC1i*(–) parasites**^a^

| Genotype | oocyst ± SE | oocyst sporozoites |
| --- | --- | --- |
| Wild-types | 95.8±16.8 | 10758 |
| *IMC1i*(–)1 | 1.6±0.38 | 380 |
| *IMC1i*(–)2 | 3.3±0.82 | 367 |

^a^ Two independently prepared mutants were used for assessing the phenotype. The number of oocysts is an average from 20 mosquitoes. SE, standard error

**Table S11. Oocyst formation of *POM*(–) parasites**^a^

| Genotype | oocyst ± SE |
| --- | --- |
| Wild-types | 77.0±31.2 |
| *POM2*(–) | 90.0±20.6 |
| *POM7*(–) | 84.2±23.7 |
| *CS domain protein* (–) | 62.7±20.7 |
| *POM*16(–) | 47.1±10.7 |

^a^ Two independently prepared mutants were used for assessing the phenotype. The number of oocysts is an average from 20 mosquitoes. SE, standard error

**Table S12. Oocyst formation of *POS* (−) parasites**^a^

| Genotype | oocyst ± SE |
| --- | --- |
| Wild-types | 84.9±36.1 |
| *POS7*(−) | 108.4±32.9 |
| *POS9*(−) | 51.9±14.1 |

^a^ Two independently prepared mutants were used for assessing the phenotype. The number of oocysts is an average from 20 mosquitoes. SE, standard error

**Table S13. Top 500 genes most expressed in *P. berghei* ookinetes**

RNA-seq analysis was performed in *P. berghei* ookinetes cultured for 24 h, and genes were ordered according to reads per kilobase of coding sequence per million reads (RPKM) value.

|  | Gene ID | chromosome | Read  number | RPKM | Functional ID | target |
| --- | --- | --- | --- | --- | --- | --- |
| 1 | PBANKA_103780 | berg10 | 2258914 | 272814 | secreted ookinete adhesive protein (SOAP) | Yes |
| 2 | PBANKA_051500 | berg05 | 642864.3 | 59476.6 | 25 kDa ookinete surface antigen precursor (P25) | Yes |
| 3 | PBANKA_051490 | berg05 | 618563.1 | 58297.98 | 28 kDa ookinete surface protein (P28) | Yes |
| 4 | PBANKA_094180 | berg09 | 137737.3 | 23344.68 | histone H2B, putative (H2B) | Yes |
| 5 | PBANKA_020170 | berg02 | 92509.78 | 21695.64 | conserved Plasmodium protein, unknown function | Yes |
| 6 | PBANKA_122890 | berg12 | 310341 | 20589.64 | von willebrand factor a-domain-related protein (WARP) | Yes |
| 7 | PBANKA_143230 | berg14 | 181667.6 | 19699.16 | cell traversal protein for ookinetes and sporozoites (CelTOS) | Yes |
| 8 | PBANKA_122540 | berg12 | 87143.96 | 18119.61 | conserved Plasmodium protein, unknown function | Yes |
| 9 | PBANKA_111920 | berg11 | 305767.2 | 17569.81 | conserved Plasmodium protein, unknown function | Yes |
| 10 | PBANKA_145950 | berg14 | 167515.8 | 16481.06 | myosin light chain 1, putative,myosin A tail domain interacting protein MTIP, putative (MTIP) | Yes |
| 11 | PBANKA_094190 | berg09 | 75135.05 | 14571.11 | histone H4, putative | Yes |
| 12 | PBANKA_102440 | berg10 | 85909.06 | 12647.41 | calmodulin, putative | Yes |
| 13 | PBANKA_120990 | berg12 | 98548.63 | 11293.31 | conserved Plasmodium protein, unknown function | No |
| 14 | PBANKA_071290 | berg07 | 53214.33 | 10841.18 | high mobility group protein, putative (HMGB2) | No |
| 15 | PBANKA_111700 | berg11 | 56724.55 | 8537.87 | histone H2A, putative (H2A) | No |
| 16 | PBANKA_080050 | berg08 | 244687.6 | 7604.15 | chitinase (CHT1) | Yes |
| 17 | PBANKA_082020 | berg08 | 110003.7 | 7226.9 | thioredoxin, putative | Yes |
| 18 | PBANKA_121760 | berg12 | 109577.6 | 7075.98 | histone H2A variant, putative (H2A.Z) | No |
| 19 | PBANKA_140920 | berg14 | 53734.08 | 6992 | conserved Plasmodium protein, unknown function | Yes |
| 20 | PBANKA_040770 | berg04 | 38095.81 | 6860.29 | 60S acidic ribosomal protein P2, putative | No |
| 21 | PBANKA_142060 | berg14 | 41865.76 | 6809.58 | histone H2B, putative | No |
| 22 | PBANKA_131860 | berg13 | 45066.53 | 5902.24 | conserved Plasmodium protein, unknown function | Yes |
| 23 | PBANKA_133680 | berg13 | 30564.35 | 5504.03 | conserved Plasmodium protein, unknown function | Yes |
| 24 | PBANKA_135450 | berg13 | 109680.4 | 5395.46 | 60S ribosomal protein L18-2, putative | No |
| 25 | PBANKA_134900 | berg13 | 90094.56 | 5313.19 | MSP7-like protein (MSRP2) | No |
| 26 | PBANKA_100640 | berg10 | 77228.71 | 4792.68 | conserved Plasmodium protein, unknown function | Yes |
| 27 | PBANKA_140070 | berg14 | 53297.26 | 4721.6 | conserved rodent malaria protein, unknown function | No |
| 28 | PBANKA_092670 | berg09 | 56276.6 | 4690.25 | circumsporozoite-related antigen | No |
| 29 | PBANKA_111530 | berg11 | 106079.5 | 4661.24 | glideosome-associated protein 40, putative (GAP40) | Yes |
| 30 | PBANKA_111710 | berg11 | 31579.61 | 4649.11 | histone H3, putative (H3.3) | No |
| 31 | PBANKA_081900 | berg08 | 86840.17 | 4422.92 | secreted acid phosphatase, putative,glideosome-associated protein 50, putative (GAP50) | No |
| 32 | PBANKA_132500 | berg13 | 14837.37 | 4400.8 | 40S ribosomal protein S28e, putative | No |
| 33 | PBANKA_010880 | berg01 | 29226.16 | 4302.64 | histone H3, putative | Yes |
| 34 | PBANKA_020160 | berg02 | 37021.3 | 4194.84 | early transcribed membrane protein (ETRAMP) | No |
| 35 | PBANKA_031060 | berg03 | 36314.82 | 4016.99 | conserved protein, unknown function | Yes |
| 36 | PBANKA_101850 | berg10 | 34595.77 | 3987.2 | transcription factor 3b, putative | No |
| 37 | PBANKA_031450 | berg03 | 21289.55 | 3975.81 | 40S ribosomal protein S26e, putative | No |
| 38 | PBANKA_143760 | berg14 | 34205.11 | 3729.09 | glideosome-associated protein 45, putative | No |
| 39 | PBANKA_020460 | berg02 | 38836.94 | 3512.56 | photosensitized INA-labeled protein 1, putative | Yes |
| 40 | PBANKA_094360 | berg09 | 36879.43 | 3391.27 | 60S acidic ribosomal protein, putative | No |
| 41 | PBANKA_123400 | berg12 | 69064.28 | 3361.92 | vacuolar ATP synthetase, putative | No |
| 42 | PBANKA_091810 | berg09 | 13592.49 | 3343.25 | 60S ribosomal protein L38e, putative | No |
| 43 | PBANKA_040540 | berg04 | 34005.63 | 3180.18 | 40S ribosomal protein S12, putative | No |
| 44 | PBANKA_145670 | berg14 | 21403.7 | 3135.76 | conserved Plasmodium protein, unknown function | Yes |
| 45 | PBANKA_030130 | berg03 | 46692.9 | 3128.73 | conserved Plasmodium protein, unknown function | No |
| 46 | PBANKA_101770 | berg10 | 13075.77 | 2963.2 | ubiquinol-cytochrome c reductase hinge protein, putative | No |
| 47 | PBANKA_146130 | berg14 | 42252.76 | 2959.01 | conserved Plasmodium protein, unknown function | Yes |
| 48 | PBANKA_071680 | berg07 | 15637.21 | 2920.24 | conserved Plasmodium protein, unknown function | Yes |
| 49 | PBANKA_134670 | berg13 | 43217.62 | 2899.07 | 60S ribosomal protein L23, putative | No |
| 50 | PBANKA_061780 | berg06 | 18495.68 | 2869.53 | conserved Plasmodium protein, unknown function | Yes |
| 51 | PBANKA_110310 | berg11 | 25935.27 | 2827.5 | actin-depolymerizing factor 1 (ADF1) | Yes |
| 52 | PBANKA_123320 | berg12 | 30462.92 | 2805.5 | cyclin, putative | Yes |
| 53 | PBANKA_051070 | berg05 | 9489.71 | 2734.25 | conserved Plasmodium protein, unknown function | Yes |
| 54 | PBANKA_123900 | berg12 | 36951.35 | 2733.26 | conserved Plasmodium protein, unknown function | No |
| 55 | PBANKA_133890 | berg13 | 52433.26 | 2670.51 | glideosome associated protein with multiple membrane spans 1, putative (GAPM1) | Yes |
| 56 | PBANKA_103540 | berg10 | 53985.18 | 2662.16 | glideosome associated protein with multiple membrane spans 3, putative (GAPM3) | No |
| 57 | PBANKA_020410 | berg02 | 28436.64 | 2643.03 | N-terminal acetyltransferase, putative | No |
| 58 | PBANKA_061910 | berg06 | 32598.29 | 2545.06 | 40S ribosomal protein S5, putative | No |
| 59 | PBANKA_030690 | berg03 | 10524.79 | 2527.07 | Sec61-gamma subunit of protein translocation complex, putative | No |
| 60 | PBANKA_050180 | berg05 | 31120.96 | 2432.86 | conserved Plasmodium protein, unknown function | No |
| 61 | PBANKA_102840 | berg10 | 25502.85 | 2338.03 | 60S ribosomal protein L10, putative | No |
| 62 | PBANKA_060190 | berg06 | 12119.07 | 2305.93 | high mobility group protein, putative (HMGB1) | No |
| 63 | PBANKA_092120 | berg09 | 14061.42 | 2221.44 | conserved Plasmodium protein, unknown function | Yes |
| 64 | PBANKA_052280 | berg05 | 34624.89 | 2180.07 | 40S ribosomal protein S19, putative | No |
| 65 | PBANKA_031580 | berg03 | 16884.97 | 2164.53 | ribosome associated membrane protein RAMP4, putative | No |
| 66 | PBANKA_071430 | berg07 | 32265.96 | 2072.52 | small heat shock protein HSP20 (HSP20) | Yes |
| 67 | PBANKA_113510 | berg11 | 15540.88 | 2062.13 | 40S ribosomal protein S13, putative | No |
| 68 | PBANKA_114100 | berg11 | 5309.17 | 2059.24 | conserved Plasmodium protein, unknown function | No |
| 69 | PBANKA_123100 | berg12 | 24477.5 | 2045.66 | 40S ribosomal protein S14, putative | No |
| 70 | PBANKA_144970 | berg14 | 10763.35 | 2041.56 | conserved Plasmodium protein, unknown function | No |
| 71 | PBANKA_120190 | berg12 | 28321.79 | 1927.63 | 40S ribosomal protein S20e, putative | No |
| 72 | PBANKA_062190 | berg06 | 13705.7 | 1884.75 | mitochondrial import inner membrane translocase subunit tim14, putative | No |
| 73 | PBANKA_122230 | berg12 | 29146.01 | 1876.1 | coatomer epsilon subunit, putative | No |
| 74 | PBANKA_091560 | berg09 | 23911.28 | 1862.03 | aquaglyceroporin (AQP) | No |
| 75 | PBANKA_061720 | berg06 | 28207.62 | 1861.24 | 40S ribosomal protein S10, putative | No |
| 76 | PBANKA_090500 | berg09 | 14683.27 | 1843.23 | 40S ribosomal protein S21e, putative | No |
| 77 | PBANKA_071190 | berg07 | 61709.5 | 1793.39 | heat shock protein, putative (HSP70) | Yes |
| 78 | PBANKA_141630 | berg14 | 32187.73 | 1785.13 | 40S ribosomal protein S15/S19, putative | No |
| 79 | PBANKA_083040 | berg08 | 27659.12 | 1780.39 | conserved Plasmodium protein, unknown function | Yes |
| 80 | PBANKA_041460 | berg04 | 14877.27 | 1765.05 | 40S ribosomal protein S15A, putative | No |
| 81 | PBANKA_111750 | berg11 | 17853.07 | 1753.63 | ribosomal protein L27a, putative | No |
| 82 | PBANKA_061650 | berg06 | 15047.64 | 1705.03 | conserved Plasmodium protein, unknown function | Yes |
| 83 | PBANKA_090860 | berg09 | 18597.52 | 1702.39 | conserved Plasmodium protein, unknown function | Yes |
| 84 | PBANKA_124140 | berg12 | 11894.06 | 1689.37 | conserved Plasmodium protein, unknown function | No |
| 85 | PBANKA_080040 | berg08 | 9894.8 | 1686.49 | conserved Plasmodium protein, unknown function | No |
| 86 | PBANKA_123810 | berg12 | 19720.94 | 1671.22 | conserved Plasmodium protein, unknown function | No |
| 87 | PBANKA_121650 | berg12 | 13773.56 | 1624.55 | peptidyl-prolyl cis-trans isomerase, putative (CYP19A) | No |
| 88 | PBANKA_062020 | berg06 | 11892.05 | 1620.61 | conserved Plasmodium protein, unknown function | No |
| 89 | PBANKA_083300 | berg08 | 32211 | 1610.73 | profilin, putative (PFN) | No |
| 90 | PBANKA_060320 | berg06 | 12815.99 | 1595.59 | eukaryotic translation initiation factor 5a, putative (EIF5A) | No |
| 91 | PBANKA_120240 | berg12 | 25266.27 | 1580.95 | 60S ribosomal protein L13, putative | No |
| 92 | PBANKA_090470 | berg09 | 10670.39 | 1567.07 | 60S ribosomal protein L41, putative | No |
| 93 | PBANKA_040440 | berg04 | 10467.26 | 1540.98 | conserved Plasmodium protein, unknown function | No |
| 94 | PBANKA_135050 | berg13 | 9301.85 | 1537.78 | conserved Plasmodium protein, unknown function | No |
| 95 | PBANKA_132090 | berg13 | 13169 | 1529.4 | thioredoxin, putative (TRX1) | No |
| 96 | PBANKA_091800 | berg09 | 23903.81 | 1525.68 | 60S ribosomal protein, putative | No |
| 97 | PBANKA_102200 | berg10 | 19933.31 | 1517.11 | 40S ribosomal protein S25, putative | No |
| 98 | PBANKA_030700 | berg03 | 20779.94 | 1487.96 | 60S ribosomal protein L37ae, putative | No |
| 99 | PBANKA_082650 | berg08 | 32997.47 | 1485.55 | histone deacetylase, putative (HDAC1) | Yes |
| 100 | PBANKA_101060 | berg10 | 18057.51 | 1460.7 | calmodulin, putative | No |
| 101 | PBANKA_052230 | berg05 | 5425.15 | 1439.73 | conserved Plasmodium protein, unknown function | No |
| 102 | PBANKA_080400 | berg08 | 6569.5 | 1424.73 | ribosomal protein, L37e, putative | No |
| 103 | PBANKA_124260 | berg12 | 8785.35 | 1417.53 | conserved Plasmodium protein, unknown function | Yes |
| 104 | PBANKA_112340 | berg11 | 6021.89 | 1396.04 | anaphase-promoting complex subunit, putative | Yes |
| 105 | PBANKA_103470 | berg10 | 24167.75 | 1376.94 | nucleolar preribosomal GTPase, putative | No |
| 106 | PBANKA_120170 | berg12 | 10254 | 1351.72 | N-acetyltransferase, putative | No |
| 107 | PBANKA_061660 | berg06 | 21077.48 | 1350.99 | conserved Plasmodium protein, unknown function | No |
| 108 | PBANKA_041750 | berg04 | 16020.85 | 1344.48 | 60S ribosomal protein L32, putative | No |
| 109 | PBANKA_092210 | berg09 | 15083.73 | 1338.22 | 40S ribosomal protein S18, putative | No |
| 110 | PBANKA_111300 | berg11 | 15944.11 | 1312.56 | purine nucleotide phosphorylase, putative | No |
| 111 | PBANKA_103940 | berg10 | 24073.64 | 1299.39 | 40S ribosomal protein S31/UBI, putative | No |
| 112 | PBANKA_132340 | berg13 | 26093.73 | 1288.86 | conserved Plasmodium protein, unknown function | No |
| 113 | PBANKA_144950 | berg14 | 15729.33 | 1274.07 | conserved Plasmodium protein, unknown function | No |
| 114 | PBANKA_061920 | berg06 | 29910.32 | 1256.79 | secreted ookinete protein, putative (PSOP1) | Yes |
| 115 | PBANKA_060960 | berg06 | 14532.43 | 1252.58 | heat shock protein 20, putative | Yes |
| 116 | PBANKA_123180 | berg12 | 16271.78 | 1246.27 | 60S ribosomal protein L12, putative | No |
| 117 | PBANKA_136270 | berg13 | 11117.52 | 1245.71 | conserved Plasmodium protein, unknown function | Yes |
| 118 | PBANKA_010700 | berg01 | 8460.06 | 1245.48 | conserved Plasmodium protein, unknown function | Yes |
| 119 | PBANKA_130500 | berg13 | 14160.49 | 1236.37 | conserved Plasmodium protein, unknown function | No |
| 120 | PBANKA_040530 | berg04 | 25064.55 | 1232.99 | 40S ribosomal protein S23, putative | No |
| 121 | PBANKA_070910 | berg07 | 12684.18 | 1212.45 | 60S ribosomal protein L22, putative | No |
| 122 | PBANKA_135280 | berg13 | 13020.74 | 1204.65 | conserved Plasmodium protein, unknown function | No |
| 123 | PBANKA_031560 | berg03 | 9348.49 | 1200.95 | 40S ribosomal protein S30, putative | No |
| 124 | PBANKA_122040 | berg12 | 3900.07 | 1191.82 | conserved Plasmodium protein, unknown function | No |
| 125 | PBANKA_142330 | berg14 | 14275.32 | 1184.85 | DNA/RNA-binding protein Alba 1, putative (ALBA1) | No |
| 126 | PBANKA_011080 | berg01 | 6993.99 | 1175.51 | conserved Plasmodium protein, unknown function | No |
| 127 | PBANKA_020820 | berg02 | 8308.45 | 1171.84 | conserved Plasmodium protein, unknown function | No |
| 128 | PBANKA_123360 | berg12 | 23352.02 | 1160.06 | secreted ookinete protein, putative (PSOP13) | No |
| 129 | PBANKA_131290 | berg13 | 3008.96 | 1152.3 | conserved Plasmodium protein, unknown function | No |
| 130 | PBANKA_114380 | berg11 | 14485.74 | 1141.26 | conserved Plasmodium protein, unknown function | Yes |
| 131 | PBANKA_142350 | berg14 | 14100 | 1140.57 | 60S ribosomal protein L13-2, putative | No |
| 132 | PBANKA_123420 | berg12 | 9465.87 | 1136.41 | 40S ribosomal protein S24, putative | No |
| 133 | PBANKA_145690 | berg14 | 18046.1 | 1131.52 | translation initiation factor SUI1, putative | No |
| 134 | PBANKA_110760 | berg11 | 19821.74 | 1129.33 | 6-cysteine protein (P38) | Yes |
| 135 | PBANKA_131130 | berg13 | 12023.37 | 1112.38 | conserved Plasmodium protein, unknown function | Yes |
| 136 | PBANKA_051290 | berg05 | 8524.73 | 1099.8 | conserved Plasmodium protein, unknown function | No |
| 137 | PBANKA_144400 | berg14 | 9065.86 | 1099.29 | macrophage migration inhibitory factor (MIF) | No |
| 138 | PBANKA_123480 | berg12 | 19409.45 | 1066.67 | 40S ribosomal protein S9, putative | No |
| 139 | PBANKA_092440 | berg09 | 12539.21 | 1056.7 | splicing factor, putative | No |
| 140 | PBANKA_111350 | berg11 | 10834.92 | 1055.7 | Rab1a, putative | Yes |
| 141 | PBANKA_061940 | berg06 | 8624.21 | 1031.27 | conserved Plasmodium protein, unknown function | No |
| 142 | PBANKA_143220 | berg14 | 10817.11 | 1014.74 | male development gene 1 (MDV1) | No |
| 143 | PBANKA_091540 | berg09 | 1475.1 | 991.71 | conserved Plasmodium protein, unknown function | Yes |
| 144 | PBANKA_122120 | berg12 | 13067.4 | 980.98 | 60S ribosomal protein L34a, putative | No |
| 145 | PBANKA_145120 | berg14 | 11331.74 | 979.5 | conserved Plasmodium protein, unknown function | Yes |
| 146 | PBANKA_070900 | berg07 | 8222.47 | 966.05 | protein-transport protein sec61 beta 1 subunit, putative | No |
| 147 | PBANKA_113010 | berg11 | 15428.03 | 948.68 | Transcription factor Tfb4, putative | No |
| 148 | PBANKA_081420 | berg08 | 12133.48 | 948.53 | elongation factor 1-beta, putative | No |
| 149 | PBANKA_134200 | berg13 | 19595.33 | 947.76 | Qa-SNARE protein, putative | Yes |
| 150 | PBANKA_136520 | berg13 | 13902.44 | 947.29 | thioredoxin-related protein, putative | No |
| 151 | PBANKA_071260 | berg07 | 23270.81 | 943.73 | 14-3-3 protein, putative | No |
| 152 | PBANKA_041290 | berg04 | 88594.79 | 937.49 | circumsporozoite- and TRAP-related protein (CTRP) | No |
| 153 | PBANKA_120200 | berg12 | 12549.31 | 907.19 | membrane skeletal protein, putative | Yes |
| 154 | PBANKA_134020 | berg13 | 3500.82 | 905.23 | U6 snRNA-associated Sm-like protein LSm6, putative (LSM6) | Yes |
| 155 | PBANKA_136020 | berg13 | 5985.03 | 900.83 | conserved Plasmodium protein, unknown function | No |
| 156 | PBANKA_060710 | berg06 | 3388.38 | 899.21 | tim10 homologue, putative | No |
| 157 | PBANKA_123390 | berg12 | 5570.52 | 898.81 | mitochondrial ribosomal protein L14 precursor, putative | No |
| 158 | PBANKA_122170 | berg12 | 7670.42 | 894.25 | conserved Plasmodium protein, unknown function | Yes |
| 159 | PBANKA_134850 | berg13 | 10881.86 | 888.57 | conserved Plasmodium protein, unknown function | Yes |
| 160 | PBANKA_030350 | berg03 | 8567.14 | 881.58 | conserved Plasmodium protein, unknown function | No |
| 161 | PBANKA_080540 | berg08 | 3004.81 | 865.77 | DNA-directed RNA polymerase II, putative | No |
| 162 | PBANKA_114240 | berg11 | 6368.29 | 856.28 | nucleoside diphosphate kinase b, putative | Yes |
| 163 | PBANKA_030440 | berg03 | 9948.93 | 855.08 | merozoite surface protein 4/5 (MSP4/5) | No |
| 164 | PBANKA_132880 | berg13 | 7661.87 | 853.77 | conserved Plasmodium protein, unknown function | No |
| 165 | PBANKA_040320 | berg04 | 14365.66 | 849.68 | circumsporozoite (CS) protein (CSP) | No |
| 166 | PBANKA_083240 | berg08 | 12225.63 | 842.52 | conserved Plasmodium protein, unknown function | Yes |
| 167 | PBANKA_103550 | berg10 | 8009.4 | 841.36 | vacuolar sorting protein 29, putative | No |
| 168 | PBANKA_145110 | berg14 | 11053.55 | 831.86 | conserved Plasmodium protein, unknown function | No |
| 169 | PBANKA_090640 | berg09 | 9532.17 | 829.87 | 60S ribosomal protein L35ae, putative | No |
| 170 | PBANKA_101860 | berg10 | 6638.64 | 826.51 | 60S ribosomal protein L21e, putative | No |
| 171 | PBANKA_052480 | berg05 | 4667.96 | 811.62 | early transcribed membrane protein (SEP1) | No |
| 172 | PBANKA_111930 | berg11 | 6888.08 | 799.95 | conserved Plasmodium protein, unknown function | No |
| 173 | PBANKA_145930 | berg14 | 14851.18 | 794.52 | actin I | No |
| 174 | PBANKA_124320 | berg12 | 4054.47 | 793.93 | conserved Plasmodium protein, unknown function | Yes |
| 175 | PBANKA_111050 | berg11 | 6760.14 | 792.7 | translationally controlled tumor protein homolog, putative | No |
| 176 | PBANKA_123950 | berg12 | 5456.83 | 780.56 | mitochondrial import receptor subunit, putative | No |
| 177 | PBANKA_061710 | berg06 | 10498.61 | 776.57 | 60S ribosomal protein L11a, putative | No |
| 178 | PBANKA_114170 | berg11 | 9961.55 | 774.73 | 60S ribosomal protein L40/UBI, putative | No |
| 179 | PBANKA_140060 | berg14 | 58651.43 | 771.98 | cytoadherence linked asexual protein, putative | No |
| 180 | PBANKA_135190 | berg13 | 8765.02 | 761.99 | 60S ribosomal protein L6-2, putative | No |
| 181 | PBANKA_142280 | berg14 | 10575.13 | 735.48 | conserved Plasmodium protein, unknown function | Yes |
| 182 | PBANKA_124060 | berg12 | 10739.87 | 726.89 | membrane skeletal protein, putative | No |
| 183 | PBANKA_142360 | berg14 | 9921.77 | 714.68 | 40S ribosomal protein S16, putative | No |
| 184 | PBANKA_143980 | berg14 | 5993.23 | 709.65 | splicing factor 3b subunit, putative (SF3B14) | No |
| 185 | PBANKA_122970 | berg12 | 5139.08 | 700.34 | conserved Plasmodium protein, unknown function | No |
| 186 | PBANKA_040310 | berg04 | 8467.15 | 696.09 | 1-cys-glutaredoxin-like protein-1, putative (GLP1) | Yes |
| 187 | PBANKA_040300 | berg04 | 5570.01 | 694.89 | 60S ribosomal protein L44, putative | No |
| 188 | PBANKA_050360 | berg05 | 6230.17 | 692.96 | 60S ribosomal protein L30e, putative | No |
| 189 | PBANKA_136420 | berg13 | 15321.07 | 691.3 | 60S ribosomal protein L17, putative | Yes |
| 190 | PBANKA_071300 | berg07 | 3342.37 | 687.88 | conserved Plasmodium protein, unknown function | No |
| 191 | PBANKA_082140 | berg08 | 11924.79 | 686.52 | ADP-ribosylation factor-like protein | No |
| 192 | PBANKA_040190 | berg04 | 7335.6 | 675.58 | DNA-directed RNA polymerase subunit I, putative | No |
| 193 | PBANKA_100990 | berg10 | 15880.72 | 675.26 | conserved Plasmodium protein, unknown function | Yes |
| 194 | PBANKA_132430 | berg13 | 5050 | 674.52 | conserved Plasmodium protein, unknown function | Yes |
| 195 | PBANKA_080760 | berg08 | 12908.98 | 671.03 | 40S ribosomal protein S11, putative | No |
| 196 | PBANKA_113800 | berg11 | 6592.9 | 668.2 | conserved Plasmodium protein, unknown function | No |
| 197 | PBANKA_132440 | berg13 | 12700.8 | 665.93 | 60S ribosomal protein L27, putative | No |
| 198 | PBANKA_133830 | berg13 | 12349.43 | 659.51 | 60S ribosomal protein L6, putative | No |
| 199 | PBANKA_070330 | berg07 | 6904.57 | 650.74 | conserved Plasmodium protein, unknown function | No |
| 200 | PBANKA_070390 | berg07 | 16517.75 | 647.72 | receptor for activated C kinase, putative (RACK) | No |
| 201 | PBANKA_123130 | berg12 | 21946.88 | 644.94 | metabolite/drug transporter, putative | Yes |
| 202 | PBANKA_112070 | berg11 | 8363.63 | 644.66 | nascent polypeptide associated complex alpha chain, putative | No |
| 203 | PBANKA_111770 | berg11 | 9961.69 | 639.86 | malate dehydrogenase, putative (MDH) | No |
| 204 | PBANKA_092240 | berg09 | 3140.59 | 639.82 | conserved Plasmodium protein, unknown function | Yes |
| 205 | PBANKA_145230 | berg14 | 9987.54 | 634.12 | conserved Plasmodium protein, unknown function | No |
| 206 | PBANKA_010510 | berg01 | 8009.69 | 630.22 | conserved Plasmodium protein, unknown function | No |
| 207 | PBANKA_110540 | berg11 | 5069.26 | 627.25 | small ubiquitin-related modifier, putative (SUMO) | No |
| 208 | PBANKA_051910 | berg05 | 9168.58 | 626.85 | CCAT-binding transcription factor-like protein, putative | No |
| 209 | PBANKA_093770 | berg09 | 8139.94 | 623.45 | apicoplast ribosomal protein L36e precursor, putative | No |
| 210 | PBANKA_132640 | berg13 | 12884.55 | 623.18 | glyceraldehyde-3-phosphate dehydrogenase, putative | No |
| 211 | PBANKA_134840 | berg13 | 5128.97 | 619.44 | histone H3 variant, putative (CenH3) | No |
| 212 | PBANKA_010060 | berg01 | 4786.15 | 599.58 | schizont membrane associated cytoadherence protein (SMAC) | No |
| 213 | PBANKA_113100 | berg11 | 11112.59 | 597.15 | V-type ATPase, putative | No |
| 214 | PBANKA_142270 | berg14 | 5889.73 | 596.93 | fe-superoxide dismutase, putative | No |
| 215 | PBANKA_071780 | berg07 | 8754.31 | 596.5 | 60S ribosomal protein L15, putative | Yes |
| 216 | PBANKA_081540 | berg08 | 13127.14 | 591.42 | conserved Plasmodium protein, unknown function | No |
| 217 | PBANKA_041720 | berg04 | 9380.58 | 591.24 | conserved Plasmodium protein, unknown function | Yes |
| 218 | PBANKA_080530 | berg08 | 3001.44 | 587.73 | conserved Plasmodium protein, unknown function | No |
| 219 | PBANKA_091670 | berg09 | 7639.07 | 583.61 | conserved Plasmodium protein, unknown function | Yes |
| 220 | PBANKA_041830 | berg04 | 7117.28 | 583.53 | retrieval receptor for endoplasmic reticulum membrane proteins, putative | No |
| 221 | PBANKA_143060 | berg14 | 8099.8 | 580.68 | conserved Plasmodium protein, unknown function | No |
| 222 | PBANKA_010550 | berg01 | 6244.65 | 580.41 | glutaredoxin-like protein (GLP2) | No |
| 223 | PBANKA_133010 | berg13 | 11655.95 | 579.99 | large ribosomal subunit assembling protein, putative | No |
| 224 | PBANKA_142290 | berg14 | 11289.57 | 577.92 | conserved Plasmodium protein, unknown function | Yes |
| 225 | PBANKA_123170 | berg12 | 8916.81 | 577.04 | 60S ribosomal protein L8, putative | No |
| 226 | PBANKA_142950 | berg14 | 7559.83 | 571.06 | RNA polymerase subunit 8c, putative | No |
| 227 | PBANKA_121910 | berg12 | 8604.51 | 570.24 | heat shock protein 90, putative | Yes |
| 228 | PBANKA_082880 | berg08 | 8438.5 | 567.95 | cytochrome c oxidase, putative | No |
| 229 | PBANKA_131740 | berg13 | 12811.2 | 567.06 | co-chaperone p23, putative | No |
| 230 | PBANKA_133960 | berg13 | 11943.29 | 563.25 | MSF1-like protein, putative | Yes |
| 231 | PBANKA_135320 | berg13 | 2149.91 | 563.14 | conserved Plasmodium protein, unknown function | No |
| 232 | PBANKA_010770 | berg01 | 16600.08 | 560.5 | permease, putative | Yes |
| 233 | PBANKA_030320 | berg03 | 5123.63 | 558.59 | DNA-directed RNA polymerase II 16 kDa subunit, putative | 2 |
| 234 | PBANKA_090410 | berg09 | 10661.75 | 556.61 | Rab6 | No |
| 235 | PBANKA_135440 | berg13 | 7538.67 | 552.23 | 60S ribosomal protein L18, putative | No |
| 236 | PBANKA_132490 | berg13 | 5007.95 | 548.94 | mitochondrial ribosomal protein S4/S9 precursor, putative | No |
| 237 | PBANKA_070940 | berg07 | 6798.75 | 547.04 | conserved Plasmodium protein, unknown function | No |
| 238 | PBANKA_052090 | berg05 | 8685.89 | 545.75 | antigen UB05, putative | No |
| 239 | PBANKA_070490 | berg07 | 7833.77 | 544.82 | conserved Plasmodium protein, unknown function | Yes |
| 240 | PBANKA_101310 | berg10 | 7720.9 | 540.08 | 60S ribosomal protein L14, putative | No |
| 241 | PBANKA_122220 | berg12 | 3497.11 | 539.79 | conserved rodent malaria protein, unknown function | Yes |
| 242 | PBANKA_092280 | berg09 | 3313.26 | 538.91 | small nuclear ribonucleoprotein D1, putative (SNRPD1) | Yes |
| 243 | PBANKA_145770 | berg14 | 20639.97 | 537.14 | conserved Plasmodium protein, unknown function | Yes |
| 244 | PBANKA_070110 | berg07 | 6020.35 | 535.69 | conserved Plasmodium protein, unknown function | No |
| 245 | PBANKA_020340 | berg02 | 3712.98 | 534.91 | human hepatopoietin-like protein, putative | Yes |
| 246 | PBANKA_145630 | berg14 | 6237.95 | 531.6 | mitochondrial import inner membrane translocase subunit, putative | No |
| 247 | PBANKA_134910 | berg13 | 8820.58 | 529.47 | merozoite surface protein 7 (MSP7) | No |
| 248 | PBANKA_092000 | berg09 | 3579.42 | 526.96 | multiprotein bridging factor type 1, putative | No |
| 249 | PBANKA_050630 | berg05 | 8023.55 | 525.98 | conserved Plasmodium protein, unknown function | No |
| 250 | PBANKA_040390 | berg04 | 5835.9 | 518.52 | conserved Plasmodium protein, unknown function | No |
| 251 | PBANKA_120070 | berg12 | 10973.21 | 514.69 | conserved Plasmodium protein, unknown function | Yes |
| 252 | PBANKA_131820 | berg13 | 4126.44 | 513.74 | nifU protein, putative | No |
| 253 | PBANKA_071880 | berg07 | 8464.36 | 511.64 | small GTP-binding protein sar1, putative (SAR1) | No |
| 254 | PBANKA_031570 | berg03 | 3391.51 | 510.47 | conserved Plasmodium protein, unknown function | No |
| 255 | PBANKA_093350 | berg09 | 11006.5 | 509.54 | rhomboid protease ROM1 (ROM1) | No |
| 256 | PBANKA_113770 | berg11 | 8810.56 | 508.2 | cytochrome c oxidase subunit 2, putative | No |
| 257 | PBANKA_132590 | berg13 | 1851.08 | 504.52 | conserved Plasmodium protein, unknown function | No |
| 258 | PBANKA_103390 | berg10 | 8220.16 | 503.93 | 40S ribosomal protein S8e, putative | No |
| 259 | PBANKA_062100 | berg06 | 5513.62 | 500.92 | conserved Plasmodium protein, unknown function | Yes |
| 260 | PBANKA_142370 | berg14 | 8983.03 | 500.49 | GDP-mannose 4,6-dehydratase, putative | No |
| 261 | PBANKA_145610 | berg14 | 6475.68 | 498.5 | 40S ribosomal protein S17, putative | No |
| 262 | PBANKA_122280 | berg12 | 11191.68 | 493.93 | tyrosyl-tRNA synthetase, putative | No |
| 263 | PBANKA_103030 | berg10 | 6165.19 | 493.43 | nuclear transport factor 2, putative | No |
| 264 | PBANKA_010450 | berg01 | 6311.68 | 489.62 | conserved Plasmodium protein, unknown function | Yes |
| 265 | PBANKA_121560 | berg12 | 10307.49 | 486.11 | 40S ribosomal protein S3A, putative | No |
| 266 | PBANKA_102890 | berg10 | 11368.41 | 480.69 | diphthamide synthesis protein, putative | No |
| 267 | PBANKA_103660 | berg10 | 8303.03 | 478.47 | ribonucleotide reductase small subunit, putative | No |
| 268 | PBANKA_144110 | berg14 | 6840.37 | 477.38 | hydrolase, putative | No |
| 269 | PBANKA_131230 | berg13 | 5323.05 | 477.16 | SNARE protein, putative | Yes |
| 270 | PBANKA_120440 | berg12 | 5019.7 | 473.83 | DNA/RNA-binding protein Alba 3, putative (ALBA3) | No |
| 271 | PBANKA_132930 | berg13 | 9921.19 | 471.19 | 40S ribosomal protein S3, putative | No |
| 272 | PBANKA_111060 | berg11 | 4241.54 | 469.18 | conserved Plasmodium protein, unknown function | No |
| 273 | PBANKA_114180 | berg11 | 2204.79 | 468.09 | conserved Plasmodium protein, unknown function | No |
| 274 | PBANKA_120940 | berg12 | 7755.27 | 466.45 | conserved Plasmodium protein, unknown function | Yes |
| 275 | PBANKA_103510 | berg10 | 9731.11 | 464.35 | fibrillarin, putative (NOP1) | No |
| 276 | PBANKA_102750 | berg10 | 4722.64 | 462.38 | RNA polymerase small subunit, putative | No |
| 277 | PBANKA_111690 | berg11 | 5064.12 | 461.47 | conserved Plasmodium protein, unknown function | No |
| 278 | PBANKA_082920 | berg08 | 5113.08 | 460.38 | conserved Plasmodium protein, unknown function | Yes |
| 279 | PBANKA_136360 | berg13 | 6247.53 | 459.88 | conserved Plasmodium protein, unknown function | Yes |
| 280 | PBANKA_061930 | berg06 | 5074.26 | 459.62 | conserved Plasmodium protein, unknown function | No |
| 281 | PBANKA_072090 | berg07 | 13454.93 | 458.66 | conserved Plasmodium protein, unknown function | Yes |
| 282 | PBANKA_142170 | berg14 | 8220.52 | 456.75 | secreted ookinete protein, putative (PSOP20) | No |
| 283 | PBANKA_140680 | berg14 | 3315.49 | 455.93 | 40S ribosomal protein S27, putative | No |
| 284 | PBANKA_110340 | berg11 | 6517.89 | 455.4 | 60S ribosomal protein L31, putative | No |
| 285 | PBANKA_121080 | berg12 | 8661.98 | 452.99 | hypoxanthine-guanine phosphoribosyltransferase, putative (HGPRT) | No |
| 286 | PBANKA_071240 | berg07 | 5819.97 | 450.89 | nucleolar preribosomal assembly protein, putative | No |
| 287 | PBANKA_134920 | berg13 | 6360.37 | 450.11 | MSP7-like protein (MSRP1) | No |
| 288 | PBANKA_090650 | berg09 | 5949.55 | 446.64 | 60S ribosomal protein L28, putative | No |
| 289 | PBANKA_135900 | berg13 | 13481.47 | 443.81 | sec61 alpha subunit, putative | No |
| 290 | PBANKA_093000 | berg09 | 3982.39 | 441.32 | clathrin assembly protein AP19, putative | No |
| 291 | PBANKA_010500 | berg01 | 5955.8 | 441.09 | ubiquitin-conjugating enzyme e2, putative | No |
| 292 | PBANKA_011150 | berg01 | 2091 | 440.83 | conserved Plasmodium protein, unknown function | No |
| 293 | PBANKA_061750 | berg06 | 5419.64 | 440.76 | 3'-5' exoribonuclease Csl4 homolog, putative | Yes |
| 294 | PBANKA_130860 | berg13 | 10565.46 | 440.58 | fructose-bisphosphate aldolase 2 (ALDO2) | No |
| 295 | PBANKA_146140 | berg14 | 6203.39 | 439 | conserved Plasmodium protein, unknown function | No |
| 296 | PBANKA_060340 | berg06 | 6454.82 | 435.41 | conserved Plasmodium protein, unknown function | Yes |
| 297 | PBANKA_120510 | berg12 | 10547.94 | 434.46 | PPPDE peptidase, putative | No |
| 298 | PBANKA_081690 | berg08 | 3369.81 | 432.9 | conserved Plasmodium protein, unknown function | No |
| 299 | PBANKA_093220 | berg09 | 5658.59 | 431.76 | peptidyl-prolyl cis-trans isomerase, putative | No |
| 300 | PBANKA_131080 | berg13 | 9965.91 | 431.33 | 40S ribosomal protein S2, putative | No |
| 301 | PBANKA_112980 | berg11 | 6001.02 | 430.73 | Tetratricopeptide repeat protein, putative | No |
| 302 | PBANKA_090560 | berg09 | 9241.69 | 423.3 | translation initiation factor eIF-1A, putative | No |
| 303 | PBANKA_133430 | berg13 | 7167.6 | 421.47 | exported protein 2, putative (EXP2) | No |
| 304 | PBANKA_052420 | berg05 | 3238.46 | 418.69 | early transcribed membrane protein (SEP2) | No |
| 305 | PBANKA_101670 | berg10 | 5774.04 | 415.92 | conserved Plasmodium protein, unknown function | No |
| 306 | PBANKA_136300 | berg13 | 4668.97 | 412.42 | small nuclear ribonucleoprotein E, putative (SNRPE) | No |
| 307 | PBANKA_081680 | berg08 | 4452.55 | 411.94 | glycolipid transfer protein, putative | No |
| 308 | PBANKA_061640 | berg06 | 10052.67 | 410.43 | conserved Plasmodium protein, unknown function | Yes |
| 309 | PBANKA_124440 | berg12 | 1768.56 | 410 | conserved Plasmodium protein, unknown function | No |
| 310 | PBANKA_131140 | berg13 | 5052.88 | 408.73 | conserved Plasmodium protein, unknown function | No |
| 311 | PBANKA_094100 | berg09 | 2708.29 | 407.64 | conserved Plasmodium protein, unknown function | No |
| 312 | PBANKA_110390 | berg11 | 7331.88 | 407 | conserved Plasmodium protein, unknown function | Yes |
| 313 | PBANKA_141400 | berg14 | 6176.19 | 405.76 | conserved Plasmodium protein, unknown function | No |
| 314 | PBANKA_111410 | berg11 | 8562.82 | 405.41 | conserved Plasmodium protein, unknown function | No |
| 315 | PBANKA_114580 | berg11 | 5310.62 | 405.21 | conserved rodent malaria protein, unknown function | No |
| 316 | PBANKA_135580 | berg13 | 2964.31 | 404.88 | transcription activator, putative | No |
| 317 | PBANKA_050580 | berg05 | 5267.84 | 403.98 | deoxyribose-phosphate aldolase, putative | Yes |
| 318 | PBANKA_040250 | berg04 | 5485.52 | 402.81 | conserved Plasmodium protein, unknown function | No |
| 319 | PBANKA_134730 | berg13 | 4243.81 | 401.85 | conserved Plasmodium protein, unknown function | Yes |
| 320 | PBANKA_135820 | berg13 | 4801.95 | 398.56 | conserved Plasmodium protein, unknown function | No |
| 321 | PBANKA_141020 | berg14 | 3034.81 | 397.46 | cytochrome c2 precursor,putative | No |
| 322 | PBANKA_090170 | berg09 | 3153.88 | 396.74 | conserved Plasmodium protein, unknown function | No |
| 323 | PBANKA_094320 | berg09 | 4786.9 | 395.68 | casein kinase II beta chain, putative | No |
| 324 | PBANKA_083590 | berg08 | 1425.81 | 393.93 | conserved Plasmodium protein, unknown function | No |
| 325 | PBANKA_101690 | berg10 | 1246.33 | 392.77 | conserved Plasmodium protein, unknown function | No |
| 326 | PBANKA_133860 | berg13 | 7348.53 | 390.37 | 60S ribosomal protein L23a, putative | No |
| 327 | PBANKA_131270 | berg13 | 7125.5 | 390.17 | gamete egress and sporozoite traversal protein (GEST) | Yes |
| 328 | PBANKA_093840 | berg09 | 6632.64 | 390.01 | endoplasmic reticulum-resident calcium binding protein, putative | No |
| 329 | PBANKA_121850 | berg12 | 9577.38 | 388.66 | conserved Plasmodium protein, unknown function | No |
| 330 | PBANKA_131350 | berg13 | 2883.37 | 387.7 | conserved Plasmodium protein, unknown function | No |
| 331 | PBANKA_113500 | berg11 | 2918.51 | 382.23 | conserved Plasmodium protein, unknown function | No |
| 332 | PBANKA_101380 | berg10 | 3270.99 | 381.34 | cytochrome c oxidase subunit II precursor, putative | No |
| 333 | PBANKA_142940 | berg14 | 1755.9 | 376.75 | dynein light chain 1, putative | No |
| 334 | PBANKA_111540 | berg11 | 1573.39 | 373.34 | conserved Plasmodium protein, unknown function | No |
| 335 | PBANKA_060800 | berg06 | 8097.73 | 372.03 | porphobilinogen deaminase, putative | No |
| 336 | PBANKA_071200 | berg07 | 6320.63 | 370.58 | U3 small nucleolar ribonucleoprotein, putative | No |
| 337 | PBANKA_091720 | berg09 | 2661.47 | 370.2 | RNA-binding protein s1, putative | No |
| 338 | PBANKA_102790 | berg10 | 4464.04 | 364.51 | small nuclear ribonucleoprotein-associated protein B, putative (SNRPB) | No |
| 339 | PBANKA_120420 | berg12 | 8536.26 | 362.46 | conserved Plasmodium protein, unknown function | No |
| 340 | PBANKA_030590 | berg03 | 3928.96 | 361.84 | conserved Plasmodium protein, unknown function | No |
| 341 | PBANKA_081100 | berg08 | 4426.1 | 361.42 | small nuclear ribonucleoprotein Sm D3, putative (SNRPD3) | No |
| 342 | PBANKA_143030 | berg14 | 7390.26 | 360.03 | conserved Plasmodium protein, unknown function | No |
| 343 | PBANKA_142430 | berg14 | 7644.61 | 358.01 | conserved Plasmodium protein, unknown function | Yes |
| 344 | PBANKA_082940 | berg08 | 1929.46 | 357.02 | conserved Plasmodium protein, unknown function | No |
| 345 | PBANKA_041060 | berg04 | 4595.01 | 356.91 | 60S ribosomal protein L26, putative | No |
| 346 | PBANKA_082670 | berg08 | 5902.66 | 354.67 | conserved Plasmodium protein, unknown function | No |
| 347 | PBANKA_082190 | berg08 | 6831.96 | 350.62 | ubiquitin conjugating enzyme, putative | No |
| 348 | PBANKA_080720 | berg08 | 3801.64 | 350.11 | conserved Plasmodium protein, unknown function | Yes |
| 349 | PBANKA_051100 | berg05 | 4954.26 | 346.95 | biotin--acetyl-CoA-carboxylase, putative | Yes |
| 350 | PBANKA_093560 | berg09 | 4290.74 | 346.62 | conserved Plasmodium protein, unknown function | No |
| 351 | PBANKA_136100 | berg13 | 4632.02 | 344.73 | step II splicing factor, putative | No |
| 352 | PBANKA_141140 | berg14 | 2003.76 | 343.46 | ubiquitin-like protein nedd8 homologue, putative | No |
| 353 | PBANKA_136550 | berg13 | 6625.66 | 343.23 | intra-erythrocytic P. berghei-induced structures protein 1 (IBIS1) | No |
| 354 | PBANKA_070530 | berg07 | 2579.13 | 342.23 | conserved Plasmodium protein, unknown function | No |
| 355 | PBANKA_081440 | berg08 | 4032.02 | 340.73 | conserved Plasmodium protein, unknown function | No |
| 356 | PBANKA_031200 | berg03 | 4144.09 | 339.76 | conserved Plasmodium protein, unknown function | Yes |
| 357 | PBANKA_071540 | berg07 | 3273.08 | 337.96 | conserved Plasmodium protein, unknown function | No |
| 358 | PBANKA_112400 | berg11 | 6734.34 | 337.87 | sphingomyelin synthase, putative | No |
| 359 | PBANKA_092910 | berg09 | 5309.76 | 337.83 | acyl-CoA-binding protein, putative | Yes |
| 360 | PBANKA_123690 | berg12 | 3888.65 | 335.65 | conserved Plasmodium protein, unknown function | No |
| 361 | PBANKA_093450 | berg09 | 3676.34 | 334 | protein tyrosine phosphatase, putative | No |
| 362 | PBANKA_090890 | berg09 | 4569.76 | 333.13 | vacuolar ATP synthase subunit f, putative | No |
| 363 | PBANKA_114150 | berg11 | 7475.43 | 332.58 | conserved Plasmodium protein, unknown function | No |
| 364 | PBANKA_142910 | berg14 | 2786.24 | 331.21 | conserved Plasmodium protein, unknown function | No |
| 365 | PBANKA_131850 | berg13 | 2829.45 | 329.87 | conserved Plasmodium protein, unknown function | No |
| 366 | PBANKA_030510 | berg03 | 25322.14 | 328.37 | serine repeat antigen 1 (SERA1) | No |
| 367 | PBANKA_051230 | berg05 | 4439.63 | 327.99 | zinc finger, C3HC4 type, putative | No |
| 368 | PBANKA_092350 | berg09 | 5554.55 | 326.62 | nuclear preribosomal assembly protein, putative | No |
| 369 | PBANKA_120690 | berg12 | 11611.36 | 325.56 | tubulin beta chain, putative | No |
| 370 | PBANKA_100500 | berg10 | 1775.35 | 325.52 | conserved Plasmodium protein, unknown function | No |
| 371 | PBANKA_103060 | berg10 | 5301.7 | 325.01 | conserved Plasmodium protein, unknown function | No |
| 372 | PBANKA_142790 | berg14 | 1688.75 | 324.38 | conserved Plasmodium protein, unknown function | No |
| 373 | PBANKA_052270 | berg05 | 9015.35 | 324.31 | alpha tubulin 2 | No |
| 374 | PBANKA_113790 | berg11 | 4420.54 | 324.21 | proliferating cell nuclear antigen, putative (PCNA) | No |
| 375 | PBANKA_113280 | berg11 | 3846.81 | 324.18 | conserved Plasmodium protein, unknown function | No |
| 376 | PBANKA_123370 | berg12 | 5385.87 | 323.62 | conserved Plasmodium protein, unknown function | Yes |
| 377 | PBANKA_083100 | berg08 | 28258.69 | 318.05 | merozoite surface protein 1 (MSP1) | No |
| 378 | PBANKA_011000 | berg01 | 2504.01 | 317.63 | 60S ribosomal protein L39, putative | No |
| 379 | PBANKA_080140 | berg08 | 3210.14 | 317.38 | conserved Plasmodium protein, unknown function | No |
| 380 | PBANKA_061950 | berg06 | 3487.81 | 316.87 | conserved Plasmodium protein, unknown function | No |
| 381 | PBANKA_134380 | berg13 | 4378.93 | 316.55 | transcriptional regulatory protein sir2a (Sir2A) | Yes |
| 382 | PBANKA_093430 | berg09 | 5459.21 | 316.4 | protein tyrosine phosphatase, putative | No |
| 383 | PBANKA_112460 | berg11 | 1513.07 | 314.61 | conserved Plasmodium protein, unknown function | No |
| 384 | PBANKA_070190 | berg07 | 9759.04 | 314.42 | GPI-anchored micronemal antigen, putative (GAMA) | Yes |
| 385 | PBANKA_122650 | berg12 | 4577.71 | 314.4 | proteasome beta-subunit, putative | Yes |
| 386 | PBANKA_102680 | berg10 | 3605.5 | 312.55 | conserved Plasmodium protein, unknown function | No |
| 387 | PBANKA_062070 | berg06 | 7220.12 | 311.38 | conserved Plasmodium protein, unknown function | Yes |
| 388 | PBANKA_112160 | berg11 | 6984.7 | 310.75 | conserved Plasmodium protein, unknown function | No |
| 389 | PBANKA_135270 | berg13 | 4190.34 | 310.72 | conserved Plasmodium protein, unknown function | No |
| 390 | PBANKA_146400 | berg14 | 3465.94 | 309.77 | conserved Plasmodium protein, unknown function | No |
| 391 | PBANKA_092740 | berg09 | 1871.6 | 306.9 | conserved Plasmodium protein, unknown function | No |
| 392 | PBANKA_121810 | berg12 | 48757.11 | 306.83 | oocyst capsule protein (Cap380) | Yes |
| 393 | PBANKA_020500 | berg02 | 2624.55 | 305.98 | conserved Plasmodium protein, unknown function | No |
| 394 | PBANKA_133350 | berg13 | 1726.45 | 305.45 | conserved Plasmodium protein, unknown function | No |
| 395 | PBANKA_134760 | berg13 | 3751.77 | 305.12 | translation initiation factor 6, putative | No |
| 396 | PBANKA_103880 | berg10 | 3187.77 | 304.71 | conserved Plasmodium protein, unknown function | No |
| 397 | PBANKA_090300 | berg09 | 7358.03 | 304.31 | conserved Plasmodium protein, unknown function | No |
| 398 | PBANKA_090120 | berg09 | 3276.47 | 303.13 | conserved Plasmodium protein, unknown function | No |
| 399 | PBANKA_030800 | berg03 | 3405.19 | 302.55 | Rab5a, GTPase, putative | No |
| 400 | PBANKA_041280 | berg04 | 3404.12 | 301.13 | translation initiation factor E4, putative (eIF4E) | No |
| 401 | PBANKA_101950 | berg10 | 8070.91 | 300.89 | 60S ribosomal protein L5, putative | Yes |
| 402 | PBANKA_031520 | berg03 | 1989.77 | 300.24 | small nuclear ribonucleoprotein Sm D2, putative (SNRPD2) | No |
| 403 | PBANKA_122310 | berg12 | 6021.42 | 300.11 | proteasome subunit alpha, putative | No |
| 404 | PBANKA_061770 | berg06 | 2682.12 | 298.32 | conserved Plasmodium protein, unknown function | No |
| 405 | PBANKA_101520 | berg10 | 9743.64 | 298.21 | diphthamide synthesis protein, putative | No |
| 406 | PBANKA_081570 | berg08 | 7246.62 | 297.07 | membrane transporter, putative | No |
| 407 | PBANKA_122580 | berg12 | 3106.44 | 296.94 | peptidyl-prolyl cis-trans isomerase, putative (CYP24) | No |
| 408 | PBANKA_112900 | berg11 | 5434.7 | 295.19 | secreted ookinete protein, putative (PSOP6) | Yes |
| 409 | PBANKA_051850 | berg05 | 2592.79 | 292.14 | ADP-ribosylation factor, putative | No |
| 410 | PBANKA_136250 | berg13 | 4973.7 | 290.77 | conserved Plasmodium protein, unknown function | No |
| 411 | PBANKA_134460 | berg13 | 4492.19 | 288.54 | conserved Plasmodium protein, unknown function | No |
| 412 | PBANKA_093950 | berg09 | 5006.19 | 288.48 | Rad51 homolog, putative | No |
| 413 | PBANKA_071910 | berg07 | 4688.02 | 286.81 | conserved Plasmodium protein, unknown function | Yes |
| 414 | PBANKA_123300 | berg12 | 1876.3 | 286.69 | conserved Plasmodium protein, unknown function | No |
| 415 | PBANKA_121920 | berg12 | 2961.05 | 285.75 | Cg8 protein, putative | No |
| 416 | PBANKA_110570 | berg11 | 4190.47 | 284.89 | 60S ribosomal subunit protein L24, putative | No |
| 417 | PBANKA_060260 | berg06 | 3669.42 | 284.65 | cytochrome c1 heme lyase, putative | No |
| 418 | PBANKA_061130 | berg06 | 2893.6 | 283.3 | conserved Plasmodium protein, unknown function | No |
| 419 | PBANKA_041670 | berg04 | 3168.06 | 283.15 | bacterial histone-like protein, putative (HU) | No |
| 420 | PBANKA_090580 | berg09 | 5298.49 | 282.46 | DnaJ protein, putative | No |
| 421 | PBANKA_082700 | berg08 | 726.09 | 281.63 | conserved Plasmodium protein, unknown function | No |
| 422 | PBANKA_135420 | berg13 | 2545.01 | 281.52 | conserved Plasmodium protein, unknown function | No |
| 423 | PBANKA_102390 | berg10 | 3751.1 | 280.9 | glutathione s-transferase, putative | No |
| 424 | PBANKA_082570 | berg08 | 2391.6 | 279.9 | telomeric repeat binding factor 1, putative | No |
| 425 | PBANKA_121070 | berg12 | 4256.59 | 279.64 | ubiquinol-cytochrome c reductase complex subunit, putative | No |
| 426 | PBANKA_113990 | berg11 | 2490.98 | 279.11 | conserved Plasmodium protein, unknown function | No |
| 427 | PBANKA_133620 | berg13 | 2073.15 | 278.75 | conserved Plasmodium protein, unknown function | No |
| 428 | PBANKA_082640 | berg08 | 3707.96 | 278.36 | zinc-binding protein (Yippee), putative | No |
| 429 | PBANKA_030840 | berg03 | 2822.7 | 277.71 | dolichol-linked oligosaccharide biosynthesis enzyme, putative | Yes |
| 430 | PBANKA_111320 | berg11 | 3289.85 | 277.63 | conserved Plasmodium protein, unknown function | No |
| 431 | PBANKA_121420 | berg12 | 6825.62 | 277.37 | ribonucleotide reductase small subunit, putative | No |
| 432 | PBANKA_122920 | berg12 | 4790.47 | 276.84 | 60S ribosomal protein L19, putative | No |
| 433 | PBANKA_040900 | berg04 | 3564.48 | 276.51 | conserved Plasmodium protein, unknown function | Yes |
| 434 | PBANKA_083200 | berg08 | 3133.99 | 276.02 | unspecified product (Sel2) | No |
| 435 | PBANKA_080350 | berg08 | 4623.31 | 275.34 | ATP synthase subunit C, putative | No |
| 436 | PBANKA_140760 | berg14 | 5230.99 | 275.23 | 60S ribosomal protein L24, putative | No |
| 437 | PBANKA_134010 | berg13 | 4319.3 | 274.81 | L-lactate dehydrogenase (LDH) | No |
| 438 | PBANKA_083520 | berg08 | 2886.4 | 274.6 | transcription factor with AP2 domain(s), putative | No |
| 439 | PBANKA_092890 | berg09 | 2576.8 | 274.01 | conserved protein, unknown function | No |
| 440 | PBANKA_071870 | berg07 | 1206.93 | 273.51 | CDGSH iron-sulfur domain-containing protein, putative | Yes |
| 441 | PBANKA_092330 | berg09 | 7224.46 | 273.38 | conserved Plasmodium protein, unknown function | No |
| 442 | PBANKA_103970 | berg10 | 2540.5 | 272.55 | conserved Plasmodium protein, unknown function | No |
| 443 | PBANKA_113020 | berg11 | 4740.46 | 271.36 | ER lumen protein retaining receptor, putative | No |
| 444 | PBANKA_101600 | berg10 | 3554.3 | 269.5 | conserved Plasmodium protein, unknown function | No |
| 445 | PBANKA_144780 | berg14 | 2123.99 | 269.43 | conserved Plasmodium protein, unknown function | No |
| 446 | PBANKA_070280 | berg07 | 6912.93 | 268.47 | protein disulfide isomerase | No |
| 447 | PBANKA_090780 | berg09 | 2292.58 | 267.79 | conserved Plasmodium protein, unknown function | No |
| 448 | PBANKA_102960 | berg10 | 5004.06 | 267.71 | conserved Plasmodium protein, unknown function | No |
| 449 | PBANKA_070710 | berg07 | 6506.26 | 265.1 | membrane skeletal protein, putative | Yes |
| 450 | PBANKA_120980 | berg12 | 3562.06 | 265.1 | proteasome subunit beta type-5, putative | No |
| 451 | PBANKA_113320 | berg11 | 6790.67 | 264.91 | cdc2-related kinase 2 (CRK2) | Yes |
| 452 | PBANKA_030200 | berg03 | 6260.35 | 262.87 | conserved Plasmodium protein, unknown function | No |
| 453 | PBANKA_031440 | berg03 | 4915.41 | 261.58 | conserved Plasmodium protein, unknown function | No |
| 454 | PBANKA_133070 | berg13 | 3874.33 | 260.47 | organelle ribosomal protein L22/L17 precursor, putative | No |
| 455 | PBANKA_011140 | berg01 | 3250.64 | 256.44 | conserved Plasmodium protein, unknown function | No |
| 456 | PBANKA_103620 | berg10 | 3657.78 | 256.16 | RNA-binding protein, putative | Yes |
| 457 | PBANKA_052390 | berg05 | 5053.43 | 256.09 | glideosome associated protein with multiple membrane spans 2, putative (GAPM2) | No |
| 458 | PBANKA_133800 | berg13 | 2047.73 | 256 | conserved Plasmodium protein, unknown function | No |
| 459 | PBANKA_134110 | berg13 | 4045.25 | 255.76 | conserved Plasmodium protein, unknown function | No |
| 460 | PBANKA_134620 | berg13 | 3379.43 | 254.64 | signal peptidase 21 kDa subunit, putative (SP21) | Yes |
| 461 | PBANKA_061190 | berg06 | 3240.14 | 254.28 | histidine triad protein, putative | No |
| 462 | PBANKA_031150 | berg03 | 8533.77 | 253.74 | conserved Plasmodium protein, unknown function | Yes |
| 463 | PBANKA_141480 | berg14 | 3097.66 | 253.63 | conserved Plasmodium protein, unknown function | No |
| 464 | PBANKA_071630 | berg07 | 3165.06 | 253.32 | conserved Plasmodium protein, unknown function | No |
| 465 | PBANKA_051300 | berg05 | 5124.38 | 253.11 | conserved Plasmodium protein, unknown function | No |
| 466 | PBANKA_051030 | berg05 | 4690.28 | 252.71 | conserved Plasmodium protein, unknown function | No |
| 467 | PBANKA_050430 | berg05 | 2887.54 | 252.48 | flagellar outer arm dynein-associated protein, putative | Yes |
| 468 | PBANKA_082310 | berg08 | 5042.6 | 252.37 | S-adenosylmethionine synthetase, putative | No |
| 469 | PBANKA_111420 | berg11 | 5377.9 | 252.05 | ribose 5-phosphate epimerase, putative | No |
| 470 | PBANKA_071400 | berg07 | 6773.01 | 252.04 | meiotic recombination protein DMC1-like protein, putative | Yes |
| 471 | PBANKA_061840 | berg06 | 4088 | 251.37 | conserved Plasmodium protein, unknown function | No |
| 472 | PBANKA_130350 | berg13 | 2255.22 | 251.3 | conserved Plasmodium protein, unknown function | No |
| 473 | PBANKA_110590 | berg11 | 4195.27 | 251.08 | conserved Plasmodium protein, unknown function | Yes |
| 474 | PBANKA_093030 | berg09 | 3301.1 | 249.99 | GTP-binding nuclear protein, putative | Yes |
| 475 | PBANKA_011110 | berg01 | 4048.3 | 248.93 | 6-cysteine protein (P12p) | No |
| 476 | PBANKA_120900 | berg12 | 3145.5 | 248.47 | eukaryotic translation initiation factor 2, beta, putative | No |
| 477 | PBANKA_113040 | berg11 | 3519.23 | 247.89 | proteasome subunit, putative | No |
| 478 | PBANKA_051170 | berg05 | 8853.08 | 246.74 | topoisomerase, putative | No |
| 479 | PBANKA_131090 | berg13 | 2630.82 | 246.03 | conserved Plasmodium protein, unknown function | No |
| 480 | PBANKA_093440 | berg09 | 2118.31 | 246.01 | conserved Plasmodium protein, unknown function | No |
| 481 | PBANKA_031630 | berg03 | 3844.87 | 245.92 | Plasmodium exported protein, unknown function | No |
| 482 | PBANKA_103310 | berg10 | 6962.28 | 244.78 | conserved Plasmodium protein, unknown function | No |
| 483 | PBANKA_120520 | berg12 | 6919.11 | 244.26 | conserved Plasmodium protein, unknown function | No |
| 484 | PBANKA_101010 | berg10 | 1973.55 | 244.2 | mitochondrial import inner membrane translocase subunit tim17, putative | No |
| 485 | PBANKA_146390 | berg14 | 5327.02 | 244.18 | conserved Plasmodium protein, unknown function | Yes |
| 486 | PBANKA_070690 | berg07 | 2223.46 | 243.72 | conserved Plasmodium protein, unknown function | No |
| 487 | PBANKA_121890 | berg12 | 3026.24 | 243.17 | elongation factor 1 (EF-1), putative | No |
| 488 | PBANKA_124230 | berg12 | 5806.82 | 242.81 | 58 kDa phosphoprotein,heat shock-related protein (HRP) | No |
| 489 | PBANKA_092340 | berg09 | 3469.3 | 242.68 | 60S ribosomal protein L35, putative | No |
| 490 | PBANKA_011200 | berg01 | 7803.05 | 242.5 | syntaxin binding protein, putative | Yes |
| 491 | PBANKA_060850 | berg06 | 3718.76 | 241.95 | SNARE protein, putative | No |
| 492 | PBANKA_122730 | berg12 | 2107.78 | 241.54 | conserved Plasmodium protein, unknown function | Yes |
| 493 | PBANKA_081890 | berg08 | 9053.7 | 241.33 | heat shock protein 70, putative | Yes |
| 494 | PBANKA_071090 | berg07 | 3040.86 | 240.83 | U6 snRNA-associated Sm-like protein LSm3, putative (LSM3) | No |
| 495 | PBANKA_146110 | berg14 | 4600.8 | 240.4 | RNA-binding protein, putative | No |
| 496 | PBANKA_052220 | berg05 | 1428.24 | 240.05 | conserved Plasmodium protein, unknown function | No |
| 497 | PBANKA_051400 | berg05 | 3187.47 | 239.58 | transcription factor, putative | No |
| 498 | PBANKA_110260 | berg11 | 1765.2 | 239.48 | zinc binding protein, putative | No |
| 499 | PBANKA_050710 | berg05 | 3506.8 | 238.95 | CDGSH iron-sulfur domain-containing protein, putative | No |
| 500 | PBANKA_144030 | berg14 | 2270.5 | 238.51 | conserved Plasmodium protein, unknown function | No |

**Table S14. List of primers used in this study**^a^

| Gene | Primer name | Sequence |
| --- | --- | --- |
| ARA | ARA pCen-GFP-F | aaaggtaCCTTATGAAGATATAAACACATTTTTAATG |
|  | ARA pCen-GFP-R | aaagctagcTATTATTATGGTTTTGCCACCTATTGG |
| PBANKA_070710 (IMC1i) | PBANKA_070710-1 | TATGCCTGAATAAAATTATGGCATTCC |
|  | PBANKA_070710-2 | CTCATCTACAAGCATCgtcgacTGGCACGTTAGAAGGATTTGG |
|  | PBANKA_070710-3 | CCTTCAATTTCGgatccactagATGAAGATGCTCGTTATGATG |
|  | PBANKA_070710-4 | CCACCACAACATTTATTCATTATATTGC |
|  | PBANKA_070710-check-F | TATAAGCAATTGCTCGATCAGTAAGG |
|  | PBANKA_070710-check-R | TTATTTAAGCATGGGGAAATGAGTAG |
|  | PBANKA_070710-GFP-F1 | aaactcgagAGGCACCATCTAGTGAAGATTC |
|  | PBANKA_070710-GFP-R1 | aaagctagcTTCACCACCACAACATTTATTC |
|  | PBANKA_070710-GFP-F2 | aaaggatccATATCTACTCATTTCCCCATGC |
|  | PBANKA_070710-GFP-R2 | aaagcggccgcCTGAAAATAATCACATGCACAC |
|  | PBANKA_070710-GFP-check-F | CAATTGAGGAACCGGTTGTTATTAATG |
|  | PBANKA_070710-GFP-check-R | TTATTTAAGCATGGGGAAATGAGTAG |
|  | PBANKA_070710-southern-F | CGTTATGATGAAATTCCAGAAAATGCC |
|  | PBANKA_070710-southern-R | CCACCACAACATTTATTCATTATATTGC |
| PBANKA_020460 (PhIL1) | PBANKA_020460 pCen-GFP-F | aaaggtacCAACACATGGATTTATATAGTTTG |
|  | PBANKA_020460 pCen-GFP-R | aaagctagcCATATTATCTTTAGGGCATTCTTG |
| PBANKA_083040 (PUA17) | PBANKA_083040 Pcen-GFP-F | aaaggtaccGTTCTTACATTCTTAAGAAACTATATCATC |
|  | PBANKA_083040 Pcen-GFP-R | aaagctagcTATACATAATGAATATTCTTTTTTTTGCCTAG |
| PBANKA_092120 (PUA19) | PBANKA_092120 Pcen-GFP-F | aaaggtaccGTTTTCAGATGTTTTCCCTTGC |
|  | PBANKA_092120 Pcen-GFP-R | aaacctaggAAATTTCGAATTATCTCTATATTGATCTTTTCG |
| PBANKA_122540 (IAAP) | PBANKA_122540-1 | AAGCTATTTACCATAACATGTAACG |
|  | PBANKA_122540-2 | CTCATCTACAAGCATCgtcgacAAATCCGTTGAATTCGCTTTCC |
|  | PBANKA_122540-3 | CCTTCAATTTCGgatccactagCGCGAAAGGGGTTATGTTGTG |
|  | PBANKA_122540-4 | ATACTAGTAGAGACTATACTTCATG |
|  | PBANKA_122540-check-F | CCGATACTGTATTTTCTGAAAAGGC |
|  | PBANKA_122540-check-R | TAAACAAAACTTGTTAAAGGGTTGG |
|  | PBANKA_122540 pCen-GFP-F | aaaggtaccCCACAATAAGTCTTATGCATATAG |
|  | PBANKA_122540 pCen-GFP-R | aaacctaggCACAACATAACCCCTTTCGCG |
|  | PBANKA_122540-southern-F | CGCGAAAGGGGTTATGTTGTG |
|  | PBANKA_122540-southern-R | ATACTAGTAGAGACTATACTTCATG |
| PBANKA_131270 (GEST) | PBANKA_131270 pCen-GFP-F | aaaggtaccTTAAGCCATATATGTTCATGTATCTC |
|  | PBANKA_131270 pCen-GFP-R | aaacctaggTTGTTGTATTATTTTTTTCTCTTCCTTTTC |
| PBANKA_071140 (PPLP4) | PBANKA_071140-1 | TATTCACCCCAACTGTCTTATCAAGAC |
|  | PBANKA_071140-2 | CTCATCTACAAGCATCgtcgacTAAGATATGCACTTATTTTGGG |
|  | PBANKA_071140-3 | CCTTCAATTTCGgatccactagGGAGGAAATGAGCACAAATTTG |
|  | PBANKA_071140-4 | TTAAAAAATGAAAGAGAAATCGCCCCG |
|  | PBANKA_071140-check-F | ATTATGTTGGGATGTTTCTTATGTGTC |
|  | PBANKA_071140-check-R | AATTTTTTATGACAAGTGCCATATAGC |
|  | PBANKA_071140-southern-F | GGAGGAAATGAGCACAAATTTG |
|  | PBANKA_071140-southern-R | TTAAAAAATGAAAGAGAAATCGCCCCG |
| PBANKA_041720 (POM2) | PBANKA_041720-1 | GAAATATGAATAATATTCACACTTTATCCTC |
|  | PBANKA_041720-2 | CTCATCTACAAGCATCgtcgacGGGTGAACTTATGACTACAC |
|  | PBANKA_041720-3 | CCTTCAATTTCGgatccactagATCAGTCTTCTGGCAATG |
|  | PBANKA_041720-4 | AACATGTCGATCATGCCGTAGTGC |
|  | PBANKA_041720 check-F | TTGTATAATCCTAATTCACTTCAAAATG |
|  | PBANKA_041720 check-R | AACAACTAGGTGCATAAATAATCAAC |
| PBANKA_080720 (POM7) | PBANKA_080720-1 | GAAATTCCAAATGAGCCTAAAAAACC |
|  | PBANKA_080720-2 | CTCATCTACAAGCATCgtcgacGCTTCCCATCCAAAATGTTGC |
|  | PBANKA_080720-3 | CCTTCAATTTCGgatccactagCACATTGATATATCTCAAACCC |
|  | PBANKA_080720-4 | GCTATAAACAAATAAAAGGAAGATAG |
|  | PBANKA_080720-check-F | CAAAGATAATATAAACGAAGATATCCC |
|  | PBANKA_080720-check-R | AATAATGCCGTTAAATAAATTTTCCC |
| PBANKA_082110 (CS domain protein) | PBANKA_082110-1 | TTATGCCCTTATAACTTGTGTATGTAC |
|  | PBANKA_082110-2 | CTCATCTACAAGCATCgtcgacCTTAGTACAAATTTGTATCACG |
|  | PBANKA_082110-3 | CCTTCAATTTCGgatccactagATGAAGGAAACGAAATGGGAC |
|  | PBANKA_082110-4 | CACAAACAATATATATACTTAATTCTTTC |
|  | PBANKA_082110-check-F | CCTGACTGGTTCATAAAAGAAATGCTCG |
|  | PBANKA_082110-check-R | GGAAATGCATATTTAATTTTATTCATTCC |
| PBANKA_114380 (POM16) | PBANKA_114380-1 | AGTACAATTTATTTACGTGTGTATTGG |
|  | PBANKA_114380-2 | CTCATCTACAAGCATCgtcgacATATACTATGCAAACTTTCGTC |
|  | PBANKA_114380-3 | CCTTCAATTTCGgatccactagTACGAAGAAATACTTCAAGAGG |
|  | PBANKA_114380-4 | AATAACGATGTTCTTAATACTAGTTGG |
|  | PBANKA_114380-check-F | AGCCCAAAAGGAATATATGAAGGTGGC |
|  | PBANKA_114380-check-R | AATCCCATTGCACTACAAAATAATGAG |
| PBANKA_111920 (POS8) | PBANKA_111920-1 | GTGAGCATATGCATTCGGATAAGAAG |
|  | PBANKA_111920-2 | CTCATCTACAAGCATCgtcgacAGGAACAACGATAGGAAAGTAC |
|  | PBANKA_111920-3 | CCTTCAATTTCGgatccactagGCATAGCCTCTAAATTAATGTC |
|  | PBANKA_111920-4 | GCTGCAGAACCTTATGAAACCATTGC |
|  | PBANKA_111920-check-F | GTTAACAAAGATATGGAAGGATGC |
|  | PBANKA_111920-check-R | TTTGAACAAAAAATTGAACCAGCTG |
|  | PBANKA_111920-southern-F | GTGAGCATATGCATTCGGATAAGAAG |
|  | PBANKA_111920-southern-R | AGGAACAACGATAGGAAAGTAC |
|  | PBANKA_111920 pCen-GFP-F | aaaggtaccCGTATCTTTACAAATTGGGTATATTC |
|  | PBANKA_111920 pCen-GFP-R | aaagctagcTAATTTTATCATTATCGTTTTTTTACTTG |
| PBANKA_100640 (POS7) | PBANKA_100640-1 | GGTTTAATAATTGAGTCCACATTTCTAC |
|  | PBANKA_100640-2 | CTCATCTACAAGCATCgtcgacCCATATGTGCTGGTTATTTGTA |
|  | PBANKA_100640-3 | CCTTCAATTTCGgatccactagGAAAAGGAAGACGATGAGTATG |
|  | PBANKA_100640-4 | GTTTATATAGCTAAAAATAGTGGGTAAC |
|  | PBANKA_100640-check-F | TCACTAGAAACAGATTATTCAGCTAC |
|  | PBANKA_100640-check-R | ATGAACAAAATGTGTAACTAGCCAAAC |
| PBANKA_110760 (P38) | PBANKA_110760-1 | GTTTCGATATCTTATCATATGTATGCTTTG |
|  | PBANKA_110760-2 | CTCATCTACAAGCATCgtcgacATGAATCATCTTGCTCATCTTC |
|  | PBANKA_110760-3 | CCTTCAATTTCGgatccactagCATCTTACTATAGATCAAGTAG |
|  | PBANKA_110760-4 | ATTATTGAAGTTTGCCAATGCCAGTAAC |
|  | PBANKA_110760-CF | GATGAACGTTTATATGAACAAAGCTC |
|  | PBANKA_110760-CR | GGATTTATTCGGATTATCAATGCTAC |
| PBANKA_120070 (POS9) | PBANKA_120070-1 | AAGACAAATGAAAATCAGCTTCACG |
|  | PBANKA_120070-2 | CTCATCTACAAGCATCgtcgacGCGGGTAGCGAAGAGTAAATAC |
|  | PBANKA_120070-3 | CCTTCAATTTCGgatccactagGTGAATTTCAAAAATGCATGTG |
|  | PBANKA_120070-4 | GCTTATTCTAAAAATTATTGGGTGG |
|  | PBANKA_120070-check-F | CATTAAAAACGTGTATTAGTTCCTCG |
|  | PBANKA_120070-check-R | CGAAAATACCATACATTAGTGCATAC |
| PBANKA_123320 (CYC3) | PBANKA_123320-1 | GTATAACCTACACAAGAGTAGCTAAGC |
|  | PBANKA_123320-2 | CTCATCTACAAGCATCgtcgacAATCTTTATGTGTCCTTGGAGG |
|  | PBANKA_123320-3 | CCTTCAATTTCGgatccactagGATAAAAATACTACAGCATCTAC |
|  | PBANKA_123320-4 | CATCCATATATTACTCCTAAAACTATATGC |
|  | PBANKA_123320-check-F | AAAACAAAGCAAACCAATTTCAGTCG |
|  | PBANKA_123320-check-R | GAGAAAATTCCCGTTTTATATGCAC |
|  | PBANKA_123320-southern-F | GATAAAAATACTACAGCATCTAC |
|  | PBANKA_123320-southern-R | CATCCATATATTACTCCTAAAACTATATGC |

^a^ Primers used for preparing gene-targeting constructs have names ending with1–4 after the gene ID. Primers for checking integration of targeting constructs in the genome have names ending with check-F and check-R. Primers used for preparing probes for Southern blot analyses have names ending with Southern-F and Southern-R. Centromere plasmid constructs were prepared using primers ending with Pcen-GFP.
